# Local Resource Availability and Subsidy Flow Mediate the Effects of Disturbances in Meta‐Ecosystems

**DOI:** 10.1002/ece3.73516

**Published:** 2026-04-27

**Authors:** Lynn Govaert, Jessica Colombo, Florian Altermatt

**Affiliations:** ^1^ Department of Evolutionary Biology and Environmental Studies University of Zurich Zurich Switzerland; ^2^ Department of Aquatic Ecology Eawag, Swiss Federal Institute of Aquatic Science and Technology Dübendorf Switzerland; ^3^ Department of Evolutionary and Integrative Ecology Leibniz Institute of Freshwater Ecology and Inland Fisheries Berlin Germany

**Keywords:** cross‐ecosystem flow, disturbance, meta‐ecosystem ecology, microcosm experiment, protozoa, subsidy flow

## Abstract

Disturbances can negatively affect multiple levels of biological organization. With human activities increasing the intensity of disturbance regimes, we need to understand when and where adverse effects of disturbance occur. Yet, how landscape heterogeneity (i.e., variation in local resource availability and meta‐ecosystem resource flows) mediates the effects of disturbances remains poorly understood, despite the frequent occurrence of connected meta‐ecosystems in nature. In this study, we assessed how disturbance intensity affects biomass (here: summed bio‐area of all individuals in the community) and community dynamics of freshwater protist communities in connected ecosystems. We used highly replicated experimental microcosms to simulate two‐patch meta‐ecosystems varying in local resource availability (low versus high carbon release) and meta‐ecosystem subsidy flow (movement of detritus and inorganic nutrients). We expected that a high‐quantity flow of allochthonous subsidies would stabilize receiving communities in their response to disturbance and thus mitigate the negative effects of disturbance on community dynamics, but only when local resource availability was low. We found that the effects of disturbance depended on local resource availability and meta‐ecosystem subsidy flow in our experiment. Irrespective of subsidy flow, high‐resource ecosystems were unaffected by disturbance, resulting in overall higher community biomass of protist communities. In contrast, low‐resource ecosystems showed a strong biomass decline in response to increased disturbance. These negative effects were partly mitigated when low‐resource ecosystems received subsidy flow from a high‐resource ecosystem. Our proof‐of‐concept approach confirms that the effects of disturbance can be context dependent. Including spatial heterogeneity in local resource availability and cross‐ecosystem subsidy flow may be essential for understanding disturbance effects on communities. Further consideration of these factors is needed in management aiming to mitigate disturbances in natural ecosystems.

## Graphical abstract

Using highly replicated experimental two‐patch meta‐ecosystems varying in local resource availability, we assessed how disturbance intensity affects biomass and community dynamics in connected ecosystems. Irrespective of subsidy flow, local high‐resource ecosystems were not affected by disturbance, resulting in overall higher biomass of their communities. Contrastingly, local low‐resource ecosystems showed a strong biomass decline and only when receiving subsidy flow from a high‐resource ecosystem could the negative effects of disturbance be partly mitigated.

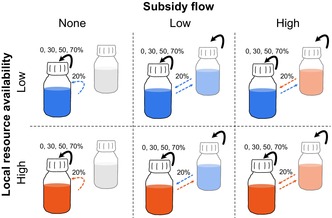

## 1 Introduction

Disturbances can occur as an abrupt event (i.e., ‘pulse’ disturbance) or continuous events (i.e., ‘press’ disturbance) in time that changes the physical environment of organisms (Jentsch and White 2019; Inamine et al. 2022). Such disturbances can rapidly disrupt community structure and the functioning of ecosystems (Jentsch et al. 2009; Wernberg et al. 2013; Jacquet et al. 2020) with direct negative impacts on biodiversity (Keck et al. 2025). Human activities are increasing the frequency and intensity of disturbance events (Hughes et al. 2017; Harris et al. 2018) and also introduce novel disturbance regimes in many natural ecosystems (Turner et al. 2003; Ellison et al. 2005). Freshwater ecosystems are particularly affected by human activities, such as land use changes leading to elevated nutrient and sediment loads, intensive agricultural practices, freshwater salinization and overgrazing (Saunders et al. 2002; Søndergaard and Jeppesen 2007; Dudgeon 2019; Kaushal et al. 2021). Hence, it is important to understand when adverse effects of disturbance occur in order to protect biodiversity in general and freshwater biodiversity in particular (Dornelas 2010; Côte et al. 2023).

Previous studies have shown how disturbances can affect community properties such as species richness (Huston 1979; Haddad et al. 2008; Bongers et al. 2009) and diversity (Britton et al. 2019; Burdon et al. 2020), or how disturbances can alter community biomass size distribution (Jacquet et al. 2020) as well as species coexistence dynamics (Violle et al. 2010; Miller et al. 2011; Fox 2013). However, most of these studies focus on isolated systems, whereas in nature, many ecosystems are linked to neighboring ecosystems via the spatial movement of energy, material or organisms (Polis et al. 1997). For example, terrestrial and aquatic ecosystems are often linked through reciprocal fluxes of nutrients and organic materials, in which plant organic matter falls into streams and in turn aquatic insects emerge from streams, providing a food source for terrestrial predators (Gounand, Little, et al. 2018; Twining et al. 2025). Lakes and rivers experience spatial flows of nutrients and detritus from neighboring forests (Likens 1985), and these flows can be affected by disturbances such as wildfire events (Spencer et al. 2003; Beyers et al. 2005). The recognition that such passive flows of inorganic nutrients and detritus (further referred to as subsidy flow) are not stationary and can influence local community dynamics of neighboring ecosystems has fostered the development of the meta‐ecosystem framework (Loreau et al. 2003; Massol et al. 2011; Gounand, Harvey, et al. 2018).

Despite the increased severity, frequency, and duration of disturbance events (Thom and Seidl 2016; Ummenhofer and Meehl 2017), its effects on community properties and ecosystem dynamics still remain poorly understood (Donohue et al. 2016; Woodward et al. 2016; Philippot et al. 2021), especially in the context of meta‐ecosystems (cf. Osakpolor et al. 2023; Giacomuzzo et al. 2024). Within a meta‐ecosystem framework, disturbance in one ecosystem might affect local community structure in neighboring ecosystems via increased or decreased subsidy exchange, potentially linked to both the quality and quantity of the subsidy (Harvey et al. 2016; Jentsch and White 2019). For example, using microcosm experiments with freshwater protist communities combined with mathematical modeling, Harvey et al. (2016) showed that increased levels of disturbance resulted in a decline of subsidy flow between neighboring heterotrophic and autotrophic ecosystems over time. However, disturbance can also increase or free up available resources (Blöschl et al. 2013; Wright et al. 2015). For example, flooding of a grassland experiment in Jena (Blöschl et al. 2013) increased the available resources with positive effects on local communities (Wright et al. 2015). Floods in rivers can free up available resources by making vulnerable prey more available to top predators (Wootton et al. 1996). Since local community structure can also control the quantity and quality of subsidy outflow to neighboring ecosystems (Gounand et al. 2017), this raises the question of whether an increase in the resource quality of a subsidy inflow can provide community rescue from local disturbance.

Generalizing the effects of disturbance events and how spatial subsidy flows might alter their effects is challenging (but see Jentsch and White (2019) for a general theory of disturbance), as disturbances not only differ in their intensity and frequency (Jacquet et al. 2020) but also depend on the spatial scale and local environmental conditions. Spatial heterogeneity in local environmental conditions (such as resource availability) of ecosystems is common and results in different structure and functioning of their communities (Polis et al. 1997). Such spatial heterogeneity in resource availability might also result in variation in subsidy quality between neighboring ecosystems (Larsen et al. 2016; Osakpolor et al. 2023) which can impact meta‐ecosystem dynamics (Marcarelli et al. 2011). Despite such evidence, theoretical and empirical approaches often focus on equal quality of subsidy flows and local resource availability (Osakpolor et al. 2023), hampering our general understanding of how variation in local resource availability and subsidy flow affects the impact of disturbance.

In this study, we investigated how local resource availability and subsidy flow mitigate the effects of disturbance on biomass dynamics of freshwater microbial communities hosted in microcosms. We experimentally created ecosystems that varied in the amount of local resource availability (i.e., low versus high presence of carbon source) and whether local ecosystems were or were not connected to a neighboring low or high resource ecosystem, respectively, resulting in homogeneous or heterogeneous 2‐patch meta‐ecosystems. We then simulated four levels of disturbance intensities by sampling a certain volume out of each ecosystem, and turning the living microbial species into dead biomass in this sample. When a local ecosystem was connected, it received subsidy inflow from its neighboring ecosystem and provided an equal volume of subsidy outflow. In isolated control systems, the subsidy was retained in the local system. Disturbance and subsidy flow events occurred repeatedly at controlled intervals over a five‐week period. We monitored changes in total biomass and density and biomass size distribution of local freshwater protist communities. Based on Jacquet et al. (2020), we expected overall negative effects of disturbance on community biomass and density with a pronounced shift towards communities dominated by smaller‐sized individuals. However, we expected that a higher local resource availability or a subsidy resource inflow from a high local resource ecosystem would reduce the negative effects of disturbance. Understanding how disturbances influence biotic communities with consequences for ecosystem functioning is crucial for understanding and predicting community changes and will also further improve the design of mitigation efforts for protecting ecosystems against global change.

## 2 Material and Methods

### 2.1 Model System

To test how effects of disturbance on local ecosystem dynamics are shaped by both local resource availability versus meta‐ecosystem subsidy flows, we conducted a microcosm experiment using 11 different unicellular freshwater protists and one rotifer species as a model system (*Blepharisma* sp., *Chilomonas* sp., *Colpidium* sp., *Cyclidium* sp., *Euglena gracilis, Euplotes aediculatus, Loxocephalus* sp., *Paramecium aurelia, Paramecium caudatum, Spirostomum* sp., and *Tetrahymena* sp. as protist species and the rotifer *Cephalodella* sp., subsequently all referred to as “protists”; see also Table S1). These species are known to naturally co‐occur in freshwater ecosystems (McGrady‐Steed et al. 1997), span a wide range of body sizes (10–10^3^ μm) (Giometto et al. 2013), and are easy to maintain and manipulate in the laboratory (Altermatt et al. 2015). These species functionally vary, with most being bacterivorous (*Cephalodella* sp., *Chilomonas* sp., *Colpidium* sp., *Cyclidium* sp., *Loxocephalus* sp., *Paramecium aurelia, Paramecium caudatum*, *Spirostomum teres* , and *Tetrahymena* sp.), some being mixotrophic ( *Euglena gracilis* ), and others ciliate predators (*Blepharisma* sp. and *Euplotes aediculatus* ) (McGrady‐Steed et al. 1997; Harvey et al. 2016). The species used here were chosen based on a previous study by Jacquet et al. (2020). Prior to the experiment, all species were grown to high densities in monocultures using autoclaved protozoan pellet medium and in constant light intensity of 18.21 μmol photons. The protozoan pellet medium was made by adding Protozoan Pellets (0.46 g/L; Carolina Biological Supply, Burlington NC, USA) to local spring water, which was then first autoclaved before being inoculated with 5% of a bacterial culture of *Bacillus subtilis* , *Brevibacillus brevis* , and *Serratia fonticola* . Protozoan Pellets and bacteria provide nutrient and carbon (organic food) for bacteria and protists (Kaunzinger and Morin 1998).

### 2.2 Experimental Design

**FIGURE 1:**
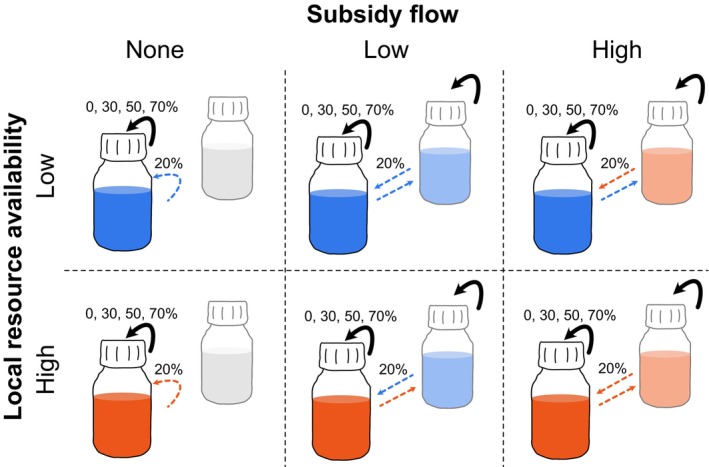
Visualization of experimental design. The experimental design consisted of a factorial design of varying the levels of total disturbance intensity (20%, 50%, 70%, 90%; represented by summing the percentage of black arrows at the top of the experimental bottles and the dashed arrows on the side of the experimental bottles), local resource availability (low or high resource implemented by the number of seeds in the microcosm, blue versus red colored bottles), and meta‐ecosystem flow (i.e., local ecosystem receives no additional subsidy flow or local ecosystem receives subsidy flow from a low or high resource ecosystem) given by the dashed arrows. Disturbance and meta‐ecosystem flow occurred at regular time intervals throughout the experiment. The subsidy flow was reciprocal and corresponded to an exchange of 20% of the volume of the ecosystem, with respective mock handling in the no‐flow treatment.

The experiment was performed using 250‐mL Schott bottles as microcosms. Each local community was established with all 12 protist species using 5 mL per species and culture, and topped up with 40 mL protozoan pellet medium. A schematic representation of the experimental design can be found in Figure 1. We implemented two levels of local resource availability (low versus high) by adding 5 autoclaved wheat seeds per replicate to the high resource treatment level (versus no wheat seed addition in the low resource treatment level). Wheat seeds provide a slow release of carbon, impacting both the nutrient availability for bacteria and protists. Further, we manipulated meta‐ecosystem subsidy flows by manually connecting local ecosystems to a second ecosystem or not. Unconnected ecosystems represent isolated controls (of low and high nutrient availability). Connected meta‐ecosystems consisted of a focal ecosystem of low or high resource availability that was manually connected to a second low or high resource ecosystem. Meta‐ecosystem flow was bi‐directional. There was no species dispersal in any of the treatments. By varying the local resource levels of the two‐patch systems, we created two types of two‐patch ecosystems: homogenous or heterogenous (i.e., both patches low or high resource availability, or one patch of each, respectively) (Figure 1). Finally, we crossed these two treatments with four levels of disturbance intensities (i.e., 0%, 30%, 50% and 70%, detailed below). Treatment combinations were 6‐fold replicated. A meta‐ecosystem subsidy flow of 20% was chosen as a control treatment, as previous work has shown no effect of such medium removal on protozoan communities (Jacquet et al. 2020).

Disturbance and meta‐ecosystem subsidy flow events were implemented at an interval of 4 days, starting 1 week after the initial species inoculation and repeated 8 times until Day 39, when the experiment was terminated. The disturbance events were conducted following Jacquet et al. (2020), whereby the respective volume of medium was extracted through pipetting and microwaved to kill organisms and turn them into detritus (see, e.g., (Harvey et al. 2016)). Given that disturbance and meta‐ecosystem flow events were conducted at the same time, this means that aliquots of 20, 50, 70, or 90 mL out of 100 mL were taken from corresponding ecosystems and microwaved. After cooling down of the aliquot, all (if isolated) or part (if connected) of the aliquot was then added back into the ecosystem from which it was obtained. If the ecosystem was isolated, local ecosystems received 20, 50, 70, or 90 mL from their own ecosystem. If the ecosystem was connected, then it received 20 mL of the microwaved aliquot from its neighboring ecosystem and 0, 30, 50, or 70 mL aliquot from their own ecosystem, corresponding to the disturbance intensity level. All subsidy flow and disturbance treatments were done by manually pipetting the respective aliquots from and to the corresponding ecosystems. In this study, meta‐ecosystem subsidy flow is thus a mixture of terrestrial DOM, POM and inorganic nutrients.

The experiment took place under a constant temperature of 20°C and a constant light intensity of 18.21 μmol photons, and the positions of all experimental microcosms were randomized.

### 2.3 Replication Statement

**Table:** 

Scale of inference	Scale at which the factor of interest is applied	Number of replicates at the appropriate scale
Individual communities	Microcosms of a meta‐ecosystem experimental unit	6 replicates of each combination

### 2.4 Data Collection

To obtain information on densities and biomass values of the experimental communities, all microcosms were sampled every 4 days. The first time point of measurement was taken 1 week after the setup of the experiment, 2 days before the first disturbance event. Using microscope slides and small aluminum plates (height ~0.5 mm), we created a custom‐made chamber (see Altermatt et al. 2015 for details). We sampled 250 μL and recorded a 10 s video of an effective volume of 34.4 μL using a Leica M205C stereomicroscope at a 1.6‐fold magnification and an Orca Flash 4 camera (Hamamatsu). Videos were then processed using ImageJ and the R‐package BEMOVI following (Pennekamp et al. 2015). From these videos, we quantified biomass and density of each community. Biomass of a community was calculated by averaging the sum of the mean bio‐area of all individuals in the community (i.e., sum of all individuals' cell area; μm^2^) across the 250 frames. Density of a community was calculated as the total number of detected individuals per frame divided by the total number of 250 frames. Density was then converted to obtain the number of individuals per mL. To determine the size structure of each community, we categorized the observed individuals into twelve size classes ranging from 0 to 1.5 × 10^4^ μm^2^ with a width of 1250 μm^2^ for each size class (similarly to the approach in Jacquet et al. 2020). The first size class covered all individual cells that had a cell area < 1250 μm^2^. The 12th size class covered all individual cells between 13,750 and 15,000 μm^2^. Prior to statistical analyses, videos with moving medium were omitted (detailed in Table S1).

### 2.5 Statistical Analysis

To understand how disturbance intensity and meta‐ecosystem subsidy flow affect local community dynamics and whether this effect depends on resource availability, we take a local ecosystem approach. The heterogeneous meta‐ecosystems (low resource connected to high resource) can be analyzed from the perspective of the low resource system (as focal community) versus high resource system as focal community. Being the same meta‐ecosystem, but with two different perspectives on the local patch type means that they must be analyzed separately. All other systems (isolated or homogeneous meta‐ecosystems) are only analyzed from one perspective. Therefore, when conducting linear mixed‐effects models, we analyzed the local low‐resource ecosystems separately from the high‐resource ecosystems and then compared the effect of disturbance intensity and subsidy flow.

For an overall assessment on the effect of disturbance intensity and meta‐ecosystem subsidy flow on community biomass and density throughout the experiment for the local low‐ and high‐resource ecosystems, we conducted a linear mixed‐effect model with time as a repeated measure. Community biomass and density were taken as response variables, respectively, disturbance intensity and meta‐ecosystem subsidy flow as fixed predictor variables and replicate as a random factor. Model assumptions of linearity and normality of residuals were checked visually and using a Shapiro–Wilk test. Next, to quantify temporal change and time‐point specific changes, we used log‐response ratio analysis. The log response ratio is calculated as the natural logarithm of the ratio between the treatment and control mean response values. First, to assess temporal changes in community biomass and density, we quantified how community biomass and density at each time point deviated from the first time point of measurement for each experimental combination of local resource availability, meta‐ecosystem flow and disturbance intensity. Hence, for each experimental treatment combination, we took the natural logarithm of the response ratio, which is calculated by dividing the mean community biomass or density at each time point by the mean community biomass or density at the first time point. Second, to assess the effect of disturbance, local resource availability and meta‐ecosystem subsidy flow on community biomass and density at each time point, we quantified how each experimental treatment combination deviated from the community biomass and density from the control, that is, undisturbed isolated low‐resource ecosystem. Hence, for each time point, we took the natural logarithm of the response ratio, which is calculated by dividing the mean community biomass or density of a specific treatment by the mean community biomass or density of the control, that is, undisturbed isolated low‐resource ecosystem.

To determine the effect of disturbance intensity, local resource availability, and meta‐ecosystem subsidy flow on compositional changes in community size structure, we used the classification of individuals into the 12 previously mentioned size classes. Using these size classes, we quantified “size class diversity H” using Shannon's diversity index H calculated using the diversity() function from the vegan R Package (Oksanen et al. 2007) and “size class evenness” calculated as Shannon's diversity index H divided by the natural logarithm of species richness and this for each community at each time point. We also performed a permutational analysis of variance (PERMANOVA) on Hellinger‐transformed community composition using the adonis() function from the vegan R package (Oksanen et al. 2007). Similar to community biomass and density, we quantified log response ratios of size class diversity and evenness to quantify the temporal change for each experimental treatment combination and how each experimental treatment combination deviated from the control, undisturbed isolated low‐resource ecosystems. In addition, we also performed PERMANOVA for the local low‐ and high‐resource ecosystems using the Hellinger‐transformed size structure community composition as response variable and using disturbance intensity, subsidy flow and time as fixed predictor variables and replicate as random effect. We then conducted PERMANOVAs for each time point. Non‐metric Multi‐Dimensional Scaling (NMDS) was done to visualize differences in Hellinger‐transformed community composition using the metaMDS() function from the vegan R package (Oksanen et al. 2007).

## 3 Results

**FIGURE 2:**
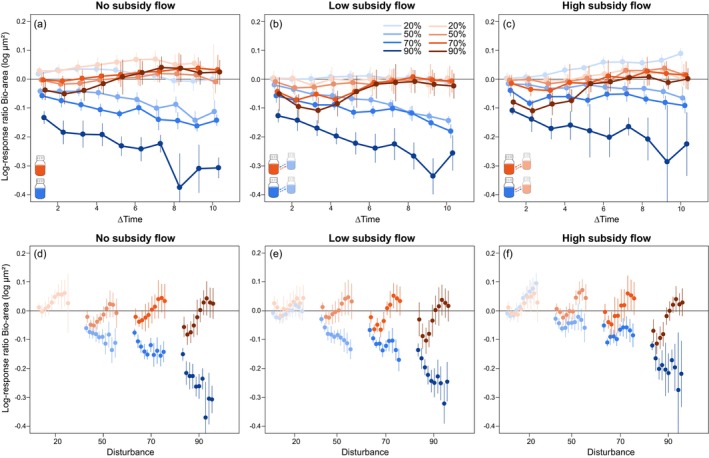
Log response ratios of time series of biomass (calculated as log‐transformed summed bio‐area μm^2^) comparing each time point to T0 for (a) the isolated ecosystems, (b) the low‐resource connected ecosystems and (c) the high‐resource connected ecosystems. Log response ratios of biomass comparing each treatment to the control (here taken as the low‐resource unconnected ecosystem) at each time point for (d) the isolated ecosystems, (e) the low‐resource connected ecosystems and (f) the high‐resource connected ecosystems. Blue symbols are low resource focal systems, while red symbols indicate high resource focal systems, both with varying levels of disturbance intensity (20%, 50%, 70%, 90%), respectively.

Overall, we largely found significant negative effects of disturbance intensity on community biomass for both local low‐ and high‐resource ecosystems (Table S2). A significant positive effect of subsidy flow on community biomass was only found for local low‐resource ecosystems, but only when these ecosystems were connected to a high‐resource ecosystem (Table S2). We found a significant positive interaction effect between disturbance intensity 50% and meta‐ecosystem low subsidy flow for both local low‐resource and local high‐resource ecosystems, whereas a significant positive interaction was found between disturbance intensity 50% and meta‐ecosystem high subsidy flow for local high‐resource ecosystems (Table S2). These results are corroborated by the log response ratio of biomass (Figure 2). The log response ratio analysis across time showed that disturbance had strong negative effects on community biomass that intensified over time for all local low‐resource ecosystems (Figure 2a–c, blue lines; Table S3). These ecosystems showed similar responses: a reduction in biomass that was initially largest for the highest disturbance intensity, with significant differences for 50 and 70 with 90% disturbance intensity throughout most of the experiment (i.e., non‐overlapping 95% confidence intervals). The decreasing trend in community biomass for the local low‐resource ecosystems largely disappeared when these local ecosystems received a meta‐ecosystem high subsidy flow input, but only so for the 50% and 70% disturbance intensity (Figure 2c). For the undisturbed local low‐resource ecosystem connected to a high‐resource ecosystem, we instead found a significant increase in community biomass that became larger over time (Figure 2c; Table S3c). Contrary, only minor effects of disturbance were found for the local high‐resource ecosystems (Figure 2a–c, red lines). For isolated local high‐resource ecosystems, we found a significant increase in community biomass (Figure 2a; Table S3a). In the other experimental treatment combinations, there is often a minor decrease during the first part of the experiment, which disappeared towards the end of the experiment (Table S3).

Log response ratio analysis for community biomass at each time point showed similar findings (Figure 2d–f; Table S4). Local low‐resource ecosystems were significantly impacted by disturbance, showing a significant reduction in community biomass at all time points (Figure 2d,e, blue symbols). When local low‐resource ecosystems received a high‐resource meta‐ecosystem flow, the effects of disturbance could be mitigated with a less strong decrease in community biomass for 50% and 70% disturbance intensity (Figure 2f). Community biomass of local high‐resource ecosystems was overall significantly higher compared to their local low‐resource counterparts (Figure 2d–f, red symbols). However, the positive effect of high resources was mainly observed towards the end of the experiment.

Effects of disturbance intensity and meta‐ecosystem subsidy flow on density were overall similar to the effects found for community biomass (Table S5). Hence, here we only discuss the differences between density and biomass results. For local low‐resource ecosystems, we did not find a significant interaction effect between disturbance intensity 50% and meta‐ecosystem low subsidy flow (Table S5). For local high‐resource ecosystems, the main difference with community biomass was that we observed a significant positive effect of disturbance intensity 70% and 90% on density (Table S5). Log response ratio analysis showed again similar patterns for density compared to community biomass (Supporting Information C: Figure S1). Compared to biomass, local low‐resource ecosystems showed a strong density reduction with 90% disturbance intensity; the reduction in density of the 90% disturbance intensity treatment was similar to the density reduction of the 50% and 70% disturbance intensity treatments in these ecosystems over time (Table S6). In contrast, for local high‐resource ecosystems, we observed that densities in the 90% disturbance intensity were higher compared to the undisturbed local high‐resource ecosystems, whereas such a difference was not found for biomass. Log‐response ratio analysis for densities at each time point showed no distinctive differences compared to the patterns found for community biomass (Figure S1d–f; Table S7).

**FIGURE 3:**
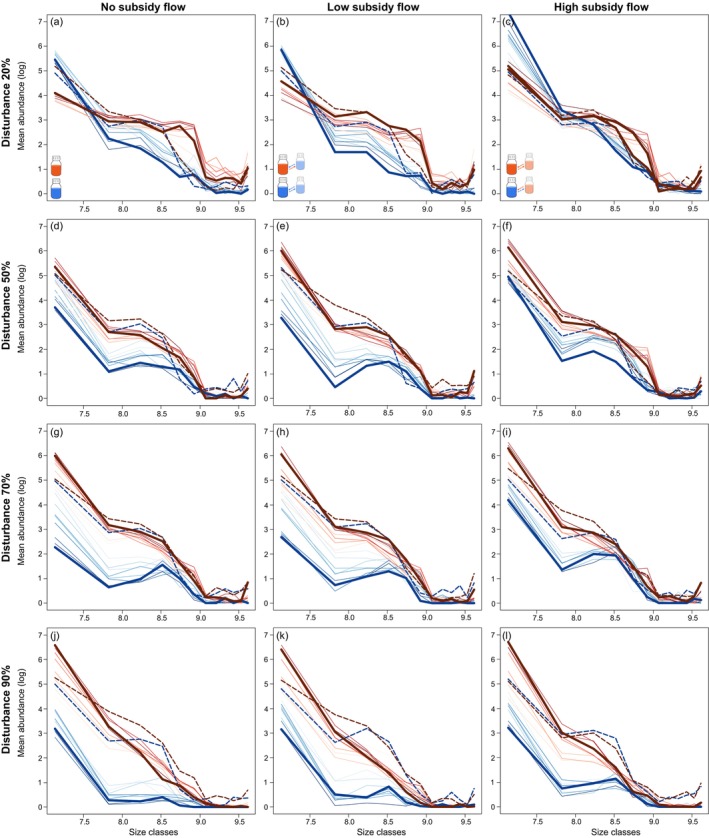
Density plots of community size structure for the four disturbance intensities (from top to bottom: 20%, 50%, 70%, and 90%) over time. Left panels: Isolated local low‐ (blue colors) and high‐resource (red colors) ecosystems. Middle panels: Local low‐ and high‐resource ecosystems connected to a low‐resource ecosystem. Right panels: Local low‐ and high‐resource ecosystems connected to a high‐resource ecosystem. Community size structure at the start of the experiment is indicated with a dashed line. Increasing color intensity of solid lines reflects later time points in the experiment. The thick solid line represents the community size structure at the end of the experiment.

**FIGURE 4:**
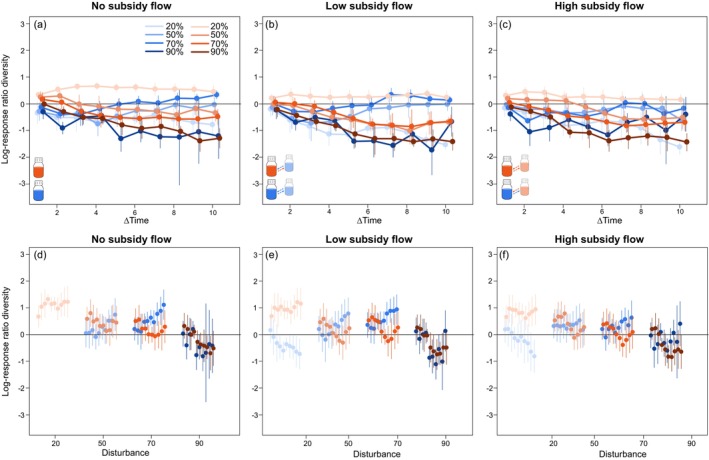
Log response ratios of time series of species diversity (quantified as Shannon's diversity index H) comparing each time point to T0 for (a) the isolated ecosystems, (b) the low‐resource connected ecosystems and (c) the high‐resource connected ecosystems. Log response ratios of evenness comparing each treatment against the control (here taken as the low‐resource isolated ecosystem) at each time point for (d) the isolated ecosystems, (e) the low‐resource connected ecosystems and (f) the high‐resource connected ecosystems. Blue symbols are low resource focal systems, while red symbols indicate high resource focal systems, both with varying levels of disturbance intensity (20%, 50%, 70%, 90%), respectively.

**FIGURE 5:**
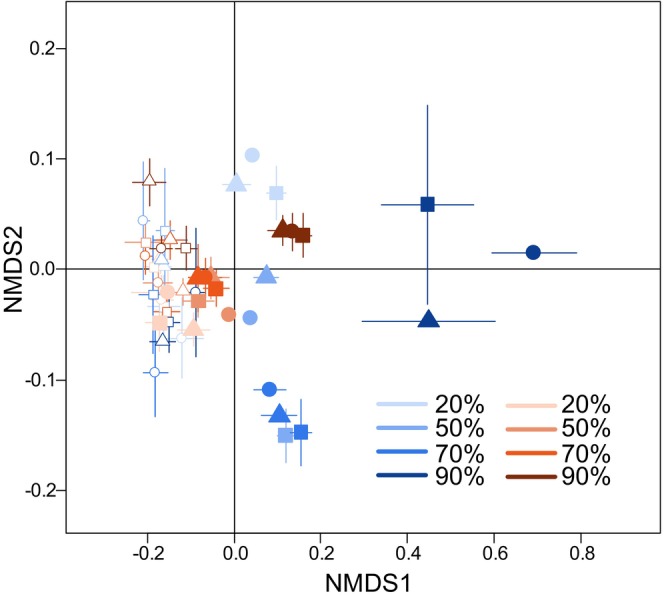
Visualization of community size class composition at the start (empty symbols) and end (filled symbols) of the experiment (for a time series of compositional change see Figure S3). We used Non‐Metric Dimensional Scaling analysis on Hellinger‐transformed size class abundances. Blue and red colors represent focal low‐resource and high‐resource ecosystems, respectively, with circles representing isolated ecosystems, squares representing ecosystems connected to a low‐resource ecosystem and triangles representing ecosystems connected to a high‐resource ecosystem. Color intensity reflects different disturbance intensity levels. Error bars reflect 95% confidence intervals calculated using mean and standard error values assuming normal distribution.

To evaluate compositional changes in the community size structure, we classified individuals into 12 size classes (Figure 3) from which we quantified “size class diversity” using Shannon's diversity index (Figure 4) and “size class evenness” (Figure S2) as well as performed multivariate analysis to assess differences in community size class composition (Figure 5). In all experimental treatments, community composition changed significantly over time (significant effect of Time, *p* < 0.001; Table S8) and this change in community composition significantly differed for different disturbance intensities (significant interaction between Time and Disturbance intensity, *p* < 0.001; Table S8). Subsidy flow significantly altered community composition, but only for those communities from local low‐resource ecosystems (Table S8). The change in community structure also reflected itself in a significant effect of disturbance intensity and a significant interaction between disturbance intensity and subsidy flow on size class diversity and evenness for both local low‐ and high‐resource ecosystems (Tables S9 and S10). Primarily for size class diversity, we also found a significant effect of subsidy flow but only for local high‐resource ecosystems (Table S9). Log response ratio analysis patterns for diversity and evenness were similar. We found that communities from undisturbed local low‐resource ecosystems became significantly less diverse (Figure 4, blue lines; Table S11) and less even (Figure S2, blue lines; Table S12) throughout the experiment, irrespective of the subsidy flow treatment. Disturbed local low‐resource ecosystems showed a significantly lower diversity at the start of the experiment, but this effect disappeared towards the end of the experiment (Figure 4a–c; Table S11). Only for the highest disturbance intensity, communities remained significantly less diverse throughout the experiment compared to the start of the experiment (Table S11). We only observed significant effects of less even communities for those in local low‐resource ecosystems experiencing the highest disturbance intensity (Figure S2a–c; Table S12). Communities from undisturbed local high‐resource ecosystems were significantly more diverse (Figure 4a–c, red lines) and more even (Figure S2a–c, red lines), but not for all time points when these ecosystems were connected (Tables S11 and S12). However, when local high‐resource ecosystems were disturbed, their communities became less diverse and less even, independent of the subsidy flow treatment.

Disturbance had a significant effect on the community size class composition at each time point for communities from both local low‐ and high‐resource ecosystems (Table S13). Only for communities from the local low‐resource ecosystems did we found significant effects of meta‐ecosystem subsidy flow (time points 2, 5, and 8; Table S13) and a significant interaction between disturbance intensity and meta‐ecosystem subsidy flow (time points 4, 5, and 9; Table S13). When comparing experimental treatments to the undisturbed local low‐resource ecosystems at specific time points, we observed no strong effect of subsidy flow on size class diversity and evenness for communities from undisturbed local low‐resource ecosystems (Figure 4d–f, Figure S2d–f, Tables S14 and S15). Visually, we observed the strongest effects of disturbance intensity, where communities from local low‐resource ecosystems could be clearly separated based on disturbance intensity on the NMDS plot (Figure 3). The effect of disturbance disappeared among communities from local high‐resource ecosystems, with only the 90% disturbance intensity being separated from the lower disturbance intensities on the NMDS plot (Figure 3). Compared to undisturbed isolated low‐resource ecosystems, local low‐resource ecosystems that experienced 50% and 70% disturbance intensity harbored more diverse and more even communities, and significantly so at later time points. For the highest disturbance intensity, no significant effects were observed (Figure 4d–f, Figure S2d–f, Tables S14 and S15). Local high resource availability resulted in significantly more diverse and more even communities, and this at all time points of the experiment (Figure 4d–f, Figure S2d–f, Tables S14 and S15). However, this positive effect disappeared with increasing disturbance intensity, with only the communities from the local high‐resource ecosystems experiencing a 50% disturbance intensity initially being significantly more diverse and more even.

## 4 Discussion

Most community ecology theories are based on steady‐state conditions, whereas natural ecosystems and communities often experience a wide range of disturbances from minimal fluctuations to more intense disturbance events (Hastings 2010). In this study, we investigated how the effect of disturbance intensity is altered by local resource availability and subsidy flows in meta‐communities of freshwater unicellular organisms with different functional traits. We created experimental meta‐ecosystems that varied in their local resource availability and subsidy flow. Replicated sets of these meta‐ecosystems were exposed to different levels of disturbance intensity. By following the community dynamics such as biomass, density, and the community structure and size class distribution, we showed in this proof‐of‐principle experiment that disturbance impacted all these variables: Community biomass and density were significantly reduced with higher disturbance intensity. However, these effects were mitigated by local high‐resource availability or by subsidy resource inflow from a neighboring high‐resource ecosystem. Our work shows that local responses to disturbances can be mitigated by local factors such as resource availability but also by cross‐ecosystem flows. Biomass size distribution was also heavily impacted by disturbance intensity, with decreasingly diverse communities in the highest disturbance intensity treatment. Again, these effects were mitigated by local high‐resource availability or by subsidy inflow from a neighboring high‐resource ecosystem. Consequently, local low‐resource ecosystems connected to a high‐resource ecosystem overall experienced beneficial effects of the high‐resource inflow. Our results highlight that the beneficial effects of subsidy flow depend on the local quality of the ecosystem as well as on the quality of the subsidy flow.

The negative impact of disturbance depended strongly on the local resource availability of the ecosystems. A resource‐mediated impact of disturbance has also been found in other studies on fish (Kraus et al. 2016; Burdon et al. 2020) and macroinvertebrate (Gafner and Robinson 2007) communities. For example, fish communities were less impacted by stream pollution when an additional supply of terrestrial prey was present (Kraus et al. 2016; Burdon et al. 2020). In our study, communities from local low‐resource ecosystems experienced the largest declines in community biomass and density when disturbed. In analogy to Jacquet et al. (2020), we observed non‐linear biomass and density declines which intensified with higher disturbance intensity. Yet, the effect of disturbance depended on both the local resource availability and the subsidy flow. Most strikingly, while we also observed an initial decrease in biomass and density at the start of the experiment for most of the local high‐resource ecosystems, in the second half of the experiment, negative effects of disturbance on community biomass and density dynamics were not observed anymore, indicating that local high‐resource availability could counteract these negative effects of disturbance in disturbed local high‐resource ecosystems. Previous studies have shown that nutrient addition can increase productivity of and hence biomass in a community (Subalusky et al. 2018). Nutrients also promote the growth of bacteria, which are the main food source for some of the protist species used in our experiment (Berninger et al. 1991; Holyoak 2000). Hence, in the high‐resource ecosystems that rapidly acquired higher biomass of protists, disturbance could have translated into a higher local detritus production, which benefited the disturbed community most likely by providing an increased bacterial growth in the system. Previous studies also found that nutrient enrichment can increase the resilience of communities to disturbances (Boada et al. 2017) or foster the dominance of resilient species (Polazzo et al. 2022). For example, Boada et al. (2017) found that macrophyte communities were buffered against sea urchin grazing, but only in nutrient‐rich conditions. Nevertheless, many more studies document negative effects of nutrient enrichment in aquatic systems (due to eutrophication) (Smith and Schindler 2009; Jeevannavar et al. 2024), with highly eutrophized environments leading to decreases in species richness and biodiversity (Tilman et al. 2001; Rocke et al. 2016). The nutrient levels chosen in our experiment are most likely below eutrophication thresholds and therefore showed mostly beneficial effects (Holyoak 2000). Aquatic ecosystems in particular experience nutrient addition from neighboring terrestrial ecosystems (Likens and Bormann 1974; Twining et al. 2025) which can vary temporally in quality (Delpierre et al. 2009; Cereghetti et al. 2025), timing (Sato et al. 2016) and duration (Sato et al. 2021; Battin et al. 2023). Understanding the effect of resource‐mediated subsidy flows for species responses to disturbances in connected ecosystems becomes increasingly important, as previous work demonstrates that global change can alter the quality and quantity of these subsidy flows (Zhang et al. 2023; Twining et al. 2025).

Whereas disturbance events are known to affect subsidy flows in experimental (Harvey et al. 2016) and natural (Spencer et al. 2003; Neary et al. 2005) systems, it is still not fully understood if and how spatial subsidy flows modulate the effects of these disturbances. In some cases, disturbance can increase (Wright et al. 2015), free up (Wootton et al. 1996), or alter (Kraus et al. 2016) available resources, which can then affect the quality and/or quantity of subsidy flow to neighboring ecosystems. In addition, the effects of subsidy flow may also depend on the ecosystem size (Giacomuzzo et al. 2024, 2026). Our study found beneficial effects of spatial flows, specifically an increase in local biomass and densities of communities from low‐resource ecosystems, even when they experienced high disturbance (50% and 70%). However, this beneficial effect strongly depended on the resource conditions of the local and neighboring ecosystems, showing the display of an interactive effect between local and neighboring resource availability. Specifically, we only observed a beneficial effect from resource subsidy inflow when local ecosystem conditions are poor and the subsidy inflow is from a high‐quality neighboring ecosystem. No additional beneficial effect was observed for local high‐resource ecosystems or for low‐resource ecosystems with low‐resource subsidy inflow.

Changes in resources also affect the diversity and composition of communities (e.g., bacteria; (Newton and McMahon 2011; Chi et al. 2021), phytoplankton; (Bilbao et al. 2023)). In our study, both local resource availability and subsidy resource inflow affected the structure of the protist communities. Overall, we observed the largest shifts in community structure for low‐resource communities independent of subsidy flow. Low‐resource communities were less diverse and less even. Such lower diversity can affect the stability of these ecosystems (Isbell et al. 2015). Analogous to Jacquet et al. (2020), we observed a larger decline in the abundance of medium‐ and larger‐sized individuals at the highest disturbance intensity, indicating that while the disturbances were not size‐selective per se, they did target larger‐sized (often slower growing) species. The reduction in larger‐sized species (often predatory species) can result in a predation‐release effect for the smaller species, further increasing their abundances (Brose et al. 2006, 2017). However, such decline was not found for the local high‐resource ecosystems independent of subsidy flow and was less severe for local low‐resource ecosystems with subsidy flow from a high‐resource ecosystem. This also resulted in high‐resource communities not showing drastic changes in their community structure (except in the highest disturbance intensity). All these communities maintained a high abundance of smaller‐sized species, which in turn might have been important to maintain the medium‐ and larger‐sized species in the community, despite their slower growth rate.

To conclude, we showed that local resource availability and subsidy flow can mediate the effect of disturbance on experimental freshwater communities. Whereas we found strong negative effects of disturbance on community biomass and size structure (community composition) with potential strong implications for ecosystem stability, these effects of disturbance were mitigated by higher local resource availability and in heterogeneous meta‐ecosystems. The effect of perturbations or disturbances and their impacts on neighboring ecosystems largely depend on the quality of the local ecosystem itself. Given that global change is altering the quantity and quality of subsidy flows between neighboring ecosystems (e.g., aquatic‐terrestrial meta‐ecosystems, Twining et al. 2025), our findings have important implications for understanding community responses to disturbance of communities embedded within connected ecosystems. Our study, in combination with recent evidence that the quality, quantity, timing and duration of resource subsidy flow can have strong effects on neighboring communities (Delpierre et al. 2009; Sato et al. 2016, 2021; Battin et al. 2023; Cereghetti et al. 2025), highlights the need for a spatially explicit study of resource availability and disturbance regimes that connect different ecosystems in space. This has implications for system that are naturally connected through subsidy flows, such as the terrestrial litter input in streams and lakes (Likens and Bormann 1974; Richardson et al. 2010), or the annual mass drownings of the Serengeti wildebeest (Subalusky et al. 2017). It might also have important implications for agricultural catchments in which not only changes in precipitation, but also management practices might alter the import of nutrients into neighboring water bodies (Adrian et al. 2009). It is important to realize that such altered management‐induced disturbances can directly affect dynamics in spatially coupled ecosystems (Richardson et al. 2010; Sitters et al. 2015; Harvey et al. 2016).

## Author Contributions

**Lynn Govaert:** conceptualization (equal), data curation (equal), formal analysis (equal), investigation (equal), methodology (equal), supervision (supporting), visualization (equal), writing – original draft (equal), writing – review and editing (equal). **Jessica Colombo:** data curation (equal), formal analysis (equal), investigation (equal), writing – original draft (equal). **Florian Altermatt:** conceptualization (equal), funding acquisition (lead), investigation (equal), project administration (lead), resources (lead), supervision (lead), writing – review and editing (equal).

## Funding

This work was supported by Deutsche Forschungsgemeinschaft (511084840). Schweizerischer Nationalfonds zur Förderung der Wissenschaftlichen Forschung (310030_197410).

## Conflicts of Interest

The authors declare no conflicts of interest.

## Supporting information


**Table S1:** Detailed list of videos that were omitted from statistical analysis due to moving medium.
**Table S2:‐A** Statistical summary output from repeated‐measures linear mixed effect model using community biomass (log‐transformed summed individual bio‐areas) as a response variable and disturbance intensity and subsidy flow as fixed predicted variables, time as a repeated measure and replicate as a random effect. We report regression estimate ± standard error (S.E.), degrees of freedom (df), value of the t‐statistic and *p*‐value. *p*‐values below 0.05 are indicated in bold and represent significant effects. Statistical analysis is performed for the local low‐ and high‐resource ecosystems separately. The results using the patch 2 experimental units are reported in Table S2‐B.
**Table S2:‐B** Statistical summary output from repeated‐measures linear mixed effect model using community biomass (log‐transformed summed individual bio‐areas) as a response variable and disturbance intensity and subsidy flow as fixed predicted variables, time as a repeated measure and replicate as a random effect. We report regression estimate ± standard error (S.E.), degrees of freedom (df), value of the t‐statistic and *p*‐value. *p*‐values below 0.05 are indicated in bold and represent significant effects. Statistical analysis is performed for the local low‐ and high‐resource ecosystems separately.
**Table S3:‐A** Log‐response ratio of community biomass comparing each time point to the first time point for (a) isolated ecosystems, (b) ecosystems connected to a low‐resource ecosystem and (c) ecosystems connected to a high‐resource ecosystems. First value gives the log‐response ratio estimate and values between brackets indicate the 95% confidence interval. Values in bold reflect where the 95% confidence interval does not overlap with zero. The results using the patch 2 experimental units are reported in Table S3‐B.
**Table S3:‐B** Log‐response ratio of community biomass comparing each time point to the first time point using the patch 2 experimental units.
**Table S4:‐A** Log‐response ratio of community biomass at each time point comparing each experimental treatment combination to the control, undisturbed isolated local low‐resource ecosystem for (a) isolated ecosystems, (b) ecosystems connected to a low‐resource ecosystem and (c) ecosystems connected to a high‐resource ecosystems. First value gives the log‐response ratio estimate and values between brackets indicate the 95% confidence interval. Values in bold reflect where the 95% confidence interval does not overlap with zero. The results using the patch 2 experimental units are reported in Table S4‐B.
**Table S4:‐B** Log‐response ratio of community biomass at each time point comparing each experimental treatment combination to the control, undisturbed isolated local low‐resource ecosystem using the patch 2 experimental units.
**Table S5:‐A** Statistical summary output from repeated‐measures linear mixed effect model using density (log‐transformed) as a response variable and disturbance intensity and subsidy flow as fixed predicted variables, time as a repeated measure and replicate as a random effect. We report regression estimate ± standard error (S.E.), degrees of freedom (df), value of the *t*‐statistic and *p*‐value. *p*‐values below 0.05 are indicated in bold and represent significant effects. Statistical analysis is performed for the local low‐ and high‐resource ecosystems separately. The results using the patch 2 experimental units are reported in Table S5‐B.
**Table S5:‐B** Statistical summary output from repeated‐measures linear mixed effect model using density (log‐transformed) as a response variable and disturbance intensity and subsidy flow as fixed predicted variables, time as a repeated measure and replicate as a random effect. We report regression estimate ± standard error (S.E.), degrees of freedom (df), value of the *t*‐statistic and *p*‐value. *p*‐values below 0.05 are indicated in bold and represent significant effects. Statistical analysis is performed for the local low‐ and high‐resource ecosystems separately.
**Table S6:‐A** Log‐response ratio of log‐transformed density comparing each time point to the first time point for (a) isolated ecosystems, (b) ecosystems connected to a low‐resource ecosystem and (c) ecosystems connected to a high‐resource ecosystem. The first value gives the log‐response ratio estimate and values between brackets indicate the 95% confidence interval. Values in bold reflect where the 95% confidence interval does not overlap with zero. The results using the patch 2 experimental units are reported in Table S6‐B.
**Table S6:‐B** Log‐response ratio of log‐transformed density comparing each time point to the first time point for (a) isolated ecosystems, (b) ecosystems connected to a low‐resource ecosystem and (c) ecosystems connected to a high‐resource ecosystem. The first value gives the log‐response ratio estimate and values between brackets indicate the 95% confidence interval. Values in bold reflect where the 95% confidence interval does not overlap with zero.
**Table S7:‐A** Log‐response ratio of density (log‐transformed) at each time point comparing each experimental treatment combination to the control, undisturbed isolated local low‐resource ecosystem for (a) isolated ecosystems, (b) ecosystems connected to a low‐resource ecosystem and (c) ecosystems connected to a high‐resource ecosystem. The first value gives the log‐response ratio estimate and values between brackets indicate the 95% confidence interval. Values in bold reflect where the 95% confidence interval does not overlap with zero. The results using the patch 2 experimental units are reported in Table S7‐B.
**Table S7:‐B** Log‐response ratio of density (log‐transformed) at each time point comparing each experimental treatment combination to the control, undisturbed isolated local low‐resource ecosystem for (a) isolated ecosystems, (b) ecosystems connected to a low‐resource ecosystem and (c) ecosystems connected to a high‐resource ecosystem using the patch 2 experimental units. The first value gives the log‐response ratio estimate and values between brackets indicate the 95% confidence interval. Values in bold reflect where the 95% confidence interval does not overlap with zero.
**Table S8:‐A** Statistical summary table from PERMANOVA on Hellinger‐transformed size class abundances. Hellinger‐transformed size class abundances are used as response variable, disturbance intensity, subsidy flow and time are used as fixed variables and replicate as a random factor. In the table we report degrees of freedom (df), sum of squares (SS), mean sum of squares (MS), *F*‐statistic (*F*‐value), *R* ^2^ and *p*‐value. Significant effects (*p* < 0.005) are indicated in bold. Analysis is performed for the local low‐ and high‐resource ecosystems separately. The results using the patch 2 experimental units are reported in Table S5‐B.
**Table S8:‐B** Statistical summary table from PERMANOVA on Hellinger‐transformed size class abundances using the patch 2 experimental units. Hellinger‐transformed size class abundances are used as response variable, disturbance intensity, subsidy flow and time are used as fixed variables and replicate as a random factor. Analysis is performed for the local low‐ and high‐resource ecosystems separately due to non‐independence of some experimental treatments. In the table we report degrees of freedom (df), sum of squares (SS), mean sum of squares (MS), *F*‐statistic (*F*‐value), *R* ^2^ and *p*‐value. Significant effects (*p* < 0.005) are indicated in bold.
**Table S9:‐A** Statistical summary output from repeated‐measures linear mixed effect model using size class diversity as a response variable and disturbance intensity and subsidy flow as fixed predicted variables, time as a repeated measure and replicate as a random effect. We report regression estimate ± standard error (S.E.), degrees of freedom (df), value of the t‐statistic and *p*‐value. *p*‐values below 0.05 are indicated in bold and represent significant effects. Statistical analysis is performed for the local low‐ and high‐resource ecosystems separately, but including the isolated ecosystems as a control in both analyses. The results using the patch 2 experimental units are reported in Table S9‐B.
**Table S9:‐B** Statistical summary output from repeated‐measures linear mixed effect model using size class diversity as a response variable and disturbance intensity and subsidy flow as fixed predicted variables, time as a repeated measure and replicate as a random effect using the patch 2 experimental units. We report regression estimate ± standard error (S.E.), degrees of freedom (df), value of the *t*‐statistic and *p*‐value. *p*‐values below 0.05 are indicated in bold and represent significant effects. Statistical analysis is performed for the local low‐ and high‐resource ecosystems separately, but including the isolated ecosystems as a control in both analyses.
**Table S10:‐A** Statistical summary output from repeated‐measures linear mixed effect model using size class evenness as a response variable and disturbance intensity and subsidy flow as fixed predicted variables, time as a repeated measure and replicate as a random effect. We report regression estimate ± standard error (S.E.), degrees of freedom (df), value of the t‐statistic and *p*‐value. *p*‐values below 0.05 are indicated in bold and represent significant effects. Statistical analysis is performed for the local low‐ and high‐resource ecosystems separately, but including the isolated ecosystems as a control in both analyses. The results using the patch 2 experimental units are reported in Table S10‐B.
**Table S10:‐B** Statistical summary output from repeated‐measures linear mixed effect model using size class evenness as a response variable and disturbance intensity and subsidy flow as fixed predicted variables, time as a repeated measure and replicate as a random effect using the patch 2 experimental units. We report regression estimate ± standard error (S.E.), degrees of freedom (df), value of the *t*‐statistic and *p*‐value. *p*‐values below 0.05 are indicated in bold and represent significant effects. Statistical analysis is performed for the local low‐ and high‐resource ecosystems separately, but including the isolated ecosystems as a control in both analyses.
**Table S11:‐A** Log‐response ratio of size class diversity comparing each time point to the first time point for (a) isolated ecosystems, (b) ecosystems connected to a low‐resource ecosystem and (c) ecosystems connected to a high‐resource ecosystem. The first value gives the log‐response ratio estimate and values between brackets indicate the 95% confidence interval. Values in bold reflect where the 95% confidence interval does not overlap with zero. The results using the patch 2 experimental units are reported in Table S11‐B.
**Table S11:‐B** Log‐response ratio of size class diversity comparing each time point to the first time point for (a) isolated ecosystems, (b) ecosystems connected to a low‐resource ecosystem and (c) ecosystems connected to a high‐resource ecosystem using the patch 2 experimental units. The first value gives the log‐response ratio estimate and values between brackets indicate the 95% confidence interval. Values in bold reflect where the 95% confidence interval does not overlap with zero.
**Table S12:‐A** Log‐response ratio of size class evenness comparing each time point to the first time point for (a) isolated ecosystems, (b) ecosystems connected to a low‐resource ecosystem and (c) ecosystems connected to a high‐resource ecosystem. The first value gives the log‐response ratio estimate and values between brackets indicate the 95% confidence interval. Values in bold reflect where the 95% confidence interval does not overlap with zero. The results using the patch 2 experimental units are reported in Table S12‐B.
**Table S12:‐B** Log‐response ratio of size class evenness comparing each time point to the first time point for (a) isolated ecosystems, (b) ecosystems connected to a low‐resource ecosystem and (c) ecosystems connected to a high‐resource ecosystem using the patch 2 experimental units. The first value gives the log‐response ratio estimate and values between brackets indicate the 95% confidence interval. Values in bold reflect where the 95% confidence interval does not overlap with zero.
**Table S13:‐A** Statistical summary table from PERMANOVA on Hellinger‐transformed size class abundances at each time point. Hellinger‐transformed size class abundances are used as response variable, disturbance intensity and subsidy flow are used as fixed variables and replicate as a random factor. Analysis is performed for the local low‐ and high‐resource ecosystems separately due to non‐independence of some experimental treatments. In the table we report degrees of freedom (df), sum of squares (SS), mean sum of squares (MS), *F*‐statistic (*F*‐value), *R* ^2^ and *p*‐value. Significant effects (*p* < 0.005) are indicated in bold. The results using the patch 2 experimental units are reported in Table S13‐B.
**Table S13:‐B** Statistical summary table from PERMANOVA on Hellinger‐transformed size class abundances at each time point using the patch 2 experimental units. Hellinger‐transformed size class abundances are used as response variable, disturbance intensity and subsidy flow are used as fixed variables and replicate as a random factor. Analysis is performed for the local low‐ and high‐resource ecosystems separately due to non‐independence of some experimental treatments. In the table we report degrees of freedom (df), sum of squares (SS), mean sum of squares (MS), *F*‐statistic (*F*‐value), *R* ^2^ and *p*‐value. Significant effects (*p* < 0.005) are indicated in bold.
**Table S14:‐A** Log‐response ratio of size class diversity at each time point comparing each experimental treatment combination to the control, undisturbed isolated local low‐resource ecosystem for (a) isolated ecosystems, (b) ecosystems connected to a low‐resource ecosystem and (c) ecosystems connected to a high‐resource ecosystem. The first value gives the log‐response ratio estimate and values between brackets indicate the 95% confidence interval. Values in bold reflect where the 95% confidence interval does not overlap with zero. The results using the patch 2 experimental units are reported in Table S14‐B.
**Table S14:‐B** Log‐response ratio of size class diversity at each time point comparing each experimental treatment combination to the control, undisturbed isolated local low‐resource ecosystem for (a) isolated ecosystems, (b) ecosystems connected to a low‐resource ecosystem and (c) ecosystems connected to a high‐resource ecosystem using the patch 2 experimental units. The first value gives the log‐response ratio estimate and values between brackets indicate the 95% confidence interval. Values in bold reflect where the 95% confidence interval does not overlap with zero.
**Table S15:‐A** Log‐response ratio of size class evenness at each time point comparing each experimental treatment combination to the control, undisturbed isolated local low‐resource ecosystem for (a) isolated ecosystems, (b) ecosystems connected to a low‐resource ecosystem and (c) ecosystems connected to a high‐resource ecosystem. The first value gives the log‐response ratio estimate and values between brackets indicate the 95% confidence interval. Values in bold reflect where the 95% confidence interval does not overlap with zero. The results using the patch 2 experimental units are reported in Table S14‐B.
**Table S15:‐B** Log‐response ratio of size class evenness at each time point comparing each experimental treatment combination to the control, undisturbed isolated local low‐resource ecosystem for (a) isolated ecosystems, (b) ecosystems connected to a low‐resource ecosystem and (c) ecosystems connected to a high‐resource ecosystem using the patch 2 experimental units. The first value gives the log‐response ratio estimate and values between brackets indicate the 95% confidence interval. Values in bold reflect where the 95% confidence interval does not overlap with zero.

**Figure S1:** Log responses ratios of time series of log‐transformed densities comparing each time point to T0 for (a) the isolated meta‐ecosystems, (b) the low‐resource connected ecosystems and (c) the high‐resource connected ecosystems. Log response ratios of density comparing each treatment against the control (here: low resource isolated unconnected ecosystem) at each time point for (d) the isolated meta‐ecosystems, (e) the low‐resource connected ecosystems and (f) the high‐resource connected ecosystems. Blue symbols are low‐resource focal systems, while red symbols indicate high resource focal systems, both with varying levels of total perturbations (20%, 50%, 70%, 90%), respectively.
**Figure S2:** Log response ratios of time series of evenness comparing each time point to T0 for (a) the isolated ecosystems, (b) the low‐resource connected ecosystems and (c) the high‐resource connected ecosystems. Log response ratios of evenness comparing each treatment against the control (here taken as the low resource isolated ecosystem) at each time point for (d) the isolated ecosystems, (e) the low‐resource connected ecosystems and (f) the high‐resource connected ecosystems. Blue symbols are low resource focal systems, while red symbols indicate high resource focal systems, both with varying levels of total perturbations (20%, 50%, 70%, 90%), respectively.
**Figure S3:** Visualization of community size class compositional trajectories from the start (unfilled symbols) to the end (filled symbols) of the experiment. We used Non‐Metric Dimensional Scaling analysis on Hellinger‐transformed size class abundances. Blue (resp. red) colors represent local low‐resource (resp. high‐resource) ecosystems with circles representing isolated ecosystems, squares representing ecosystems connected to a low‐resource ecosystem and triangles representing ecosystems connected to a high‐resource ecosystem. Color intensity reflects different disturbance intensity levels.

## Data Availability

The data and R code are available on Zenodo: https://doi.org/10.5281/zenodo.17526198.
