# Supplementary material for: Local Resource Availability and Subsidy Flow Mediate the Effects of Disturbances in Meta‐Ecosystems
Source: Ecol Evol. 2026 Apr 27;16(4):e73516. doi: 10.1002/ece3.73516 (PMC13112083; doi:10.1002/ece3.73516)
Supplement: Supplementary file 1 — Table S1: Detailed list of videos that were omitted from statistical analysis due to moving medium. Table S2:‐A Statistical summary output from repeated‐measures linear mixed effect model using community biomass (log‐transformed summed individual bio‐areas) as a response variable and disturbance intensity and subsidy flow as fixed predicted variables, time as a repeated measure and replicate as a random effect. We report regression estimate ± standard error (S.E.), degrees of freedom (df), value of the t‐statistic and p‐value. p‐values below 0.05 are indicated in bold and represent significant effects. Statistical analysis is performed for the local low‐ and high‐resource ecosystems separately. The results using the patch 2 experimental units are reported in Table S2‐B. Table S2:‐B Statistical summary output from repeated‐measures linear mixed effect model using community biomass (log‐transformed summed individual bio‐areas) as a response variable and disturbance intensity and subsidy flow as fixed predicted variables, time as a repeated measure and replicate as a random effect. We report regression estimate ± standard error (S.E.), degrees of freedom (df), value of the t‐statistic and p‐value. p‐values below 0.05 are indicated in bold and represent significant effects. Statistical analysis is performed for the local low‐ and high‐resource ecosystems separately. Table S3:‐A Log‐response ratio of community biomass comparing each time point to the first time point for (a) isolated ecosystems, (b) ecosystems connected to a low‐resource ecosystem and (c) ecosystems connected to a high‐resource ecosystems. First value gives the log‐response ratio estimate and values between brackets indicate the 95% confidence interval. Values in bold reflect where the 95% confidence interval does not overlap with zero. The results using the patch 2 experimental units are reported in Table S3‐B. Table S3:‐B Log‐response ratio of community biomass comparing each time point to the first ti [file ECE3-16-e73516-s002.docx]

**Supplementary Information A: Supplementary Tables**

Note that the local low-resource and local high-resource ecosystems connected to a low- and high-resource ecosystem, respectively, reflect a two-patch meta-ecosystem that consists of two ecosystems that both have low or high local resources, respectively. This means that when we focus on a focal ecosystem there is the option to either use all pre-defined patch 1 experimental units or pre-defined patch 2 experimental units. Therefore, we repeat all statistical analyses with either patch 1 or patch 2 experimental units to see whether this choice affects the results observed.

**Table S1**: Detailed list of videos that were omitted from statistical analysis due to moving medium.

| Time point | Samples removed |
| --- | --- |
| T0 | 14, 18, 21, 87, 89, 105, 178 |
| T1 | 46, 55, 58, 71, 78, 88, 134, 138, 152, 165, 180, 192 |
| T2 | 105,132,133,137,165 |
| T3 | 45,67,115,122 |
| T4 | 69,117,142,152,153,157,178,191 |
| T5 | 24,35,88,112,117,136,189 |
| T6 | 11,23,29,32,45,69,89,100,104,116,122 |
| T7 | 92,97,132,147,162,176,189,192 |
| T8 | 22,85,129,161 |
| T9 | 50,66,84,99,127,187 |
| T10 | 61,121 |

**Table S2-A**: Statistical summary output from repeated-measures linear mixed effect model using community biomass (log-transformed summed individual bio-areas) as a response variable and disturbance intensity and subsidy flow as fixed predicted variables, time as a repeated measure and replicate as a random effect. We report regression estimate ± standard error (S.E.), degrees of freedom (df), value of the t-statistic and p-value. p-values below 0.05 are indicated in bold and represent significant effects. Statistical analysis is performed for the local low- and high-resource ecosystems separately. The results using the patch 2 experimental units are reported in Supplementary Table S2-B.

***(a) Local low-resource ecosystems*** (Shapiro-Wilk normality test: W = 0.96, p < 0.001)

|  | Estimate ± S.E. | df | t-value | p-value |
| --- | --- | --- | --- | --- |
| Low subsidy flow | -0.06 ± 0.10 | 746.12 | -0.65 | 0.514 |
| **High subsidy flow** | **0.37 ± 0.10** | **746.12** | **3.82** | **< 0.001** |
| **Disturbance 50** | **-1.01 ± 0.10** | **746.34** | **-10.36** | **< 0.001** |
| **Disturbance 70** | **-1.40 ± 0.10** | **746.16** | **-14.45** | **< 0.001** |
| **Disturbance 90** | **-2.57 ± 0.10** | **746.19** | **-26.34** | **< 0.001** |
| Low subsidy flow × Dist. 50 | 0.14 ± 0.14 | 746.19 | 0.99 | 0.324 |
| Low subsidy flow × Dist. 70 | 0.22 ± 0.14 | 746.17 | 1.57 | 0.117 |
| **Low subsidy flow × Dist. 90** | **0.32 ± 0.14** | **746.20** | **2.34** | **0.020** |
| High subsidy flow × Dist. 50 | 0.15 ± 0.14 | 746.37 | 1.06 | 0.288 |
| High subsidy flow × Dist. 70 | 0.18 ± 0.14 | 746.17 | 1.30 | 0.193 |
| High subsidy flow × Dist. 90 | 0.21 ± 0.14 | 746.16 | 1.52 | 0.130 |

***(b) Local high-resource ecosystems*** (Shapiro-Wilk normality test: W = 0.97, p < 0.001)

|  | Estimate ± S.E. | df | t-value | p-value |
| --- | --- | --- | --- | --- |
| Low subsidy flow | -0.10 ± 0.09 | 732.1 | -1.13 | 0.259 |
| High subsidy flow | 0.13 ± 0.09 | 732.0 | -1.43 | 0.153 |
| **Disturbance 50** | **-0.53 ± 0.09** | **732.0** | **-5.85** | **< 0.001** |
| **Disturbance 70** | **-0.34 ± 0.09** | **732.1** | **-3.71** | **< 0.001** |
| **Disturbance 90** | **-0.52 ± 0.09** | **732.1** | **-5.67** | **< 0.001** |
| **Low subsidy flow x Dist. 50** | **0.42 ± 0.13** | **732.1** | **3.20** | **0.001** |
| Low subsidy flow x Dist. 70 | 0.02 ± 0.13 | 732.1 | 0.15 | 0.882 |
| Low subsidy flow x Dist. 90 | 0.12 ± 0.13 | 732.1 | 0.01 | 0.993 |
| **High subsidy flow x Dist. 50** | **0.52 ± 0.13** | **732.0** | **4.00** | **< 0.001** |
| High subsidy flow x Dist. 70 | 0.22 ± 0.13 | 732.1 | 1.66 | 0.100 |
| High subsidy flow x Dist. 90 | 0.00 ± 0.13 | 732.1 | 0.00 | 0.999 |

**Table S2-B**: Statistical summary output from repeated-measures linear mixed effect model using community biomass (log-transformed summed individual bio-areas) as a response variable and disturbance intensity and subsidy flow as fixed predicted variables, time as a repeated measure and replicate as a random effect. We report regression estimate ± standard error (S.E.), degrees of freedom (df), value of the t-statistic and p-value. p-values below 0.05 are indicated in bold and represent significant effects. Statistical analysis is performed for the local low- and high-resource ecosystems separately.

***(a) Local low-resource ecosystems*** (Shapiro-Wilk normality test: W = 0.96, p < 0.001)

|  | Estimate ± S.E. | df | t-value | p-value |
| --- | --- | --- | --- | --- |
| Low resource metaflow | -0.17 ± 0.10 | 742.02 | -1.69 | 0.091 |
| **High resource metaflow** | **0.37 ± 0.10** | **742.06** | **3.70** | **< 0.001** |
| **Disturbance 50** | **-1.01 ± 0.10** | **742.18** | **-14.00** | **< 0.001** |
| **Disturbance 70** | **-1.40 ± 0.10** | **742.08** | **-14.45** | **< 0.001** |
| **Disturbance 90** | **-2.57 ± 0.10** | **742.11** | **-25.49** | **< 0.001** |
| Low subsidy flow x Dist. 50 | 0.15 ± 0.14 | 742.09 | 1.09 | 0.278 |
| Low subsidy flow x Dist. 70 | 0.23 ± 0.14 | 742.18 | 1.63 | 0.103 |
| **Low subsidy flow x Dist. 90** | **0.57 ± 0.14** | **742.10** | **4.04** | **< 0.001** |
| High subsidy flow x Dist. 50 | 0.15 ± 0.14 | 742.20 | 1.04 | 0.299 |
| High subsidy flow x Dist. 70 | 0.18 ± 0.14 | 742.09 | 1.27 | 0.206 |
| High subsidy flow x Dist. 90 | 0.21 ± 0.14 | 742.10 | 1.48 | 0.139 |

***(b) Local high-resource ecosystems*** (Shapiro-Wilk normality test: W = 0.97, p < 0.001)

|  | Estimate ± S.E. | df | t-value | p-value |
| --- | --- | --- | --- | --- |
| Low resource metaflow | -0.11 ± 0.09 | 727.12 | -1.16 | 0.245 |
| **High resource metaflow** | **-0.23 ± 0.09** | **727.26** | **-2.61** | **0.009** |
| **Disturbance 50** | **-0.53 ± 0.09** | **727.04** | **-5.90** | **< 0.001** |
| **Disturbance 70** | **-0.34 ± 0.09** | **727.21** | **-3.71** | **< 0.001** |
| **Disturbance 90** | **-0.52 ± 0.09** | **727.15** | **-5.69** | **< 0.001** |
| **Low subsidy flow x Dist. 50** | **0.42 ± 0.13** | **727.12** | **3.27** | **0.001** |
| Low subsidy flow x Dist. 70 | 0.02 ± 0.13 | 727.20 | 0.13 | 0.894 |
| Low subsidy flow x Dist. 90 | 0.00 ± 0.13 | 727.19 | 0.03 | 0.974 |
| **High subsidy flow x Dist. 50** | **0.36 ± 0.13** | **727.32** | **2.79** | **0.005** |
| High subsidy flow x Dist. 70 | 0.14 ± 0.13 | 727.19 | 1.05 | 0.296 |
| High subsidy flow x Dist. 90 | 0.09 ± 0.13 | 727.19 | 0.71 | 0.476 |

**Table S3-A**: Log-response ratio of community biomass comparing each time point to the first time point for (a) isolated ecosystems, (b) ecosystems connected to a low-resource ecosystem and (c) ecosystems connected to a high-resource ecosystems. First value gives the log-response ratio estimate and values between brackets indicate the 95% confidence interval. Values in bold reflect where the 95% confidence interval does not overlap with zero. The results using the patch 2 experimental units are reported in Supplementary Table S3-B.

***(a) Isolated ecosystems***

*Local low resource ecosystem*

| 0% | 50% | 70% | 90% |
| --- | --- | --- | --- |
| **0.0179(0.0065,0.0292)** | **-0.0422(-0.0557,-0.0286)** | **-0.0583(-0.0703,-0.0463)** | **-0.133(-0.1541,-0.1119)** |
| **0.0314(0.0214,0.0414)** | **-0.0422(-0.0647,-0.0198)** | **-0.0742(-0.0972,-0.0512)** | **-0.1841(-0.2248,-0.1433)** |
| **0.0354(0.0237,0.0472)** | **-0.0425(-0.0594,-0.0255)** | **-0.0887(-0.109,-0.0684)** | **-0.1904(-0.2364,-0.1444)** |
| **0.0344(0.0154,0.0535)** | **-0.0469(-0.0677,-0.0262)** | **-0.1066(-0.1276,-0.0857)** | **-0.1919(-0.231,-0.1528)** |
| **0.0309(0.0179,0.0438)** | **-0.0612(-0.0793,-0.0432)** | **-0.1208(-0.1358,-0.1057)** | **-0.2318(-0.2649,-0.1986)** |
| **0.0196(0.0076,0.0315)** | **-0.0702(-0.0947,-0.0458)** | **-0.0991(-0.1219,-0.0763)** | **-0.2424(-0.284,-0.2007)** |
| 0.0122(-0.009,0.0333) | **-0.1013(-0.1324,-0.0703)** | **-0.1403(-0.1763,-0.1043)** | **-0.2237(-0.2536,-0.1937)** |
| -0.0054(-0.0419,0.0312) | **-0.0879(-0.1154,-0.0603)** | **-0.1434(-0.1721,-0.1148)** | **-0.3752(-0.4928,-0.2576)** |
| -0.0062(-0.0394,0.027) | **-0.1425(-0.1737,-0.1113)** | **-0.1621(-0.1805,-0.1437)** | **-0.3102(-0.3932,-0.2272)** |
| -6e-04(-0.0333,0.0322) | **-0.1124(-0.1514,-0.0734)** | **-0.1425(-0.1613,-0.1236)** | **-0.3074(-0.3424,-0.2724)** |

*Local high resource ecosystem*

| 0% | 50% | 70% | 90% |
| --- | --- | --- | --- |
| **0.0287(0.014,0.0435)** | -0.0029(-0.022,0.0161) | -0.0033(-0.0314,0.0247) | **-0.0382(-0.0723,-0.0041)** |
| **0.0288(0.0069,0.0506)** | **-0.0173(-0.0345,-1e-04)** | -0.0066(-0.0242,0.011) | **-0.0511(-0.0859,-0.0163)** |
| **0.0416(0.0167,0.0665)** | -0.0152(-0.0441,0.0136) | 0.0022(-0.0283,0.0328) | **-0.0388(-0.0752,-0.0024)** |
| **0.05(0.0239,0.0761)** | -0.0032(-0.0237,0.0173) | 0.0103(-0.034,0.0545) | -0.0164(-0.0518,0.0191) |
| **0.0589(0.0327,0.0852)** | 0.0036(-0.014,0.0211) | 0.0156(-0.0268,0.0581) | 0.008(-0.0519,0.0679) |
| **0.0675(0.0432,0.0919)** | 0.0065(-0.0203,0.0334) | 0.0226(-0.0234,0.0686) | 0.0215(-0.0189,0.062) |
| **0.0693(0.0398,0.0988)** | 0.0196(-0.0091,0.0483) | 0.0266(-0.0112,0.0643) | **0.0405(0.0024,0.0787)** |
| **0.0501(0.0141,0.0861)** | 0.0182(-0.0114,0.0479) | 0.0346(-0.0211,0.0903) | 0.0374(-0.0097,0.0846) |
| **0.0548(0.0224,0.0872)** | 0.0139(-0.0141,0.042) | **0.0374(0,0.0749)** | 0.0218(-0.019,0.0626) |
| 0.0249(-0.0716,0.1215) | -0.0092(-0.0488,0.0304) | 0.0329(-0.0177,0.0835) | 0.0241(-0.0185,0.0668) |

***(b) Low-resource connected ecosystems***

*Local low resource ecosystem*

| 0% | 50% | 70% | 90% |
| --- | --- | --- | --- |
| 0.0012(-0.0121,0.0144) | **-0.0199(-0.0347,-0.0051)** | **-0.0581(-0.0816,-0.0346)** | **-0.1269(-0.1491,-0.1047)** |
| 3e-04(-0.0144,0.015) | **-0.0355(-0.0504,-0.0207)** | **-0.0733(-0.0886,-0.0581)** | **-0.1429(-0.1794,-0.1063)** |
| 8e-04(-0.0182,0.0198) | **-0.0529(-0.0752,-0.0307)** | **-0.0884(-0.1012,-0.0757)** | **-0.1695(-0.1959,-0.1432)** |
| 0.0081(-0.014,0.0302) | **-0.0571(-0.0814,-0.0329)** | **-0.0889(-0.1054,-0.0724)** | **-0.197(-0.2251,-0.169)** |
| 0.0099(-0.0103,0.0302) | **-0.0688(-0.0904,-0.0472)** | **-0.115(-0.1366,-0.0934)** | **-0.2218(-0.2598,-0.1838)** |
| 0.0116(-0.0068,0.03) | **-0.0726(-0.0893,-0.056)** | **-0.1107(-0.1282,-0.0932)** | **-0.239(-0.289,-0.1891)** |
| 0.0042(-0.0109,0.0194) | **-0.0896(-0.1097,-0.0695)** | **-0.1021(-0.131,-0.0733)** | **-0.2249(-0.2799,-0.1699)** |
| 0.0046(-0.0118,0.021) | **-0.1163(-0.1372,-0.0954)** | **-0.1181(-0.1438,-0.0923)** | **-0.2659(-0.3129,-0.219)** |
| -0.002(-0.0154,0.0113) | **-0.1283(-0.1446,-0.112)** | **-0.1511(-0.1697,-0.1325)** | **-0.3364(-0.3981,-0.2747)** |
| -0.0108(-0.0232,0.0016) | **-0.1432(-0.158,-0.1285)** | **-0.1801(-0.2034,-0.1568)** | **-0.2557(-0.3162,-0.1952)** |

*Local high resource ecosystem*

| 0% | 50% | 70% | 90% |
| --- | --- | --- | --- |
| 0.004(-0.0208,0.0288) | -0.0088(-0.0252,0.0076) | **-0.0437(-0.0645,-0.0229)** | -0.0517(-0.1099,0.0064) |
| 0.0029(-0.0271,0.0329) | **-0.0297(-0.0557,-0.0036)** | **-0.0717(-0.0877,-0.0557)** | **-0.0958(-0.1394,-0.0522)** |
| -0.0207(-0.045,0.0036) | **-0.0278(-0.0522,-0.0034)** | **-0.0525(-0.076,-0.0291)** | **-0.1081(-0.1531,-0.063)** |
| 0.0012(-0.0389,0.0412) | -0.0172(-0.0469,0.0126) | **-0.0707(-0.0918,-0.0496)** | **-0.0846(-0.1286,-0.0405)** |
| -0.0059(-0.0397,0.028) | -0.0126(-0.049,0.0238) | **-0.0438(-0.0738,-0.0138)** | -0.0431(-0.0978,0.0115) |
| 0(-0.0414,0.0415) | -0.0187(-0.0588,0.0214) | -0.0114(-0.0344,0.0116) | -0.0171(-0.0666,0.0325) |
| 0.0044(-0.0285,0.0372) | -0.003(-0.0403,0.0343) | -0.0064(-0.0495,0.0368) | -0.0124(-0.0764,0.0516) |
| -0.0019(-0.0297,0.0259) | -0.0036(-0.0479,0.0406) | 0.007(-0.0179,0.032) | -0.0078(-0.0472,0.0316) |
| -0.0193(-0.0648,0.0261) | 5e-04(-0.0514,0.0524) | -0.003(-0.0378,0.0318) | -0.0187(-0.0581,0.0207) |
| 0.0036(-0.0297,0.0368) | -0.0076(-0.0603,0.0451) | -0.0065(-0.0455,0.0324) | -0.0239(-0.0665,0.0187) |

***(c) High-resource connected ecosystems***

*Local low resource ecosystem*

| 0% | 50% | 70% | 90% |
| --- | --- | --- | --- |
| 0.0023(-0.0166,0.0213) | -0.016(-0.0342,0.0021) | **-0.0395(-0.0588,-0.0202)** | **-0.1088(-0.1226,-0.0949)** |
| 0.0068(-0.0127,0.0263) | **-0.0182(-0.0356,-8e-04)** | **-0.0838(-0.0971,-0.0705)** | **-0.1392(-0.1818,-0.0967)** |
| 0.0135(-0.0024,0.0294) | **-0.0316(-0.044,-0.0193)** | **-0.0602(-0.0868,-0.0335)** | **-0.1718(-0.2082,-0.1353)** |
| **0.0313(0.0133,0.0492)** | **-0.0306(-0.0591,-0.0021)** | **-0.0604(-0.0754,-0.0454)** | **-0.1598(-0.2088,-0.1109)** |
| **0.0385(0.0291,0.0478)** | -0.0179(-0.0498,0.014) | **-0.0732(-0.0947,-0.0518)** | **-0.1785(-0.2702,-0.0868)** |
| **0.0581(0.0379,0.0782)** | **-0.0281(-0.0483,-0.0078)** | **-0.0523(-0.0717,-0.0328)** | **-0.2018(-0.2697,-0.1339)** |
| **0.0593(0.037,0.0816)** | **-0.033(-0.0548,-0.0112)** | **-0.0512(-0.0767,-0.0256)** | **-0.1643(-0.1946,-0.134)** |
| **0.0557(0.0373,0.0742)** | **-0.0324(-0.0615,-0.0034)** | **-0.0694(-0.0858,-0.0529)** | **-0.2072(-0.2806,-0.1338)** |
| **0.0648(0.0439,0.0856)** | **-0.0437(-0.0651,-0.0224)** | **-0.0807(-0.1043,-0.0571)** | **-0.2867(-0.483,-0.0904)** |
| **0.0881(0.0698,0.1065)** | **-0.0652(-0.104,-0.0264)** | **-0.0919(-0.118,-0.0658)** | **-0.2251(-0.3351,-0.115)** |

*Local high resource ecosystem*

| 0% | 50% | 70% | 90% |
| --- | --- | --- | --- |
| 0.0019(-0.0177,0.0216) | -0.0039(-0.023,0.0152) | -0.0141(-0.0425,0.0144) | **-0.0797(-0.119,-0.0404)** |
| -0.0057(-0.028,0.0166) | -4e-04(-0.0149,0.0142) | **-0.0356(-0.0589,-0.0124)** | **-0.1096(-0.146,-0.0733)** |
| -0.0034(-0.0211,0.0142) | -0.016(-0.0422,0.0102) | -0.0396(-0.0835,0.0044) | **-0.0859(-0.1246,-0.0471)** |
| -0.006(-0.0304,0.0184) | -0.0075(-0.0315,0.0166) | -0.012(-0.0761,0.0521) | **-0.0741(-0.1132,-0.035)** |
| 0.0088(-0.0131,0.0307) | 0.002(-0.0243,0.0282) | -0.0141(-0.0734,0.0452) | -0.0154(-0.0448,0.014) |
| 0.0183(-0.0093,0.0459) | -0.011(-0.0367,0.0147) | 0.0105(-0.0458,0.0668) | -0.009(-0.0421,0.0241) |
| 0.031(-0.0041,0.0662) | **0.0295(0.0103,0.0487)** | 0.0034(-0.0487,0.0555) | 0.0052(-0.0165,0.0268) |
| 0.0162(-0.0204,0.0527) | **0.0295(0.0096,0.0494)** | 0.0271(-0.0228,0.0771) | 0.0069(-0.0214,0.0352) |
| 0.0261(-0.0023,0.0545) | **0.0375(0.0177,0.0573)** | 0.0193(-0.024,0.0626) | -0.0118(-0.0342,0.0106) |
| 9e-04(-0.0602,0.062) | 0.0157(-0.0205,0.0519) | 0.014(-0.0334,0.0614) | 6e-04(-0.0361,0.0372) |

**Table S3-B**: Log-response ratio of community biomass comparing each time point to the first time point using the patch 2 experimental units.

***(a) Low-resource connected ecosystems***

*Local low resource ecosystem*

| -0.0097(-0.0231,0.0037) | **-0.0255(-0.0473,-0.0037)** | **-0.0747(-0.1072,-0.0421)** | **-0.1045(-0.1363,-0.0727)** |
| --- | --- | --- | --- |
| -7e-04(-0.0155,0.0142) | **-0.0315(-0.0475,-0.0155)** | **-0.0673(-0.0796,-0.055)** | **-0.1469(-0.1838,-0.11)** |
| -0.0068(-0.0287,0.0152) | **-0.0677(-0.0838,-0.0516)** | **-0.0856(-0.1073,-0.0639)** | **-0.1705(-0.228,-0.113)** |
| 0.0074(-0.0132,0.028) | **-0.0653(-0.0913,-0.0393)** | **-0.1113(-0.1259,-0.0967)** | **-0.2037(-0.2352,-0.1722)** |
| 0.0113(-0.0056,0.0283) | **-0.0725(-0.0993,-0.0456)** | **-0.1317(-0.1472,-0.1162)** | **-0.2735(-0.3016,-0.2453)** |
| 0.0028(-0.0169,0.0225) | **-0.0787(-0.1004,-0.0571)** | **-0.1041(-0.1169,-0.0914)** | **-0.1966(-0.2472,-0.146)** |
| 1e-04(-0.0307,0.0309) | **-0.1192(-0.1443,-0.0941)** | **-0.1088(-0.1241,-0.0935)** | **-0.2384(-0.2787,-0.198)** |
| -0.0117(-0.0428,0.0195) | **-0.1253(-0.1512,-0.0995)** | **-0.1376(-0.1675,-0.1077)** | **-0.2041(-0.2467,-0.1614)** |
| -0.0061(-0.0279,0.0157) | **-0.1253(-0.1432,-0.1074)** | **-0.122(-0.1561,-0.0879)** | **-0.2692(-0.3383,-0.2001)** |
| -0.0214(-0.0834,0.0406) | **-0.1069(-0.1573,-0.0565)** | **-0.1654(-0.2157,-0.1152)** | **-0.1849(-0.2884,-0.0814)** |

***(b) High-resource connected ecosystems***

*Local high resource ecosystem*

| -0.008(-0.0229,0.0068) | -0.0248(-0.0533,0.0037) | -0.0309(-0.0705,0.0087) | **-0.0816(-0.1262,-0.0371)** |
| --- | --- | --- | --- |
| -0.003(-0.0311,0.0251) | -0.0263(-0.0663,0.0137) | **-0.0469(-0.0673,-0.0265)** | **-0.1031(-0.1414,-0.0648)** |
| **-0.0205(-0.0392,-0.0018)** | **-0.0407(-0.0618,-0.0196)** | **-0.0456(-0.0767,-0.0145)** | **-0.0903(-0.1443,-0.0362)** |
| **-0.0309(-0.0541,-0.0076)** | **-0.0321(-0.0636,-6e-04)** | **-0.0361(-0.0578,-0.0143)** | **-0.0534(-0.0928,-0.0139)** |
| 8e-04(-0.0484,0.05) | -0.0425(-0.1112,0.0261) | **-0.0315(-0.0601,-0.0028)** | -0.0144(-0.0478,0.019) |
| -0.0037(-0.0622,0.0547) | -0.0171(-0.0488,0.0145) | -0.0106(-0.037,0.0157) | -0.0229(-0.0568,0.0109) |
| 0.015(-0.0249,0.0549) | -0.0135(-0.0406,0.0136) | 0.0109(-0.0238,0.0457) | 0.0176(-0.0222,0.0574) |
| 0.0104(-0.0328,0.0536) | 0.0079(-0.0298,0.0456) | 0.008(-0.0331,0.049) | -0.0066(-0.0422,0.0289) |
| 0.0168(-0.0264,0.0599) | 0.0137(-0.0211,0.0485) | 0.0089(-0.0224,0.0401) | -0.0135(-0.0506,0.0235) |
| 0.0173(-0.0165,0.0511) | 0.0183(-0.0057,0.0424) | 0.0076(-0.0188,0.034) | -0.012(-0.0355,0.0114) |

**Table S4-A**: Log-response ratio of community biomass at each time point comparing each experimental treatment combination to the control, undisturbed isolated local low-resource ecosystem for (a) isolated ecosystems, (b) ecosystems connected to a low-resource ecosystem and (c) ecosystems connected to a high-resource ecosystems. First value gives the log-response ratio estimate and values between brackets indicate the 95% confidence interval. Values in bold reflect where the 95% confidence interval does not overlap with zero. The results using the patch 2 experimental units are reported in Supplementary Table S4-B.

***(a) Isolated ecosystems***

*Local low resource ecosystem*

| 0% | 50% | 70% | 90% |
| --- | --- | --- | --- |
|  | **-0.06(-0.0746,-0.0454)** | **-0.0762(-0.0894,-0.063)** | **-0.1508(-0.1726,-0.129)** |
|  | **-0.0736(-0.0961,-0.0511)** | **-0.1056(-0.1286,-0.0826)** | **-0.2154(-0.2562,-0.1747)** |
|  | **-0.0779(-0.0959,-0.0598)** | **-0.1242(-0.1454,-0.1029)** | **-0.2258(-0.2723,-0.1794)** |
|  | **-0.0814(-0.1077,-0.055)** | **-0.141(-0.1676,-0.1145)** | **-0.2263(-0.2687,-0.184)** |
|  | **-0.0921(-0.112,-0.0723)** | **-0.1517(-0.1688,-0.1345)** | **-0.2627(-0.2968,-0.2285)** |
|  | **-0.0898(-0.1151,-0.0644)** | **-0.1186(-0.1424,-0.0949)** | **-0.2619(-0.3041,-0.2197)** |
|  | **-0.1135(-0.1498,-0.0773)** | **-0.1525(-0.1931,-0.1119)** | **-0.2359(-0.2712,-0.2006)** |
|  | **-0.0825(-0.1272,-0.0379)** | **-0.1381(-0.1834,-0.0927)** | **-0.3698(-0.4926,-0.2471)** |
|  | **-0.1363(-0.1808,-0.0918)** | **-0.1559(-0.1925,-0.1193)** | **-0.304(-0.3928,-0.2152)** |
|  | **-0.1119(-0.1618,-0.0619)** | **-0.1419(-0.1784,-0.1055)** | **-0.3068(-0.3537,-0.26)** |

*Local high resource ecosystem*

| 0% | 50% | 70% | 90% |
| --- | --- | --- | --- |
| 0.0109(-0.0046,0.0264) | **-0.0208(-0.0404,-0.0012)** | **-0.0212(-0.0496,0.0072)** | **-0.056(-0.0905,-0.0216)** |
| -0.0026(-0.0244,0.0191) | **-0.0487(-0.0658,-0.0317)** | **-0.038(-0.0554,-0.0205)** | **-0.0825(-0.1172,-0.0477)** |
| 0.0062(-0.0194,0.0318) | **-0.0507(-0.0801,-0.0212)** | **-0.0332(-0.0643,-0.0021)** | **-0.0743(-0.1111,-0.0374)** |
| 0.0155(-0.0151,0.0462) | **-0.0376(-0.0636,-0.0116)** | -0.0241(-0.0712,0.0229) | **-0.0508(-0.0897,-0.0119)** |
| **0.0281(6e-04,0.0555)** | **-0.0273(-0.0466,-0.0081)** | -0.0153(-0.0584,0.0279) | -0.0229(-0.0833,0.0375) |
| **0.048(0.0229,0.0731)** | -0.013(-0.0406,0.0145) | 0.003(-0.0434,0.0494) | 0.002(-0.039,0.0429) |
| **0.0571(0.0223,0.092)** | 0.0074(-0.0267,0.0416) | 0.0144(-0.0276,0.0564) | 0.0283(-0.014,0.0707) |
| **0.0554(0.0052,0.1057)** | 0.0236(-0.0223,0.0695) | 0.04(-0.0258,0.1058) | 0.0428(-0.016,0.1015) |
| **0.061(0.0157,0.1062)** | 0.0201(-0.0221,0.0624) | 0.0436(-0.0054,0.0926) | 0.028(-0.0236,0.0796) |
| 0.0255(-0.0759,0.1269) | -0.0086(-0.059,0.0417) | 0.0334(-0.0259,0.0928) | 0.0247(-0.0281,0.0775) |

***(b) Low-resource connected ecosystems***

*Local low resource ecosystem*

| 0% | 50% | 70% | 90% |
| --- | --- | --- | --- |
| -0.0077(-0.0212,0.0059) | **-0.0288(-0.0438,-0.0137)** | **-0.0669(-0.0906,-0.0432)** | **-0.1357(-0.1581,-0.1133)** |
| **-0.0221(-0.0361,-0.0081)** | **-0.0579(-0.072,-0.0437)** | **-0.0957(-0.1102,-0.0811)** | **-0.1652(-0.2014,-0.129)** |
| **-0.0256(-0.0451,-0.0062)** | **-0.0793(-0.102,-0.0567)** | **-0.1148(-0.1283,-0.1014)** | **-0.1959(-0.2226,-0.1692)** |
| -0.0173(-0.0444,0.0097) | **-0.0825(-0.1113,-0.0537)** | **-0.1143(-0.137,-0.0916)** | **-0.2224(-0.2545,-0.1903)** |
| -0.0119(-0.0333,0.0095) | **-0.0906(-0.1133,-0.0679)** | **-0.1368(-0.1595,-0.1141)** | **-0.2436(-0.2822,-0.205)** |
| 0.0011(-0.0179,0.0201) | **-0.0832(-0.1005,-0.0659)** | **-0.1212(-0.1393,-0.1031)** | **-0.2496(-0.2997,-0.1994)** |
| 0.0011(-0.0225,0.0247) | **-0.0927(-0.1198,-0.0657)** | **-0.1053(-0.1394,-0.0712)** | **-0.228(-0.2859,-0.1701)** |
| 0.019(-0.0195,0.0575) | **-0.1019(-0.1425,-0.0613)** | **-0.1037(-0.147,-0.0604)** | **-0.2516(-0.31,-0.1931)** |
| 0.0132(-0.0209,0.0473) | **-0.1131(-0.1484,-0.0778)** | **-0.1359(-0.1723,-0.0994)** | **-0.3212(-0.3904,-0.252)** |
| -0.0012(-0.0345,0.032) | **-0.1337(-0.1678,-0.0995)** | **-0.1705(-0.2091,-0.1319)** | **-0.2462(-0.3141,-0.1783)** |

*Local high resource ecosystem*

| 0% | 50% | 70% | 90% |
| --- | --- | --- | --- |
| **0.0256(0.0011,0.0501)** | 0.0128(-0.0031,0.0288) | **-0.0221(-0.0425,-0.0016)** | **-0.0301(-0.0882,0.028)** |
| 0.011(-0.0183,0.0403) | -0.0215(-0.0468,0.0037) | **-0.0636(-0.0783,-0.049)** | **-0.0877(-0.1308,-0.0445)** |
| -0.0166(-0.0409,0.0076) | -0.0238(-0.0481,6e-04) | **-0.0485(-0.0718,-0.0251)** | **-0.104(-0.149,-0.059)** |
| 0.0063(-0.0365,0.049) | -0.0121(-0.0454,0.0212) | **-0.0656(-0.0914,-0.0398)** | **-0.0795(-0.126,-0.033)** |
| 0.0028(-0.0315,0.037) | -0.004(-0.0408,0.0327) | **-0.0352(-0.0656,-0.0047)** | -0.0345(-0.0894,0.0204) |
| 0.02(-0.0215,0.0614) | 0.0012(-0.0389,0.0414) | 0.0085(-0.0145,0.0315) | 0.0029(-0.0467,0.0524) |
| 0.0317(-0.0055,0.0689) | 0.0243(-0.0169,0.0655) | 0.0209(-0.0256,0.0675) | 0.0149(-0.0514,0.0812) |
| 0.043(-0.0014,0.0873) | 0.0412(-0.0149,0.0973) | 0.0519(0.0093,0.0945) | 0.037(-0.0154,0.0894) |
| 0.0263(-0.0287,0.0814) | 0.0462(-0.0143,0.1066) | 0.0427(-0.004,0.0893) | 0.027(-0.0231,0.0771) |
| 0.0436(-0.0015,0.0887) | 0.0325(-0.0284,0.0934) | 0.0335(-0.0159,0.083) | 0.0162(-0.0362,0.0685) |

***(c) High-resource connected ecosystems***

*Local low resource ecosystem*

| 0% | 50% | 70% | 90% |
| --- | --- | --- | --- |
| -0.0098(-0.0293,0.0097) | **-0.0282(-0.0469,-0.0095)** | **-0.0517(-0.0715,-0.0318)** | **-0.1209(-0.1355,-0.1063)** |
| -0.0189(-0.0382,4e-04) | **-0.0439(-0.0611,-0.0266)** | **-0.1095(-0.1225,-0.0964)** | **-0.1649(-0.2074,-0.1225)** |
| -0.0162(-0.0331,6e-04) | **-0.0613(-0.0749,-0.0478)** | **-0.0899(-0.1171,-0.0626)** | **-0.2015(-0.2384,-0.1646)** |
| 0.0026(-0.0215,0.0267) | **-0.0593(-0.092,-0.0266)** | **-0.0891(-0.111,-0.0671)** | **-0.1886(-0.2401,-0.137)** |
| **0.0133(0.0011,0.0255)** | **-0.0431(-0.0759,-0.0102)** | **-0.0984(-0.1212,-0.0756)** | **-0.2036(-0.2957,-0.1116)** |
| **0.0442(0.0232,0.0652)** | **-0.0419(-0.063,-0.0208)** | **-0.0661(-0.0864,-0.0458)** | **-0.2157(-0.2838,-0.1475)** |
| **0.0528(0.0238,0.0818)** | **-0.0395(-0.0681,-0.0109)** | **-0.0576(-0.0892,-0.0261)** | **-0.1708(-0.2063,-0.1353)** |
| **0.0668(0.0272,0.1064)** | -0.0214(-0.0669,0.0242) | **-0.0583(-0.097,-0.0196)** | **-0.1962(-0.2775,-0.1148)** |
| **0.0767(0.0388,0.1145)** | -0.0318(-0.07,0.0063) | **-0.0688(-0.1082,-0.0294)** | **-0.2748(-0.4737,-0.076)** |
| **0.0944(0.0583,0.1305)** | **-0.0589(-0.1086,-0.0092)** | **-0.0856(-0.1262,-0.0451)** | **-0.2188(-0.3331,-0.1045)** |

*Local high resource ecosystem*

| 0% | 50% | 70% | 90% |
| --- | --- | --- | --- |
| 0.0117(-0.0073,0.0307) | 0.0059(-0.0125,0.0243) | -0.0043(-0.0323,0.0237) | **-0.0699(-0.1089,-0.0309)** |
| -0.0095(-0.0305,0.0116) | -0.0041(-0.0167,0.0084) | **-0.0394(-0.0614,-0.0174)** | **-0.1134(-0.149,-0.0778)** |
| -0.0112(-0.0285,0.006) | -0.0238(-0.0497,0.0021) | **-0.0474(-0.0912,-0.0036)** | **-0.0937(-0.1322,-0.0551)** |
| -0.0128(-0.0412,0.0156) | -0.0143(-0.0423,0.0138) | -0.0188(-0.0845,0.047) | **-0.0809(-0.1225,-0.0392)** |
| 0.0056(-0.0167,0.0278) | -0.0013(-0.0278,0.0252) | -0.0174(-0.0768,0.042) | -0.0186(-0.0482,0.0109) |
| 0.0264(-0.0011,0.0538) | -0.0029(-0.0284,0.0226) | 0.0186(-0.0376,0.0748) | -9e-04(-0.0339,0.032) |
| 0.0465(0.0073,0.0856) | **0.045(0.0193,0.0707)** | 0.0188(-0.036,0.0737) | 0.0206(-0.007,0.0482) |
| 0.0491(-0.001,0.0993) | **0.0625(0.0228,0.1022)** | 0.0601(-5e-04,0.1207) | 0.0399(-0.0046,0.0844) |
| 0.0599(0.018,0.1018) | **0.0713(0.0347,0.1079)** | **0.0531(0,0.1062)** | 0.022(-0.0161,0.0601) |
| 0.0291(-0.0391,0.0973) | 0.0439(-0.0033,0.091) | 0.0422(-0.014,0.0984) | 0.0288(-0.0188,0.0763) |

**Table S4-B**: Log-response ratio of community biomass at each time point comparing each experimental treatment combination to the control, undisturbed isolated local low-resource ecosystem using the patch 2 experimental units.

***(a) Low-resource connected ecosystems***

*Local low resource ecosystem*

| 0% | 50% | 70% | 90% |
| --- | --- | --- | --- |
| **-0.0226(-0.0361,-0.009)** | **-0.0384(-0.0603,-0.0165)** | **-0.0876(-0.1202,-0.0549)** | **-0.1174(-0.1492,-0.0855)** |
| **-0.0271(-0.0411,-0.0131)** | **-0.0579(-0.0732,-0.0427)** | **-0.0937(-0.105,-0.0824)** | **-0.1733(-0.2099,-0.1367)** |
| **-0.0373(-0.0596,-0.015)** | **-0.0982(-0.1147,-0.0816)** | **-0.1161(-0.1381,-0.094)** | **-0.201(-0.2586,-0.1434)** |
| -0.022(-0.0478,0.0037) | **-0.0948(-0.125,-0.0645)** | **-0.1407(-0.1621,-0.1194)** | **-0.2332(-0.2682,-0.1981)** |
| -0.0146(-0.0328,0.0036) | **-0.0984(-0.126,-0.0707)** | **-0.1576(-0.1745,-0.1407)** | **-0.2994(-0.3283,-0.2705)** |
| -0.0118(-0.032,0.0083) | **-0.0934(-0.1154,-0.0713)** | **-0.1187(-0.1322,-0.1052)** | **-0.2112(-0.262,-0.1604)** |
| -0.0071(-0.0428,0.0286) | **-0.1265(-0.1574,-0.0956)** | **-0.116(-0.1396,-0.0924)** | **-0.2456(-0.2898,-0.2014)** |
| -0.0013(-0.0481,0.0454) | **-0.115(-0.1584,-0.0717)** | **-0.1273(-0.1731,-0.0814)** | **-0.1937(-0.2488,-0.1387)** |
| 0.0051(-0.0331,0.0432) | **-0.1141(-0.1502,-0.0781)** | **-0.1108(-0.1571,-0.0645)** | **-0.2581(-0.3339,-0.1822)** |
| -0.0159(-0.0851,0.0533) | **-0.1014(-0.1605,-0.0423)** | **-0.1599(-0.2189,-0.101)** | **-0.1794(-0.2873,-0.0714)** |
| -**0.0226(-0.0361,-0.009)** | **-0.0384(-0.0603,-0.0165)** | **-0.0876(-0.1202,-0.0549)** | **-0.1174(-0.1492,-0.0855)** |

***(b) High-resource connected ecosystems***

*Local high resource ecosystem*

| 0% | 50% | 70% | 90% |
| --- | --- | --- | --- |
| 8e-04(-0.0133,0.015) | -0.016(-0.0441,0.0122) | -0.022(-0.0614,0.0173) | **-0.0728(-0.1171,-0.0285)** |
| -0.0076(-0.0349,0.0196) | -0.0309(-0.0703,0.0084) | **-0.0516(-0.0707,-0.0324)** | **-0.1078(-0.1454,-0.0701)** |
| **-0.0292(-0.0476,-0.0108)** | **-0.0494(-0.0703,-0.0285)** | **-0.0543(-0.0853,-0.0233)** | **-0.099(-0.1529,-0.045)** |
| **-0.0386(-0.0661,-0.0111)** | **-0.0398(-0.0746,-0.0051)** | **-0.0438(-0.07,-0.0176)** | **-0.0611(-0.1031,-0.019)** |
| -0.0034(-0.0528,0.046) | -0.0467(-0.1155,0.0221) | **-0.0356(-0.0646,-0.0067)** | -0.0186(-0.0523,0.0151) |
| 0.0034(-0.055,0.0618) | -0.01(-0.0416,0.0216) | -0.0035(-0.0297,0.0228) | -0.0158(-0.0496,0.018) |
| 0.0295(-0.014,0.073) | 0.001(-0.0311,0.0332) | 0.0254(-0.0134,0.0643) | 0.0321(-0.0112,0.0755) |
| 0.0425(-0.0128,0.0977) | 0.04(-0.0111,0.091) | 0.04(-0.0135,0.0936) | 0.0254(-0.0241,0.0749) |
| 0.0497(-0.0034,0.1028) | **0.0466(1e-04,0.0932)** | 0.0418(-0.0022,0.0857) | 0.0194(-0.0289,0.0676) |
| 0.0446(-9e-04,0.09) | **0.0456(0.0069,0.0843)** | 0.0349(-0.0054,0.0751) | 0.0152(-0.0231,0.0536) |

**Table S5-A**: Statistical summary output from repeated-measures linear mixed effect model using density (log-transformed) as a response variable and disturbance intensity and subsidy flow as fixed predicted variables, time as a repeated measure and replicate as a random effect. We report regression estimate ± standard error (S.E.), degrees of freedom (df), value of the t-statistic and p-value. p-values below 0.05 are indicated in bold and represent significant effects. Statistical analysis is performed for the local low- and high-resource ecosystems separately. The results using the patch 2 experimental units are reported in Supplementary Table S5-B.

***(a) Local low-resource ecosystems*** (Shapiro-Wilk normality test: W = 1.00, p = 0.093)

|  | Estimate ± S.E. | df | t-value | p-value |
| --- | --- | --- | --- | --- |
| Low subsidy flow | 0.12 ± 0.10 | 751.01 | 1.29 | 0.196 |
| **High subsidy flow** | **0.67 ± 0.10** | **751.02** | **6.98** | **< 0.001** |
| **Disturbance 50** | **-0.92 ± 0.10** | **751.02** | **-9.41** | **< 0.001** |
| **Disturbance 70** | **-1.47 ± 0.10** | **751.02** | **-15.19** | **< 0.001** |
| **Disturbance 90** | **-1.58 ± 0.10** | **751.05** | **-16.26** | **< 0.001** |
| Low resource SF x Dist. 50 | -0.06 ± 0.14 | 751.01 | -0.47 | 0.638 |
| Low resource SF x Dist. 70 | -0.02 ± 0.14 | 751.03 | -0.15 | 0.881 |
| Low resource SF x Dist. 90 | 0.06 ± 0.14 | 751.02 | 0.46 | 0.649 |
| High resource SF x Dist. 50 | -0.15 ± 0.14 | 751.03 | -1.09 | 0.277 |
| High resource SF x Dist. 70 | 0.09 ± 0.14 | 751.03 | 0.69 | 0.492 |
| High resource SF x Dist. 90 | -0.23 ± 0.14 | 751.07 | -1.64 | 0.101 |

***(b) Local high-resource ecosystems*** (Shapiro-Wilk normality test: W = 0.99, p < 0.001)

|  | Estimate ± S.E. | df | t-value | p-value |
| --- | --- | --- | --- | --- |
| Low subsidy flow | 0.01 ± 0.12 | 732.05 | 0.12 | 0.906 |
| High subsidy flow | 0.16 ± 0.12 | 732.00 | 1.31 | 0.190 |
| Disturbance 50 | 0.15 ± 0.12 | 731.99 | 1.23 | 0.221 |
| **Disturbance 70** | **0.62 ± 0.12** | **732.12** | **5.07** | **< 0.001** |
| **Disturbance 90** | **0.81 ± 0.12** | **732.07** | **6.67** | **< 0.001** |
| **Low resource SF x Dist. 50** | **0.49 ± 0.17** | **732.05** | **2.82** | **0.005** |
| Low resource SF x Dist. 70 | -0.16 ± 0.17 | 732.11 | -0.90 | 0.367 |
| Low resource SF x Dist. 90 | -0.14 ± 0.17 | 732.11 | -0.78 | 0.436 |
| **High resource SF x Dist. 50** | **0.41 ± 0.17** | **732.00** | **2.38** | **0.017** |
| High resource SF x Dist. 70 | 0.02 ± 0.17 | 732.14 | 0.12 | 0.903 |
| High resource SF x Dist. 90 | -0.25 ± 0.17 | 732.10 | -1.44 | 0.150 |

**Table S5-B**: Statistical summary output from repeated-measures linear mixed effect model using density (log-transformed) as a response variable and disturbance intensity and subsidy flow as fixed predicted variables, time as a repeated measure and replicate as a random effect. We report regression estimate ± standard error (S.E.), degrees of freedom (df), value of the t-statistic and p-value. p-values below 0.05 are indicated in bold and represent significant effects. Statistical analysis is performed for the local low- and high-resource ecosystems separately.

***(a) Local low-resource ecosystems*** (Shapiro-Wilk normality test: W = 0.99, p = 0.005)

|  | Estimate ± S.E. | df | t-value | p-value |
| --- | --- | --- | --- | --- |
| Low subsidy flow | -0.01 ± 0.10 | 741.89 | -0.12 | 0.904 |
| **High subsidy flow** | **0.67 ± 0.10** | **741.98** | **6.84** | **< 0.001** |
| **Disturbance 50** | **-0.92 ± 0.10** | **742.25** | **-9.23** | **< 0.001** |
| **Disturbance 70** | **-1.47 ± 0.10** | **742.03** | **-14.90** | **< 0.001** |
| **Disturbance 90** | **-1.58 ± 0.10** | **742.09** | **-15.95** | **< 0.001** |
| Low resource SF x Dist. 50 | -0.21 ± 0.14 | 742.06 | -1.49 | 0.136 |
| Low resource SF x Dist. 70 | 0.10 ± 0.14 | 742.24 | 0.70 | 0.483 |
| **Low resource SF x Dist. 90** | **0.31 ± 0.14** | **742.07** | **2.18** | **0.030** |
| High resource SF x Dist. 50 | -0.15 ± 0.14 | 742.28 | -1.06 | 0.289 |
| High resource SF x Dist. 70 | 0.10 ± 0.14 | 742.05 | 0.69 | 0.493 |
| High resource SF x Dist. 90 | -0.22 ± 0.14 | 742.07 | -1.59 | 0.112 |

***(b) Local high-resource ecosystems*** (Shapiro-Wilk normality test: W = 1.00, p = 0.022)

|  | Estimate ± S.E. | df | t-value | p-value |
| --- | --- | --- | --- | --- |
| Low subsidy flow | 0.01 ± 0.12 | 727.10 | 0.11 | 0.914 |
| High subsidy flow | 0.06 ± 0.12 | 727.24 | 0.51 | 0.609 |
| Disturbance 50 | 0.15 ± 0.12 | 727.04 | 1.27 | 0.204 |
| **Disturbance 70** | **0.62 ± 0.12** | **727.22** | **5.21** | **< 0.001** |
| **Disturbance 90** | **0.82 ± 0.12** | **727.15** | **6.87** | **< 0.001** |
| **Low resource SF x Dist. 50** | **0.49 ± 0.17** | **727.11** | **2.90** | **0.004** |
| Low resource SF x Dist. 70 | -0.16 ± 0.17 | 727.20 | -0.93 | 0.351 |
| Low resource SF x Dist. 90 | -0.14 ± 0.17 | 727.18 | -0.80 | 0.426 |
| High resource SF x Dist. 50 | 0.12 ± 0.17 | 727.30 | 0.73 | 0.465 |
| High resource SF x Dist. 70 | -0.16 ± 0.17 | 727.18 | -0.95 | 0.341 |
| High resource SF x Dist. 90 | -0.18 ± 0.17 | 727.19 | -1.08 | 0.283 |

**Table S6-A**: Log-response ratio of log-transformed density comparing each time point to the first time point for (a) isolated ecosystems, (b) ecosystems connected to a low-resource ecosystem and (c) ecosystems connected to a high-resource ecosystem. The first value gives the log-response ratio estimate and values between brackets indicate the 95% confidence interval. Values in bold reflect where the 95% confidence interval does not overlap with zero. The results using the patch 2 experimental units are reported in Supplementary Table S6-B.

***(a) Unconnected meta-ecosystems***

*Low resource ecosystem*

| 0% | 50% | 70% | 90% |
| --- | --- | --- | --- |
| **0.0605(0.0264,0.0947)** | -0.0044(-0.041,0.0322) | **-0.0326(-0.0563,-0.0089)** | **-0.1166(-0.1335,-0.0998)** |
| **0.0775(0.0497,0.1052)** | 0.0181(-0.0079,0.0441) | -0.0082(-0.0255,0.0092) | **-0.0842(-0.1263,-0.0422)** |
| **0.0818(0.0478,0.1158)** | 0.008(-0.0105,0.0265) | -0.0371(-0.0754,0.0012) | **-0.1248(-0.1599,-0.0896)** |
| **0.0889(0.0651,0.1127)** | 0.0206(-0.0162,0.0574) | **-0.0885(-0.1408,-0.0363)** | **-0.1557(-0.1925,-0.1189)** |
| **0.0853(0.0645,0.1062)** | -0.0143(-0.0567,0.0282) | **-0.1425(-0.161,-0.124)** | **-0.1194(-0.1605,-0.0782)** |
| **0.0788(0.0577,0.0999)** | **-0.0557(-0.1028,-0.0086)** | **-0.1255(-0.1777,-0.0733)** | **-0.1407(-0.187,-0.0945)** |
| **0.065(0.0239,0.1061)** | **-0.0871(-0.1336,-0.0407)** | **-0.1628(-0.1914,-0.1341)** | **-0.1055(-0.1324,-0.0786)** |
| 0.0366(-0.0305,0.1036) | **-0.0915(-0.1162,-0.0668)** | **-0.2141(-0.2467,-0.1815)** | **-0.2353(-0.3378,-0.1327)** |
| 0.0459(-0.0173,0.1092) | **-0.1545(-0.2003,-0.1087)** | **-0.2239(-0.2532,-0.1947)** | **-0.1793(-0.2272,-0.1313)** |
| 0.051(2e-04,0.1017) | **-0.1288(-0.2255,-0.0321)** | **-0.2458(-0.2745,-0.2171)** | **-0.1816(-0.2402,-0.123)** |

*High resource ecosystem*

| 0% | 50% | 70% | 90% |
| --- | --- | --- | --- |
| 0.0278(-0.0221,0.0776) | -0.0069(-0.0489,0.0351) | 0.004(-0.0526,0.0607) | 0.0044(-0.0739,0.0826) |
| 0.0024(-0.049,0.0538) | **-0.0286(-0.0481,-0.0092)** | 0.0359(-0.0094,0.0813) | 0.0312(-0.0272,0.0896) |
| 0.0076(-0.0534,0.0687) | 0.0113(-0.0512,0.0739) | **0.0837(0.0229,0.1445)** | **0.0667(0.0064,0.1269)** |
| 0.0032(-0.0514,0.0578) | 0.0293(-0.0282,0.0867) | **0.1009(0.0354,0.1663)** | **0.0949(0.0377,0.152)** |
| 0.0271(-0.0343,0.0885) | **0.0527(0.0083,0.0971)** | **0.1067(0.0376,0.1759)** | **0.1286(0.0558,0.2014)** |
| 0.042(-0.018,0.102) | **0.0604(0.0188,0.102)** | **0.1173(0.0311,0.2035)** | **0.147(0.1001,0.1939)** |
| 0.0399(-0.0428,0.1225) | **0.0749(0.0116,0.1382)** | **0.1088(0.0214,0.1963)** | **0.1706(0.117,0.2243)** |
| 0.0246(-0.059,0.1081) | **0.0828(0.0164,0.1493)** | **0.1351(0.0381,0.2321)** | **0.1672(0.1049,0.2294)** |
| 0.0219(-0.0562,0.1) | 0.0601(-0.004,0.1242) | **0.1289(0.0536,0.2042)** | **0.1666(0.1208,0.2124)** |
| 0.0142(-0.0851,0.1135) | 0.0515(-0.0161,0.119) | **0.1175(0.0351,0.2)** | **0.163(0.1104,0.2156)** |

***(b) Low-resource connected meta-ecosystems***

*Low resource ecosystem*

| 0% | 50% | 70% | 90% |
| --- | --- | --- | --- |
| 0.0299(-0.0019,0.0617) | 0.0112(-0.0123,0.0346) | **-0.0348(-0.0529,-0.0166)** | **-0.1056(-0.1356,-0.0757)** |
| **0.0587(0.0261,0.0913)** | 0.0332(-0.004,0.0703) | -0.015(-0.0309,9e-04) | **-0.0547(-0.094,-0.0154)** |
| **0.0662(0.0307,0.1016)** | 0.0221(-0.0313,0.0754) | **-0.0246(-0.0486,-6e-04)** | **-0.1063(-0.1421,-0.0705)** |
| **0.0859(0.0524,0.1195)** | 0.0118(-0.0403,0.064) | **-0.0745(-0.1116,-0.0374)** | **-0.1439(-0.1694,-0.1185)** |
| **0.0936(0.0646,0.1227)** | -0.0145(-0.0784,0.0495) | **-0.116(-0.1507,-0.0813)** | **-0.1059(-0.1573,-0.0545)** |
| **0.0892(0.0626,0.1159)** | -0.0523(-0.1139,0.0093) | **-0.1246(-0.1626,-0.0867)** | **-0.1107(-0.1815,-0.04)** |
| **0.0699(0.0456,0.0942)** | **-0.1029(-0.17,-0.0357)** | **-0.1871(-0.225,-0.1492)** | **-0.0847(-0.1321,-0.0373)** |
| **0.0771(0.0515,0.1028)** | **-0.1312(-0.2001,-0.0623)** | **-0.1985(-0.2538,-0.1433)** | **-0.1327(-0.1629,-0.1024)** |
| **0.0873(0.0547,0.1199)** | **-0.1578(-0.219,-0.0967)** | **-0.2178(-0.2482,-0.1875)** | **-0.2044(-0.2408,-0.168)** |
| **0.0734(0.0547,0.092)** | **-0.1776(-0.2139,-0.1413)** | **-0.2475(-0.2765,-0.2186)** | **-0.186(-0.209,-0.1629)** |

*High resource ecosystem*

| 0% | 50% | 70% | 90% |
| --- | --- | --- | --- |
| 0.0181(-0.036,0.0722) | 0.0213(-0.0344,0.077) | -0.0164(-0.0668,0.034) | 0.0111(-0.0832,0.1054) |
| -0.0188(-0.1116,0.0739) | -6e-04(-0.0658,0.0645) | **-0.0513(-0.0928,-0.0099)** | -0.0127(-0.085,0.0595) |
| -0.0452(-0.14,0.0496) | 0.0282(-0.0246,0.0809) | 0.0041(-0.0413,0.0495) | -0.0079(-0.0686,0.0528) |
| 0.0041(-0.0918,0.0999) | 0.0552(-0.0031,0.1136) | -0.0089(-0.0376,0.0199) | 0.0223(-0.0424,0.087) |
| -0.0222(-0.0974,0.0529) | 0.0617(-0.0095,0.133) | 0.0378(-0.012,0.0876) | **0.083(0.0106,0.1554)** |
| -0.0103(-0.0921,0.0715) | **0.0691(0.0026,0.1356)** | **0.0806(0.0367,0.1245)** | **0.1084(0.0339,0.1829)** |
| -0.0132(-0.0987,0.0722) | **0.0872(0.0123,0.1621)** | **0.0872(0.0044,0.1701)** | **0.1161(0.0205,0.2118)** |
| -0.0282(-0.1025,0.0461) | **0.1055(0.0211,0.1898)** | **0.1115(0.0521,0.1709)** | **0.1235(0.0655,0.1816)** |
| -0.0624(-0.154,0.0291) | **0.0882(0.0026,0.1738)** | **0.081(0.0085,0.1535)** | **0.1068(0.0489,0.1647)** |
| 0.0031(-0.075,0.0811) | **0.0802(0.0105,0.1498)** | **0.0794(0.0149,0.1438)** | **0.1055(0.0482,0.1627)** |

***(c) High-resource connected meta-ecosystems***

*Low resource ecosystem*

| 0% | 50% | 70% | 90% |
| --- | --- | --- | --- |
| **0.0387(0.0096,0.0678)** | 0.003(-0.0152,0.0213) | -0.0081(-0.0369,0.0208) | **-0.082(-0.1158,-0.0483)** |
| **0.0698(0.0268,0.1129)** | **0.04(0.009,0.071)** | 0.0085(-0.0093,0.0263) | -0.0187(-0.0654,0.0279) |
| **0.0923(0.0539,0.1307)** | **0.0335(0.0013,0.0658)** | 0.0028(-0.0232,0.0287) | **-0.0783(-0.1258,-0.0308)** |
| **0.1287(0.1026,0.1547)** | 0.0345(-0.0079,0.077) | -0.0027(-0.0232,0.0179) | **-0.0895(-0.1343,-0.0447)** |
| **0.1389(0.1109,0.1669)** | 0.0412(-0.0047,0.0871) | **-0.0251(-0.0459,-0.0044)** | **-0.0698(-0.1369,-0.0027)** |
| **0.1506(0.1023,0.1989)** | 0.0116(-0.0372,0.0604) | -0.0161(-0.051,0.0187) | **-0.0678(-0.1277,-0.0079)** |
| **0.1568(0.0957,0.218)** | -0.0029(-0.0457,0.0399) | -0.0409(-0.0832,0.0014) | **-0.0595(-0.1082,-0.0108)** |
| **0.1703(0.126,0.2146)** | 0.0074(-0.0329,0.0477) | **-0.0804(-0.1229,-0.0379)** | **-0.1126(-0.171,-0.0542)** |
| **0.1962(0.1573,0.2351)** | -0.0068(-0.0705,0.057) | -0.0362(-0.0754,0.003) | **-0.15(-0.2504,-0.0496)** |
| **0.2304(0.2025,0.2582)** | 0.0017(-0.0621,0.0655) | **-0.0612(-0.1145,-0.008)** | **-0.1472(-0.2033,-0.0912)** |

*High resource ecosystem*

| 0% | 50% | 70% | 90% |
| --- | --- | --- | --- |
| 0.0168(-0.0224,0.056) | 0.0218(-0.0236,0.0672) | 0.0301(-0.0322,0.0925) | -0.0338(-0.1041,0.0366) |
| -0.0217(-0.0839,0.0404) | 0.0327(-0.0091,0.0746) | 0.0162(-0.034,0.0664) | -0.044(-0.0883,2e-04) |
| -0.0284(-0.0766,0.0198) | 0.0335(-0.0431,0.1102) | 0.0284(-0.0492,0.1059) | 0.0068(-0.0403,0.054) |
| -0.0182(-0.073,0.0366) | 0.0422(-0.0194,0.1038) | 0.0633(-0.0434,0.17) | 0.0296(-0.0156,0.0748) |
| -0.0046(-0.075,0.0658) | 0.0552(-0.0095,0.1199) | 0.065(-0.0357,0.1657) | **0.1078(0.0635,0.152)** |
| 0.0117(-0.0581,0.0814) | **0.0632(0.0164,0.1101)** | **0.1055(0.0169,0.1942)** | **0.1146(0.0654,0.1639)** |
| 0.0379(-0.0322,0.108) | **0.1217(0.0879,0.1554)** | **0.1085(0.0208,0.1963)** | **0.1343(0.1057,0.1629)** |
| 0.0276(-0.037,0.0922) | **0.124(0.0872,0.1607)** | **0.1349(0.0387,0.231)** | **0.1362(0.0913,0.1811)** |
| 0.0431(-0.0129,0.0991) | **0.1268(0.0781,0.1754)** | **0.1228(0.0354,0.2102)** | **0.1201(0.0788,0.1614)** |
| 0.0144(-0.0628,0.0916) | **0.0927(0.022,0.1633)** | **0.1099(0.0274,0.1925)** | **0.1394(0.0924,0.1864)** |

**Table S6-B**: Log-response ratio of log-transformed density comparing each time point to the first time point for (a) isolated ecosystems, (b) ecosystems connected to a low-resource ecosystem and (c) ecosystems connected to a high-resource ecosystem. The first value gives the log-response ratio estimate and values between brackets indicate the 95% confidence interval. Values in bold reflect where the 95% confidence interval does not overlap with zero.

***(a) Low-resource connected meta-ecosystems***

*Low resource ecosystem*

| 0.0189(-0.0092,0.047) | -0.0234(-0.0566,0.0098) | **-0.0214(-0.0414,-0.0014)** | **-0.0922(-0.1122,-0.0721)** |
| --- | --- | --- | --- |
| **0.0464(0.0167,0.0761)** | 0.0276(-0.0042,0.0594) | -0.0054(-0.0177,0.0069) | **-0.0422(-0.0658,-0.0186)** |
| **0.0589(0.0318,0.086)** | 8e-04(-0.0305,0.0321) | **-0.0322(-0.0601,-0.0043)** | **-0.0963(-0.1402,-0.0523)** |
| **0.085(0.0635,0.1066)** | -0.0092(-0.0606,0.0422) | **-0.0749(-0.1093,-0.0406)** | **-0.1092(-0.1343,-0.0842)** |
| **0.0965(0.0748,0.1181)** | -0.0592(-0.1349,0.0166) | **-0.1177(-0.1643,-0.0712)** | **-0.1192(-0.141,-0.0975)** |
| **0.0836(0.0555,0.1117)** | **-0.0907(-0.1634,-0.0179)** | **-0.1197(-0.1762,-0.0631)** | **-0.0953(-0.1371,-0.0535)** |
| **0.0722(0.029,0.1155)** | **-0.1565(-0.2051,-0.1078)** | **-0.164(-0.2235,-0.1045)** | **-0.0961(-0.1281,-0.0641)** |
| **0.0622(0.0053,0.1191)** | **-0.1794(-0.215,-0.1437)** | **-0.1893(-0.2496,-0.1289)** | **-0.1169(-0.135,-0.0988)** |
| **0.0729(0.0234,0.1223)** | **-0.1696(-0.2041,-0.1352)** | **-0.2174(-0.2887,-0.1461)** | **-0.1518(-0.1841,-0.1196)** |
| 0.0505(-0.0229,0.1239) | **-0.1411(-0.2635,-0.0186)** | **-0.2156(-0.2705,-0.1607)** | -0.0877(-0.2054,0.03) |

***(b) High-resource connected meta-ecosystems***

*High resource ecosystem*

|  |  |  |  |
| --- | --- | --- | --- |
| -0.011(-0.0734,0.0514) | -0.0032(-0.0703,0.064) | -0.0093(-0.0737,0.0551) | -0.0326(-0.1013,0.036) |
| -0.0091(-0.0777,0.0596) | -0.0024(-0.0638,0.0589) | -0.0034(-0.0482,0.0413) | -0.0187(-0.0726,0.0351) |
| -0.0311(-0.0738,0.0116) | -0.0014(-0.0551,0.0522) | 0.0364(-0.006,0.0788) | 0.0339(-0.0264,0.0942) |
| **-0.0331(-0.0563,-0.0098)** | 0.0031(-0.0546,0.0608) | 0.04(-0.008,0.0879) | **0.083(0.0248,0.1412)** |
| 0.0029(-0.0823,0.0882) | 0.018(-0.0986,0.1345) | **0.0499(0.0055,0.0943)** | **0.1257(0.0858,0.1657)** |
| -0.0044(-0.1155,0.1067) | 0.0304(-0.0464,0.1073) | **0.0887(0.045,0.1324)** | **0.1048(0.059,0.1505)** |
| 0.0282(-0.0619,0.1183) | 0.0446(-0.0157,0.105) | **0.1221(0.0651,0.1791)** | **0.1592(0.0956,0.2228)** |
| 0.039(-0.05,0.1281) | **0.0959(0.0286,0.1633)** | **0.1184(0.0493,0.1875)** | **0.1249(0.0744,0.1753)** |
| 0.0513(-0.0162,0.1187) | **0.1189(0.0656,0.1722)** | **0.1123(0.0529,0.1717)** | **0.1164(0.061,0.1717)** |
| **0.0661(0.0133,0.1189)** | **0.106(0.0496,0.1624)** | **0.1124(0.0663,0.1585)** | **0.1238(0.0762,0.1714)** |

**Table S7-A**: Log-response ratio of density (log-transformed) at each time point comparing each experimental treatment combination to the control, undisturbed isolated local low-resource ecosystem for (a) isolated ecosystems, (b) ecosystems connected to a low-resource ecosystem and (c) ecosystems connected to a high-resource ecosystem. The first value gives the log-response ratio estimate and values between brackets indicate the 95% confidence interval. Values in bold reflect where the 95% confidence interval does not overlap with zero. The results using the patch 2 experimental units are reported in Supplementary Table S7-B.

***(a) Unconnected meta-ecosystems***

*Low resource ecosystem*

| 0% | 50% | 70% | 90% |
| --- | --- | --- | --- |
|  | **-0.0649(-0.1127,-0.0172)** | **-0.0931(-0.1319,-0.0543)** | **-0.1772(-0.2122,-0.1421)** |
|  | **-0.0594(-0.0943,-0.0244)** | **-0.0856(-0.1147,-0.0566)** | **-0.1617(-0.2098,-0.1136)** |
|  | **-0.0738(-0.1095,-0.0381)** | **-0.1189(-0.1679,-0.0699)** | **-0.2066(-0.2531,-0.16)** |
|  | **-0.0683(-0.1095,-0.0271)** | **-0.1774(-0.2329,-0.122)** | **-0.2446(-0.2858,-0.2034)** |
|  | **-0.0996(-0.1445,-0.0548)** | **-0.2279(-0.2513,-0.2044)** | **-0.2047(-0.2483,-0.1611)** |
|  | **-0.1345(-0.1839,-0.0851)** | **-0.2043(-0.2585,-0.15)** | **-0.2195(-0.2681,-0.1709)** |
|  | **-0.1522(-0.2123,-0.092)** | **-0.2278(-0.2756,-0.18)** | **-0.1705(-0.2173,-0.1238)** |
|  | **-0.1281(-0.198,-0.0583)** | **-0.2507(-0.3237,-0.1777)** | **-0.2718(-0.3934,-0.1502)** |
|  | **-0.2005(-0.2771,-0.1238)** | **-0.2699(-0.3379,-0.2018)** | **-0.2252(-0.3032,-0.1473)** |
|  | **-0.1798(-0.2879,-0.0716)** | **-0.2968(-0.3531,-0.2405)** | **-0.2325(-0.3086,-0.1565)** |

*High resource ecosystem*

| -0.0328(-0.09,0.0244) | **-0.0674(-0.1179,-0.0169)** | -0.0565(-0.1197,0.0067) | -0.0562(-0.1393,0.0269) |
| --- | --- | --- | --- |
| **-0.0751(-0.1302,-0.02)** | **-0.1061(-0.1338,-0.0784)** | -0.0416(-0.091,0.0079) | -0.0463(-0.108,0.0153) |
| **-0.0741(-0.1413,-0.007)** | **-0.0704(-0.1389,-0.002)** | 0.0019(-0.065,0.0687) | -0.0151(-0.0815,0.0512) |
| **-0.0857(-0.142,-0.0294)** | **-0.0596(-0.1187,-5e-04)** | 0.012(-0.0549,0.0788) | 0.006(-0.0528,0.0647) |
| -0.0582(-0.12,0.0036) | -0.0326(-0.0777,0.0124) | 0.0214(-0.0482,0.0909) | 0.0433(-0.0299,0.1164) |
| -0.0368(-0.0973,0.0237) | -0.0184(-0.0608,0.024) | 0.0385(-0.0481,0.1251) | **0.0682(0.0206,0.1158)** |
| -0.0252(-0.1154,0.0651) | 0.0099(-0.063,0.0828) | 0.0438(-0.0508,0.1384) | **0.1056(0.0409,0.1703)** |
| -0.012(-0.1174,0.0933) | 0.0462(-0.0461,0.1386) | 0.0985(-0.0178,0.2148) | **0.1306(0.0412,0.2199)** |
| -0.024(-0.1226,0.0745) | 0.0141(-0.0738,0.102) | 0.083(-0.0134,0.1794) | **0.1207(0.045,0.1963)** |
| -0.0367(-0.1465,0.073) | 5e-04(-0.0817,0.0827) | 0.0666(-0.0282,0.1614) | **0.1121(0.0416,0.1825)** |

***(b) Low-resource connected meta-ecosystems***

*Low resource ecosystem*

| -0.0413(-0.083,4e-04) | **-0.0836(-0.1289,-0.0383)** | **-0.0816(-0.1183,-0.0448)** | **-0.1524(-0.1891,-0.1156)** |
| --- | --- | --- | --- |
| -0.0307(-0.0686,0.0071) | **-0.0495(-0.0891,-0.01)** | **-0.0826(-0.109,-0.0561)** | **-0.1193(-0.1526,-0.0861)** |
| -0.0225(-0.0635,0.0184) | **-0.0806(-0.1244,-0.0369)** | **-0.1137(-0.1551,-0.0722)** | **-0.1777(-0.2313,-0.1241)** |
| -0.0035(-0.032,0.0251) | **-0.0978(-0.1525,-0.0431)** | **-0.1635(-0.2026,-0.1244)** | **-0.1978(-0.229,-0.1665)** |
| 0.0115(-0.0147,0.0376) | **-0.1441(-0.2213,-0.067)** | **-0.2027(-0.2515,-0.154)** | **-0.2042(-0.2304,-0.178)** |
| 0.0051(-0.0267,0.037) | **-0.1691(-0.2433,-0.0948)** | **-0.1981(-0.2566,-0.1396)** | **-0.1737(-0.2181,-0.1293)** |
| 0.0076(-0.0502,0.0654) | **-0.2211(-0.2831,-0.1592)** | **-0.2287(-0.2995,-0.1579)** | **-0.1608(-0.2107,-0.1108)** |
| 0.026(-0.0607,0.1126) | **-0.2156(-0.2901,-0.1411)** | **-0.2255(-0.3145,-0.1365)** | **-0.1531(-0.221,-0.0853)** |
| 0.0273(-0.0516,0.1062) | **-0.2152(-0.2857,-0.1448)** | **-0.263(-0.3572,-0.1688)** | **-0.1974(-0.2668,-0.128)** |
| -1e-04(-0.0881,0.0879) | **-0.1917(-0.3234,-0.06)** | **-0.2662(-0.3395,-0.1929)** | **-0.1383(-0.2656,-0.011)** |

*High resource ecosystem*

| -0.0066(-0.0672,0.054) | -0.0034(-0.0655,0.0587) | -0.0411(-0.0984,0.0162) | -0.0136(-0.1117,0.0846) |
| --- | --- | --- | --- |
| -0.0604(-0.1551,0.0342) | -0.0422(-0.11,0.0255) | -0.0929(-0.1384,-0.0475) | -0.0543(-0.129,0.0203) |
| -0.0911(-0.1898,0.0075) | -0.0178(-0.0771,0.0416) | -0.0418(-0.0948,0.0111) | -0.0538(-0.1203,0.0127) |
| -0.0489(-0.1456,0.0477) | 0.0022(-0.0574,0.0618) | -0.0619(-0.0931,-0.0307) | -0.0307(-0.0965,0.0351) |
| -0.0717(-0.147,0.0035) | 0.0123(-0.0591,0.0836) | -0.0116(-0.0616,0.0383) | 0.0335(-0.039,0.106) |
| -0.0532(-0.1352,0.0288) | 0.0262(-0.0405,0.0928) | 0.0377(-0.0065,0.0819) | 0.0655(-0.0092,0.1402) |
| -0.0424(-0.135,0.0502) | 0.0581(-0.0249,0.141) | 0.0581(-0.0321,0.1482) | 0.087(-0.0151,0.1891) |
| -0.0289(-0.1268,0.0691) | 0.1048(-0.001,0.2105) | **0.1108(0.0236,0.198)** | **0.1228(0.0365,0.2091)** |
| -0.0725(-0.1819,0.0369) | 0.0781(-0.0263,0.1826) | 0.0709(-0.0231,0.1649) | **0.0967(0.0134,0.18)** |
| -0.012(-0.1028,0.0788) | 0.0651(-0.0186,0.1488) | 0.0643(-0.0151,0.1437) | **0.0904(0.0167,0.1641)** |

***(c) High-resource connected meta-ecosystems***

*Low resource ecosystem*

| -0.0173(-0.0594,0.0248) | -0.053(-0.0885,-0.0175) | **-0.0641(-0.106,-0.0222)** | **-0.1381(-0.1835,-0.0926)** |
| --- | --- | --- | --- |
| -0.0031(-0.0519,0.0456) | -0.033(-0.0716,0.0056) | **-0.0645(-0.0935,-0.0355)** | **-0.0917(-0.1437,-0.0398)** |
| 0.015(-0.0339,0.0639) | -0.0437(-0.088,5e-04) | **-0.0745(-0.1144,-0.0347)** | **-0.1556(-0.2119,-0.0992)** |
| **0.0443(0.0126,0.0759)** | **-0.0498(-0.0959,-0.0038)** | **-0.0871(-0.1144,-0.0597)** | **-0.1739(-0.2221,-0.1256)** |
| **0.0581(0.0269,0.0892)** | -0.0397(-0.0876,0.0083) | **-0.106(-0.1309,-0.081)** | **-0.1507(-0.2192,-0.0821)** |
| **0.0764(0.026,0.1267)** | **-0.0627(-0.1135,-0.0119)** | **-0.0904(-0.1281,-0.0528)** | **-0.142(-0.2036,-0.0805)** |
| **0.0963(0.0243,0.1683)** | **-0.0634(-0.1206,-0.0062)** | **-0.1014(-0.1583,-0.0446)** | **-0.12(-0.1818,-0.0582)** |
| **0.1382(0.0594,0.2171)** | -0.0247(-0.1013,0.052) | **-0.1125(-0.1903,-0.0347)** | **-0.1447(-0.2322,-0.0571)** |
| **0.1548(0.0822,0.2274)** | -0.0482(-0.1366,0.0402) | **-0.0776(-0.1504,-0.0049)** | **-0.1915(-0.3091,-0.0738)** |
| **0.1839(0.1282,0.2396)** | -0.0447(-0.1247,0.0352) | **-0.1077(-0.1795,-0.0358)** | **-0.1937(-0.2677,-0.1197)** |

*High resource ecosystem*

| -0.0112(-0.0593,0.0369) | -0.0062(-0.0595,0.047) | 0.0021(-0.0662,0.0705) | -0.0618(-0.1375,0.0139) |
| --- | --- | --- | --- |
| -0.0667(-0.1318,-0.0016) | -0.0122(-0.0584,0.0339) | -0.0288(-0.0826,0.0251) | -0.089(-0.1373,-0.0407) |
| -0.0777(-0.1332,-0.0221) | -0.0157(-0.0972,0.0658) | -0.0209(-0.1032,0.0614) | -0.0424(-0.0971,0.0123) |
| -0.0746(-0.131,-0.0182) | -0.0142(-0.0772,0.0488) | 0.0069(-0.1006,0.1145) | -0.0267(-0.0738,0.0203) |
| -0.0574(-0.1281,0.0133) | 0.0024(-0.0627,0.0674) | 0.0122(-0.0888,0.1131) | **0.0549(0.0102,0.0996)** |
| -0.0346(-0.1047,0.0355) | 0.017(-0.0304,0.0644) | 0.0593(-0.0297,0.1482) | **0.0684(0.0186,0.1181)** |
| 0.0054(-0.0734,0.0842) | **0.0892(0.0399,0.1385)** | 0.0761(-0.0188,0.1709) | **0.1018(0.0558,0.1478)** |
| 0.0236(-0.0674,0.1146) | **0.1199(0.0461,0.1938)** | **0.1308(0.0153,0.2463)** | **0.1322(0.0539,0.2104)** |
| 0.0297(-0.0524,0.1118) | **0.1134(0.0361,0.1907)** | **0.1094(0.0034,0.2154)** | **0.1067(0.0338,0.1796)** |
| -0.0041(-0.0943,0.0862) | 0.0743(-0.0104,0.1589) | 0.0915(-0.0033,0.1863) | **0.121(0.0547,0.1873)** |

**Table S7-B**: Log-response ratio of density (log-transformed) at each time point comparing each experimental treatment combination to the control, undisturbed isolated local low-resource ecosystem for (a) isolated ecosystems, (b) ecosystems connected to a low-resource ecosystem and (c) ecosystems connected to a high-resource ecosystem using the patch 2 experimental units. The first value gives the log-response ratio estimate and values between brackets indicate the 95% confidence interval. Values in bold reflect where the 95% confidence interval does not overlap with zero.

***(a) Low-resource connected meta-ecosystems***

*Low resource ecosystem*

| -0.0413(-0.083,4e-04) | **-0.0836(-0.1289,-0.0383)** | **-0.0816(-0.1183,-0.0448)** | **-0.1524(-0.1891,-0.1156)** |
| --- | --- | --- | --- |
| -0.0307(-0.0686,0.0071) | **-0.0495(-0.0891,-0.01)** | **-0.0826(-0.109,-0.0561)** | **-0.1193(-0.1526,-0.0861)** |
| -0.0225(-0.0635,0.0184) | **-0.0806(-0.1244,-0.0369)** | **-0.1137(-0.1551,-0.0722)** | **-0.1777(-0.2313,-0.1241)** |
| -0.0035(-0.032,0.0251) | **-0.0978(-0.1525,-0.0431)** | **-0.1635(-0.2026,-0.1244)** | **-0.1978(-0.229,-0.1665)** |
| 0.0115(-0.0147,0.0376) | **-0.1441(-0.2213,-0.067)** | **-0.2027(-0.2515,-0.154)** | **-0.2042(-0.2304,-0.178)** |
| 0.0051(-0.0267,0.037) | **-0.1691(-0.2433,-0.0948)** | **-0.1981(-0.2566,-0.1396)** | **-0.1737(-0.2181,-0.1293)** |
| 0.0076(-0.0502,0.0654) | **-0.2211(-0.2831,-0.1592)** | **-0.2287(-0.2995,-0.1579)** | **-0.1608(-0.2107,-0.1108)** |
| 0.026(-0.0607,0.1126) | **-0.2156(-0.2901,-0.1411)** | **-0.2255(-0.3145,-0.1365)** | **-0.1531(-0.221,-0.0853)** |
| 0.0273(-0.0516,0.1062) | **-0.2152(-0.2857,-0.1448)** | **-0.263(-0.3572,-0.1688)** | **-0.1974(-0.2668,-0.128)** |
| -1e-04(-0.0881,0.0879) | **-0.1917(-0.3234,-0.06)** | **-0.2662(-0.3395,-0.1929)** | **-0.1383(-0.2656,-0.011)** |

***(b) High-resource connected meta-ecosystems***

*High resource ecosystem*

| -0.0536(-0.1222,0.015) | -0.0457(-0.1187,0.0273) | -0.0518(-0.1223,0.0186) | -0.0752(-0.1495,-8e-04) |
| --- | --- | --- | --- |
| -0.0686(-0.1402,0.003) | -0.0619(-0.1266,0.0028) | -0.0629(-0.1121,-0.0138) | -0.0782(-0.1358,-0.0206) |
| -0.0949(-0.1461,-0.0436) | -0.0652(-0.1259,-0.0046) | -0.0274(-0.0784,0.0237) | -0.0299(-0.0965,0.0368) |
| -0.104(-0.1315,-0.0765) | -0.0678(-0.1273,-0.0083) | -0.0309(-0.081,0.0192) | 0.0121(-0.0479,0.0722) |
| -0.0644(-0.1501,0.0213) | -0.0494(-0.1663,0.0675) | -0.0174(-0.0627,0.0278) | 0.0584(0.0174,0.0993) |
| -0.0652(-0.1767,0.0464) | -0.0303(-0.1078,0.0471) | 0.0279(-0.0168,0.0727) | 0.044(-0.0028,0.0907) |
| -0.0188(-0.116,0.0784) | -0.0024(-0.0729,0.0682) | **0.0751(0.0074,0.1428)** | **0.1122(0.0388,0.1855)** |
| 0.0204(-0.0894,0.1303) | 0.0773(-0.0158,0.1705) | **0.0998(0.0053,0.1942)** | **0.1063(0.0245,0.188)** |
| 0.0233(-0.0672,0.1139) | **0.091(0.0105,0.1715)** | 0.0844(-3e-04,0.1691) | **0.0884(0.0065,0.1703)** |
| 0.0331(-0.0376,0.1039) | 0.073(-5e-04,0.1465) | **0.0794(0.0135,0.1453)** | **0.0908(0.0239,0.1578)** |

**Table S8-A**: Statistical summary table from PERMANOVA on Hellinger-transformed size class abundances. Hellinger-transformed size class abundances are used as response variable, disturbance intensity, subsidy flow and time are used as fixed variables and replicate as a random factor. In the table we report degrees of freedom (df), sum of squares (SS), mean sum of squares (MS), F-statistic (F-value), R² and p-value. Significant effects (p < 0.005) are indicated in bold. Analysis is performed for the local low- and high-resource ecosystems separately. The results using the patch 2 experimental units are reported in Supplementary Table S5-B.

***(a) Low-resource ecosystem***

|  | df | SS | MS | F-value | R² | p-value |
| --- | --- | --- | --- | --- | --- | --- |
| **Disturbance (D)** | **3** | **5.10** | **1.70** | **82.47** | **0.208** | **< 0.001** |
| **Subsidy flow** | **2** | **0.22** | **0.11** | **5.22** | **0.009** | **< 0.001** |
| **Time** | **1** | **2.14** | **2.14** | **103.70** | **0.087** | **< 0.001** |
| **D x Subsidy flow** | **6** | **0.23** | **0.04** | **1.88** | **0.009** | **0.016** |
| **D x Time** | **3** | **1.18** | **0.39** | **19.09** | **0.048** | **< 0.001** |
| Subsidy flow x Time | 2 | 0.07 | 0.03 | 1.68 | 0.003 | 0.121 |
| D x Subsidy flow x Time | 6 | 0.20 | 0.03 | 1.58 | 0.008 | 0.050 |

***(b) High-resource ecosystem***

|  | df | SS | MS | F-value | R² | p-value |
| --- | --- | --- | --- | --- | --- | --- |
| **Disturbance (D)** | **3** | **3.66** | **1.22** | **122.90** | **0.301** | **< 0.001** |
| Subsidy flow | 2 | 0.04 | 0.02 | 1.86 | 0.003 | 0.088 |
| **Time** | **1** | **0.58** | **0.58** | **58.56** | **0.048** | **< 0.001** |
| **D x Subsidy Flow** | **6** | **0.23** | **0.04** | **3.82** | **0.019** | **< 0.001** |
| **D x Time** | **3** | **0.25** | **0.08** | **8.45** | **0.021** | **< 0.001** |
| Subsidy flow x Time | 2 | 0.02 | 0.01 | 0.97 | 0.002 | 0.414 |
| D x Subsidy flow x Time | 6 | 0.09 | 0.02 | 1.56 | 0.008 | 0.073 |

**Table S8-B**: Statistical summary table from PERMANOVA on Hellinger-transformed size class abundances using the patch 2 experimental units. Hellinger-transformed size class abundances are used as response variable, disturbance intensity, subsidy flow and time are used as fixed variables and replicate as a random factor. Analysis is performed for the local low- and high-resource ecosystems separately due to non-independence of some experimental treatments. In the table we report degrees of freedom (df), sum of squares (SS), mean sum of squares (MS), F-statistic (F-value), R² and p-value. Significant effects (p < 0.005) are indicated in bold.

***(a) Low-resource ecosystem***

|  | df | SS | MS | F-value | R² | p-value |
| --- | --- | --- | --- | --- | --- | --- |
| **Disturbance (D)** | **3** | **4.58** | **1.53** | **75.24** | **0.198** | **< 0.001** |
| **Subsidy flow** | **2** | **0.20** | **0.10** | **4.95** | **0.009** | **< 0.001** |
| **Time** | **1** | **1.92** | **1.92** | **94.86** | **0.083** | **< 0.001** |
| **D x Subsidy flow** | **6** | **0.22** | **0.04** | **1.78** | **0.009** | **0.029** |
| **D x Time** | **3** | **0.86** | **0.29** | **14.22** | **0.037** | **< 0.001** |
| Subsidy flow x Time | 2 | 0.05 | 0.02 | 1.19 | 0.002 | 0.306 |
| D x Subsidy flow x Time | 6 | 0.18 | 0.03 | 1.48 | 0.008 | 0.076 |

***(b) High-resource ecosystem***

|  | df | SS | MS | F-value | R² | p-value |
| --- | --- | --- | --- | --- | --- | --- |
| **Disturbance (D)** | **3** | **3.47** | **1.16** | **116.23** | **0.287** | **< 0.001** |
| **Subsidy flow** | **2** | **0.07** | **0.03** | **3.49** | **0.006** | **0.003** |
| **Time** | **1** | **0.66** | **0.66** | **65.96** | **0.054** | **< 0.001** |
| **D x Subsidy flow** | **6** | **0.24** | **0.04** | **3.99** | **0.020** | **< 0.001** |
| **D x Time** | **3** | **0.25** | **0.08** | **8.33** | **0.021** | **< 0.001** |
| Subsidy flow x Time | 2 | 0.03 | 0.02 | 1.28 | 0.002 | 0.266 |
| **D x Subsidy flow x Time** | **6** | **0.11** | **0.01** | **1.92** | **0.010** | **0.019** |

**Table S9-A**: Statistical summary output from repeated-measures linear mixed effect model using size class diversity as a response variable and disturbance intensity and subsidy flow as fixed predicted variables, time as a repeated measure and replicate as a random effect. We report regression estimate ± standard error (S.E.), degrees of freedom (df), value of the t-statistic and p-value. p-values below 0.05 are indicated in bold and represent significant effects. Statistical analysis is performed for the local low- and high-resource ecosystems separately, but including the isolated ecosystems as a control in both analyses. The results using the patch 2 experimental units are reported in Supplementary Table S9-B.

***(a) Local low-resource ecosystems*** (Shapiro-Wilk normality test: W = 0.97, p < 0.001)

|  | Estimate ± S.E. | df | t-value | p-value |
| --- | --- | --- | --- | --- |
| **Low subsidy flow** | **-0.14 ± 0.05** | **746.10** | **-2.78** | **0.006** |
| High subsidy flow | -0.06 ± 0.05 | 746.10 | -1.20 | 0.230 |
| **Disturbance 50** | **0.15 ± 0.05** | **746.21** | **2.78** | **0.006** |
| **Disturbance 70** | **0.38 ± 0.05** | **746.13** | **7.21** | **< 0.001** |
| **Disturbance 90** | **-0.14 ± 0.05** | **746.17** | **-2.74** | **0.006** |
| **Low subsidy flow x Dist. 50** | **0.16 ± 0.07** | **746.14** | **2.16** | **0.031** |
| **Low subsidy flow x Dist. 70** | **0.19 ± 0.07** | **746.02** | **2.52** | **0.012** |
| **Low subsidy flow x Dist. 90** | **0.16 ± 0.07** | **746.15** | **2.14** | **0.032** |
| High subsidy flow x Dist. 50 | 0.12 ± 0.07 | 746.23 | 1.67 | 0.096 |
| High subsidy flow x Dist. 70 | -0.13 ± 0.07 | 746.14 | -1.82 | 0.069 |
| High subsidy flow x Dist. 90 | 0.13 ± 0.07 | 746.18 | 1.81 | 0.071 |

***(b) Local high-resource ecosystems*** (Shapiro-Wilk normality test: W = 0.99, p < 0.001)

|  | Estimate ± S.E. | df | t-value | p-value |
| --- | --- | --- | --- | --- |
| **Low resource metaflow** | **-0.20 ± 0.05** | **732.13** | **-3.67** | **< 0.001** |
| **High resource metaflow** | **-0.37 ± 0.05** | **732.11** | **-6.94** | **< 0.001** |
| **Disturbance 50** | **-0.73 ± 0.05** | **732.02** | **-13.68** | **< 0.001** |
| **Disturbance 70** | **-0.89 ± 0.05** | **732.55** | **-16.48** | **< 0.001** |
| **Disturbance 90** | **-1.08 ± 0.05** | **732.35** | **-20.11** | **< 0.001** |
| Low subsidy flow x Dist. 50 | 0.02 ± 0.08 | 732.13 | 0.21 | 0.834 |
| **Low subsidy flow x Dist. 70** | **0.23 ± 0.08** | **732.49** | **2.99** | **0.003** |
| **Low subsidy flow x Dist. 90** | **0.15 ± 0.08** | **732.31** | **1.97** | **0.049** |
| **High subsidy flow x Dist. 50** | **0.32 ± 0.08** | **732.10** | **4.21** | **< 0.001** |
| **High subsidy flow x Dist. 70** | **0.29 ± 0.08** | **732.64** | **3.76** | **< 0.001** |
| **High subsidy flow x Dist. 90** | **0.29 ± 0.08** | **732.42** | **3.85** | **< 0.001** |

**Table S9-B**: Statistical summary output from repeated-measures linear mixed effect model using size class diversity as a response variable and disturbance intensity and subsidy flow as fixed predicted variables, time as a repeated measure and replicate as a random effect using the patch 2 experimental units. We report regression estimate ± standard error (S.E.), degrees of freedom (df), value of the t-statistic and p-value. p-values below 0.05 are indicated in bold and represent significant effects. Statistical analysis is performed for the local low- and high-resource ecosystems separately, but including the isolated ecosystems as a control in both analyses.

***(a) Local low-resource ecosystems*** (Shapiro-Wilk normality test: W = 0.98, p < 0.001)

|  | Estimate ± S.E. | df | t-value | p-value |
| --- | --- | --- | --- | --- |
| Low resource metaflow | -0.08 ± 0.05 | 742.00 | -1.56 | 0.120 |
| High resource metaflow | -0.06 ± 0.05 | 742.04 | -1.20 | 0.230 |
| **Disturbance 50** | **0.15 ± 0.05** | **742.13** | **2.74** | **0.006** |
| **Disturbance 70** | **0.38 ± 0.05** | **742.06** | **7.13** | **< 0.001** |
| **Disturbance 90** | **-0.14 ± 0.05** | **742.11** | **-2.70** | **0.007** |
| **Low subsidy flow x Dist. 50** | **0.28 ± 0.07** | **742.06** | **3.78** | **< 0.001** |
| Low subsidy flow x Dist. 70 | 0.08 ± 0.07 | 742.11 | 1.12 | 0.265 |
| **Low subsidy flow x Dist. 90** | **0.15 ± 0.07** | **742.09** | **2.07** | **0.039** |
| High subsidy flow x Dist. 50 | 0.13 ± 0.07 | 742.15 | 1.68 | 0.094 |
| High subsidy flow x Dist. 70 | -0.13 ± 0.07 | 742.07 | -1.79 | 0.074 |
| High subsidy flow x Dist. 90 | 0.13 ± 0.08 | 742.12 | 1.78 | 0.076 |

***(b) Local high-resource ecosystems*** (Shapiro-Wilk normality test: W = 0.99, p < 0.001)

|  | Estimate ± S.E. | df | t-value | p-value |
| --- | --- | --- | --- | --- |
| **Low resource metaflow** | **-0.20 ± 0.05** | **727.21** | **-3.77** | **< 0.001** |
| **High resource metaflow** | **-0.35 ± 0.05** | **727.55** | **-6.60** | **< 0.001** |
| **Disturbance 50** | **-0.73 ± 0.05** | **727.11** | **-14.02** | **< 0.001** |
| **Disturbance 70** | **-0.89 ± 0.05** | **727.66** | **-16.88** | **< 0.001** |
| **Disturbance 90** | **-1.08 ± 0.05** | **727.44** | **-20.61** | **< 0.001** |
| Low subsidy flow x Dist. 50 | 0.02 ± 0.07 | 727.22 | 0.23 | 0.818 |
| **Low subsidy flow x Dist. 70** | **0.23 ± 0.08** | **727.59** | **3.07** | **0.002** |
| **Low subsidy flow x Dist. 90** | **0.15 ± 0.07** | **727.38** | **2.04** | **0.042** |
| **High subsidy flow x Dist. 50** | **0.37 ± 0.07** | **727.68** | **4.95** | **< 0.001** |
| **High subsidy flow x Dist. 70** | **0.34 ± 0.07** | **727.48** | **4.56** | **< 0.001** |
| **High subsidy flow x Dist. 90** | **0.27 ± 0.07** | **727.56** | **3.57** | **< 0.001** |

**Table S10-A**: Statistical summary output from repeated-measures linear mixed effect model using size class evenness as a response variable and disturbance intensity and subsidy flow as fixed predicted variables, time as a repeated measure and replicate as a random effect. We report regression estimate ± standard error (S.E.), degrees of freedom (df), value of the t-statistic and p-value. p-values below 0.05 are indicated in bold and represent significant effects. Statistical analysis is performed for the local low- and high-resource ecosystems separately, but including the isolated ecosystems as a control in both analyses. The results using the patch 2 experimental units are reported in Supplementary Table S10-B.

***(a) Local low-resource ecosystems*** (Shapiro-Wilk normality test: W = 0.97, p < 0.001)

|  | Estimate ± S.E. | df | t-value | p-value |
| --- | --- | --- | --- | --- |
| **Low resource metaflow** | **-0.07 ± 0.03** | **737.06** | **-2.52** | **0.012** |
| High resource metaflow | -0.04 ± 0.03 | 737.06 | -1.58 | 0.115 |
| **Disturbance 50** | **0.09 ± 0.03** | **737.20** | **3.61** | **< 0.001** |
| **Disturbance 70** | **0.23 ± 0.03** | **737.56** | **8.82** | **< 0.001** |
| Disturbance 90 | -0.01 ± 0.03 | 737.56 | -0.28 | 0.782 |
| **Low subsidy flow x Dist. 50** | **0.08 ± 0.04** | **737.10** | **2.10** | **0.036** |
| Low subsidy flow x Dist. 70 | 0.07 ± 0.04 | 737.12 | 1.94 | 0.053 |
| Low subsidy flow x Dist. 90 | 0.07 ± 0.04 | 737.31 | 1.77 | 0.077 |
| High subsidy flow x Dist. 50 | 0.05 ± 0.04 | 737.23 | 1.28 | 0.201 |
| **High subsidy flow x Dist. 70** | **-0.08 ± 0.04** | **737.12** | **-2.14** | **0.033** |
| High subsidy flow x Dist. 90 | 0.07 ± 0.04 | 737.58 | 1.94 | 0.052 |

***(b) Local high-resource ecosystems*** (Shapiro-Wilk normality test: W = 1.00, p = 0.017)

|  | Estimate ± S.E. | df | t-value | p-value |
| --- | --- | --- | --- | --- |
| **Low resource metaflow** | **-0.08 ± 0.02** | **732.05** | **-3.07** | **0.002** |
| **High resource metaflow** | **-0.14 ± 0.02** | **732.00** | **-5.70** | **< 0.001** |
| **Disturbance 50** | **-0.27 ± 0.02** | **731.95** | **-10.98** | **< 0.001** |
| **Disturbance 70** | **-0.36 ± 0.02** | **732.31** | **-14.30** | **< 0.001** |
| **Disturbance 90** | **-0.43 ± 0.02** | **732.17** | **-17.28** | **< 0.001** |
| Low subsidy flow x Dist. 50 | -0.03 ± 0.04 | 732.05 | -0.71 | 0.477 |
| **Low subsidy flow x Dist. 70** | **0.10 ± 0.04** | **732.27** | **2.77** | **0.006** |
| Low subsidy flow x Dist. 90 | 0.07 ± 0.04 | 732.18 | 1.86 | 0.063 |
| **High subsidy flow x Dist. 50** | **0.09 ± 0.03** | **732.00** | **2.64** | **0.009** |
| **High subsidy flow x Dist. 70** | **0.11 ± 0.04** | **732.37** | **3.06** | **0.002** |
| **High subsidy flow x Dist. 90** | **0.10 ± 0.04** | **732.23** | **2.96** | **0.003** |

**Table S10-B**: Statistical summary output from repeated-measures linear mixed effect model using size class evenness as a response variable and disturbance intensity and subsidy flow as fixed predicted variables, time as a repeated measure and replicate as a random effect using the patch 2 experimental units. We report regression estimate ± standard error (S.E.), degrees of freedom (df), value of the t-statistic and p-value. p-values below 0.05 are indicated in bold and represent significant effects. Statistical analysis is performed for the local low- and high-resource ecosystems separately, but including the isolated ecosystems as a control in both analyses.

***(a) Local low-resource ecosystems*** (Shapiro-Wilk normality test: W = 0.98, p < 0.001)

|  | Estimate ± S.E. | df | t-value | p-value |
| --- | --- | --- | --- | --- |
| Low resource metaflow | -0.04 ± 0.03 | 733.01 | -1.43 | 0.153 |
| High resource metaflow | -0.04 ± 0.03 | 733.05 | -1.58 | 0.114 |
| **Disturbance 50** | **0.09 ± 0.03** | **733.16** | **3.58** | **< 0.001** |
| **Disturbance 70** | **0.23 ± 0.03** | **733.08** | **8.75** | **< 0.001** |
| Disturbance 90 | -0.01 ± 0.03 | 733.49 | -0.24 | 0.808 |
| **Low subsidy flow x Dist. 50** | **0.14 ± 0.04** | **733.08** | **3.82** | **< 0.001** |
| Low subsidy flow x Dist. 70 | 0.05 ± 0.04 | 733.14 | 1.25 | 0.214 |
| Low subsidy flow x Dist. 90 | 0.06 ± 0.04 | 733.37 | 1.65 | 0.099 |
| High subsidy flow x Dist. 50 | 0.05 ± 0.04 | 733.19 | 1.30 | 0.194 |
| **High subsidy flow x Dist. 70** | **-0.08 ± 0.04** | **733.10** | **-2.11** | **0.035** |
| High subsidy flow x Dist. 90 | 0.07 ± 0.04 | 733.51 | 1.93 | 0.054 |

***(b) Local high-resource ecosystems*** (Shapiro-Wilk normality test: W = 1.00, p = 0.224)

|  | Estimate ± S.E. | df | t-value | p-value |
| --- | --- | --- | --- | --- |
| **Low resource metaflow** | **-0.08 ± 0.02** | **727.18** | **-3.12** | **0.002** |
| **High resource metaflow** | **-0.11 ± 0.02** | **727.41** | **-4.64** | **< 0.001** |
| **Disturbance 50** | **-0.27 ± 0.02** | **727.09** | **-11.12** | **< 0.001** |
| **Disturbance 70** | **-0.36 ± 0.02** | **727.43** | **-14.45** | **< 0.001** |
| **Disturbance 90** | **-0.43 ± 0.02** | **727.30** | **-17.49** | **< 0.001** |
| Low subsidy flow x Dist. 50 | -0.02 ± 0.03 | 727.18 | -0.71 | 0.477 |
| **Low subsidy flow x Dist. 70** | **0.10 ± 0.04** | **727.39** | **2.80** | **0.005** |
| Low subsidy flow x Dist. 90 | 0.07 ± 0.04 | 727.30 | 1.90 | 0.058 |
| **High subsidy flow x Dist. 50** | **0.12 ± 0.04** | **727.51** | **3.29** | **0.001** |
| **High subsidy flow x Dist. 70** | **0.12 ± 0.03** | **727.34** | **3.49** | **< 0.001** |
| **High subsidy flow x Dist. 90** | **0.08 ± 0.04** | **727.38** | **2.37** | **0.018** |

**Table S11-A**: Log-response ratio of size class diversity comparing each time point to the first time point for (a) isolated ecosystems, (b) ecosystems connected to a low-resource ecosystem and (c) ecosystems connected to a high-resource ecosystem. The first value gives the log-response ratio estimate and values between brackets indicate the 95% confidence interval. Values in bold reflect where the 95% confidence interval does not overlap with zero. The results using the patch 2 experimental units are reported in Supplementary Table S11-B.

***(a) Unconnected meta-ecosystems***

*Low resource ecosystem*

| -0.3398(-0.7443,0.0646) | -0.2851(-0.6273,0.0571) | -0.1348(-0.3039,0.0343) | **-0.3074(-0.572,-0.0427)** |
| --- | --- | --- | --- |
| **-0.513(-1.0062,-0.0199)** | **-0.4488(-0.7565,-0.1412)** | **-0.3683(-0.568,-0.1685)** | **-0.9066(-1.129,-0.6841)** |
| -0.511(-1.0324,0.0103) | **-0.3399(-0.6276,-0.0522)** | -0.3958(-0.8224,0.0309) | **-0.4963(-0.8133,-0.1792)** |
| **-0.666(-0.8428,-0.4891)** | **-0.7522(-1.2055,-0.2989)** | -0.189(-0.4692,0.0911) | **-0.4552(-0.7196,-0.1907)** |
| **-0.5242(-0.7884,-0.26)** | **-0.4698(-0.8711,-0.0684)** | -0.0163(-0.2401,0.2075) | **-1.2983(-1.7875,-0.809)** |
| **-0.5517(-0.767,-0.3364)** | -0.2652(-0.695,0.1646) | 0.0866(-0.1241,0.2973) | **-1.0176(-1.4269,-0.6083)** |
| **-0.4276(-0.7975,-0.0577)** | -0.2662(-0.6258,0.0935) | 0.0313(-0.2446,0.3072) | **-1.2406(-1.6543,-0.8269)** |
| **-0.5685(-0.9675,-0.1696)** | -0.0529(-0.2086,0.1028) | **0.2023(0.0011,0.4036)** | -1.2476(-3.0479,0.5527) |
| **-0.6941(-1.2056,-0.1827)** | -0.1785(-0.4197,0.0626) | **0.1936(0.0213,0.3658)** | -1.0574(-2.2374,0.1225) |
| **-0.7839(-1.3343,-0.2335)** | -0.0386(-0.3115,0.2342) | **0.3358(0.1757,0.4959)** | **-1.202(-2.0576,-0.3463)** |

*High resource ecosystem*

| **0.3293(0.2087,0.4499)** | **0.2442(0.0244,0.4641)** | 0.1634(-0.0899,0.4167) | **-0.0128(-0.2747,0.2491)** |
| --- | --- | --- | --- |
| **0.5344(0.4057,0.6631)** | **0.2916(0.1155,0.4676)** | 0.0548(-0.1822,0.2917) | **-0.2982(-0.4987,-0.0978)** |
| **0.6409(0.5273,0.7545)** | -0.0175(-0.3762,0.3412) | **-0.2938(-0.5129,-0.0747)** | **-0.5091(-0.7639,-0.2544)** |
| **0.6607(0.5365,0.7849)** | -0.1096(-0.5172,0.2979) | **-0.4418(-0.8754,-0.0083)** | **-0.5556(-0.7674,-0.3437)** |
| **0.6107(0.4677,0.7537)** | -0.2053(-0.5891,0.1786) | **-0.5095(-0.7633,-0.2557)** | **-0.8025(-1.1579,-0.447)** |
| **0.6145(0.4503,0.7787)** | -0.2185(-0.5214,0.0844) | **-0.5493(-0.7728,-0.3258)** | **-0.9169(-1.302,-0.5318)** |
| **0.5458(0.3117,0.7798)** | -0.2867(-0.5926,0.0192) | **-0.495(-0.8626,-0.1275)** | **-0.8464(-1.33,-0.3629)** |
| **0.5506(0.3359,0.7654)** | -0.4088(-0.8286,0.0111) | **-0.5754(-0.9076,-0.2433)** | **-1.0427(-1.2464,-0.839)** |
| **0.5327(0.3321,0.7333)** | -0.1935(-0.5572,0.1701) | **-0.5663(-1.0735,-0.0591)** | **-1.3841(-1.7825,-0.9858)** |
| **0.4476(0.2096,0.6857)** | **-0.3373(-0.6519,-0.0228)** | **-0.4906(-0.781,-0.2002)** | **-1.2922(-1.656,-0.9283)** |

***(b) Low-resource connected meta-ecosystems***

*Low resource ecosystem*

| -0.1956(-0.4786,0.0874) | **-0.1497(-0.435,0.1356)** | 0.003(-0.2731,0.279) | -0.2367(-0.5716,0.0982) |
| --- | --- | --- | --- |
| **-0.6189(-1.078,-0.1597)** | **-0.5542(-0.9403,-0.1681)** | **-0.2867(-0.5692,-0.0042)** | **-0.6971(-0.9904,-0.4038)** |
| **-0.8435(-1.3085,-0.3786)** | **-0.723(-1.386,-0.06)** | **-0.3049(-0.578,-0.0318)** | **-0.5175(-0.7981,-0.2368)** |
| **-1.1287(-1.5635,-0.694)** | **-0.6696(-1.1734,-0.1658)** | -0.1758(-0.4507,0.0991) | **-0.621(-0.9868,-0.2553)** |
| **-1.1453(-1.3963,-0.8943)** | -0.4846(-1.0291,0.06) | -0.0803(-0.3919,0.2312) | **-1.4031(-1.676,-1.1303)** |
| **-0.9289(-1.1031,-0.7547)** | -0.1946(-0.6634,0.2743) | -0.0446(-0.3267,0.2376) | **-1.3906(-1.9912,-0.7901)** |
| **-0.8793(-1.1534,-0.6051)** | -0.1453(-0.6109,0.3202) | **0.3411(0.0929,0.5893)** | **-1.5548(-1.999,-1.1105)** |
| **-1.0663(-1.3058,-0.8268)** | -0.0469(-0.5098,0.416) | **0.3061(0.0822,0.53)** | **-1.1333(-1.5451,-0.7215)** |
| **-1.3522(-1.643,-1.0615)** | -0.0251(-0.3964,0.3461) | **0.173(0.0108,0.3352)** | **-1.7256(-2.6606,-0.7906)** |
| **-1.5272(-1.6991,-1.3553)** | -0.0127(-0.2598,0.2345) | **0.1337(-0.0162,0.2837)** | **-0.6682(-1.2175,-0.1189)** |

*High resource ecosystem*

| **0.2064(0.0422,0.3705)** | -0.0119(-0.3209,0.2972) | 0.0588(-0.1159,0.2335) | -0.2144(-0.4926,0.0637) |
| --- | --- | --- | --- |
| **0.3524(0.1141,0.5908)** | -0.0622(-0.3782,0.2537) | 0.0028(-0.2127,0.2183) | **-0.4361(-0.6515,-0.2207)** |
| 0.2762(-0.085,0.6373) | -0.29(-0.5912,0.0112) | -0.1(-0.3244,0.1244) | **-0.6739(-1.0079,-0.34)** |
| 0.2364(-0.0767,0.5495) | **-0.5145(-0.7684,-0.2606)** | **-0.3237(-0.5975,-0.0498)** | **-0.8304(-1.0195,-0.6413)** |
| **0.2639(0.0113,0.5164)** | **-0.6016(-0.9979,-0.2053)** | **-0.554(-0.8927,-0.2153)** | **-1.139(-1.6357,-0.6424)** |
| 0.2445(-0.0328,0.5218) | **-0.75(-1.0187,-0.4814)** | **-0.7726(-0.9904,-0.5548)** | **-1.312(-1.8952,-0.7287)** |
| **0.2743(0.0011,0.5475)** | **-0.7985(-1.1667,-0.4302)** | **-0.8063(-1.2515,-0.361)** | **-1.3094(-1.7638,-0.855)** |
| **0.3275(0.0957,0.5594)** | **-1.0039(-1.515,-0.4928)** | **-0.8595(-1.5059,-0.2131)** | **-1.4135(-1.6017,-1.2253)** |
| **0.3775(0.2061,0.5489)** | **-0.7322(-1.1497,-0.3148)** | **-0.7105(-1.1363,-0.2846)** | **-1.3122(-1.6538,-0.9706)** |
| 0.2357(-0.0042,0.4755) | **-0.6873(-0.8746,-0.5)** | **-0.6579(-0.8969,-0.419)** | **-1.4118(-1.7386,-1.085)** |

***(c) High-resource connected meta-ecosystems***

*Low resource ecosystem*

| -0.1635(-0.4719,0.145) | -0.029(-0.2666,0.2085) | **-0.1452(-0.2484,-0.0419)** | **-0.3837(-0.6464,-0.121)** |
| --- | --- | --- | --- |
| -0.3191(-0.7801,0.1418) | -0.1838(-0.5511,0.1835) | **-0.6335(-0.7439,-0.5232)** | **-1.0526(-1.5697,-0.5355)** |
| -0.4572(-0.9463,0.0319) | -0.2144(-0.5648,0.1361) | **-0.3226(-0.5832,-0.062)** | **-0.8912(-1.4067,-0.3756)** |
| **-0.8178(-1.2044,-0.4311)** | -0.3504(-0.8068,0.1061) | **-0.3504(-0.5587,-0.1421)** | **-0.5913(-0.887,-0.2957)** |
| **-0.7681(-1.3529,-0.1834)** | -0.3229(-0.78,0.1342) | **-0.4714(-0.79,-0.1529)** | **-0.8604(-1.2991,-0.4218)** |
| **-0.643(-1.2276,-0.0584)** | -0.2106(-0.5906,0.1695) | **-0.3123(-0.4522,-0.1724)** | **-1.1621(-1.576,-0.7483)** |
| -0.6897(-1.4281,0.0486) | -0.0847(-0.4611,0.2916) | 0.0409(-0.1895,0.2712) | **-0.7013(-1.0505,-0.3521)** |
| **-0.9153(-1.5118,-0.3187)** | -0.1679(-0.6899,0.3541) | -0.0076(-0.1756,0.1605) | **-0.5122(-0.7965,-0.2279)** |
| **-1.3512(-1.8813,-0.821)** | -0.1709(-0.5391,0.1973) | -0.3664(-0.6534,-0.0793) | -0.9879(-2.2522,0.2764) |
| **-1.6117(-1.904,-1.3193)** | **-0.6191(-1.0106,-0.2275)** | -0.1715(-0.5281,0.1851) | -0.3967(-1.0387,0.2453) |

*High resource ecosystem*

| **0.3045(0.1038,0.5051)** | 0.1891(0.0498,0.3284) | 0.0319(-0.231,0.2947) | -0.1351(-0.2492,-0.0211) |
| --- | --- | --- | --- |
| **0.444(0.2636,0.6244)** | 0.1505(-0.0938,0.3949) | -0.1(-0.3805,0.1804) | -0.2805(-0.5724,0.0113) |
| **0.4136(0.195,0.6321)** | 0.1334(-0.1593,0.4261) | -0.2423(-0.5227,0.0381) | **-0.499(-0.7706,-0.2273)** |
| **0.2414(0.0055,0.4772)** | 0.1129(-0.1621,0.388) | -0.4567(-0.9986,0.0852) | **-1.0652(-1.2822,-0.8482)** |
| 0.2679(-0.0739,0.6097) | -0.1235(-0.39,0.143) | **-0.5295(-0.9983,-0.0607)** | **-1.0936(-1.6478,-0.5395)** |
| 0.2576(-0.0203,0.5355) | **-0.3632(-0.5473,-0.1791)** | **-0.6868(-1.1591,-0.2145)** | **-1.3928(-1.7875,-0.9981)** |
| 0.1852(-0.0976,0.468) | **-0.5556(-0.8477,-0.2636)** | **-0.8295(-1.0767,-0.5823)** | **-1.2737(-1.6871,-0.8604)** |
| 0.1877(-0.0725,0.448) | **-0.5821(-0.9846,-0.1796)** | **-0.7836(-1.3295,-0.2378)** | **-1.2027(-1.5542,-0.8511)** |
| 0.1593(-0.0508,0.3695) | **-0.5579(-0.9698,-0.1461)** | **-0.7191(-1.1707,-0.2674)** | **-1.2564(-1.7767,-0.7362)** |
| 0.1478(-0.0523,0.3478) | **-0.5139(-0.9338,-0.0941)** | **-0.6984(-1.0225,-0.3742)** | **-1.4357(-1.7711,-1.1003)** |

**Table S11-B**: Log-response ratio of size class diversity comparing each time point to the first time point for (a) isolated ecosystems, (b) ecosystems connected to a low-resource ecosystem and (c) ecosystems connected to a high-resource ecosystem using the patch 2 experimental units. The first value gives the log-response ratio estimate and values between brackets indicate the 95% confidence interval. Values in bold reflect where the 95% confidence interval does not overlap with zero.

***(a) Low-resource connected meta-ecosystems***

*Low resource ecosystem*

| -0.2344(-0.4865,0.0177) | 0.0354(-0.2683,0.3392) | -0.2952(-0.5619,-0.0284) | -0.044(-0.4292,0.3413) |
| --- | --- | --- | --- |
| -0.4453(-0.816,-0.0746) | -0.3018(-0.648,0.0445) | -0.3064(-0.4474,-0.1654) | -0.7058(-1.2721,-0.1395) |
| -0.7615(-1.0341,-0.489) | -0.452(-0.8965,-0.0075) | -0.2488(-0.6781,0.1806) | -0.5459(-0.8445,-0.2473) |
| -0.847(-1.0346,-0.6595) | -0.5233(-0.9668,-0.0799) | -0.3305(-0.5969,-0.0642) | -0.9026(-1.1299,-0.6753) |
| -0.9935(-1.1384,-0.8486) | -0.1624(-0.6553,0.3304) | -0.2007(-0.5358,0.1344) | -2.0533(-2.2979,-1.8087) |
| -1.0626(-1.3291,-0.7961) | -0.0062(-0.4312,0.4188) | 0.0075(-0.2994,0.3144) | -0.994(-1.5771,-0.4109) |
| -0.8881(-1.1645,-0.6118) | 0.0835(-0.1389,0.3059) | 0.2093(-0.0599,0.4785) | -1.3823(-2.0407,-0.7239) |
| -0.9669(-1.412,-0.5219) | 0.1334(-0.1168,0.3836) | 0.2115(-0.0112,0.4341) | -0.5656(-0.7869,-0.3443) |
| -0.9962(-1.5197,-0.4727) | 0.1596(-0.1004,0.4195) | 0.3297(0.2155,0.4439) | -0.7736(-1.4209,-0.1264) |
| -0.8122(-1.4105,-0.214) | 0.032(-0.3315,0.3954) | 0.1283(-0.052,0.3086) | -0.645(-1.0621,-0.2278) |

***(b) High-resource connected meta-ecosystems***

*High resource ecosystem*

| 0.2709(0.0774,0.4644) | 0.0928(-0.1598,0.3454) | 0.0572(-0.1038,0.2182) | -0.1662(-0.2945,-0.038) |
| --- | --- | --- | --- |
| 0.3258(0.1818,0.4698) | 0.1535(-0.0195,0.3265) | -0.0706(-0.2573,0.1162) | -0.6269(-0.7889,-0.465) |
| 0.3457(0.1976,0.4937) | 0.0201(-0.1521,0.1923) | -0.3574(-0.5136,-0.2013) | -1.0927(-1.3652,-0.8202) |
| 0.2491(0.0815,0.4168) | -0.0595(-0.2745,0.1554) | -0.3752(-0.6657,-0.0847) | -1.2938(-1.7313,-0.8562) |
| 0.1772(-0.0793,0.4336) | -0.3771(-0.6079,-0.1464) | -0.4349(-0.7677,-0.1021) | -1.4095(-1.6745,-1.1444) |
| 0.1762(-0.0921,0.4446) | -0.1201(-0.4108,0.1706) | -0.7309(-0.9363,-0.5254) | -1.2473(-1.5197,-0.9748) |
| 0.0654(-0.2374,0.3682) | -0.296(-0.6454,0.0534) | -0.8693(-1.1387,-0.6) | -1.3276(-1.7452,-0.91) |
| -0.0244(-0.3176,0.2689) | -0.5881(-0.8717,-0.3044) | -0.9665(-1.299,-0.6339) | -1.1633(-1.5166,-0.8101) |
| -0.039(-0.1952,0.1173) | -0.7524(-1.1549,-0.35) | -0.8179(-1.1508,-0.485) | -1.1245(-1.4368,-0.8122) |
| -0.1459(-0.3116,0.0198) | -0.4634(-1.0021,0.0753) | -0.8094(-1.112,-0.5067) | -1.1901(-1.6021,-0.7782) |

**Table S12-A**: Log-response ratio of size class evenness comparing each time point to the first time point for (a) isolated ecosystems, (b) ecosystems connected to a low-resource ecosystem and (c) ecosystems connected to a high-resource ecosystem. The first value gives the log-response ratio estimate and values between brackets indicate the 95% confidence interval. Values in bold reflect where the 95% confidence interval does not overlap with zero. The results using the patch 2 experimental units are reported in Supplementary Table S12-B.

***(a) Unconnected meta-ecosystems***

*Low resource ecosystem*

| **-0.3671(-0.7157,-0.0185)** | -0.1897(-0.4834,0.1039) | -0.0159(-0.1729,0.141) | -0.1555(-0.4002,0.0893) |
| --- | --- | --- | --- |
| **-0.5237(-0.9943,-0.053)** | -0.3142(-0.6672,0.0387) | **-0.1952(-0.3409,-0.0495)** | **-0.6647(-0.9383,-0.391)** |
| **-0.4222(-0.9926,0.1482)** | -0.1704(-0.4392,0.0983) | -0.2125(-0.6049,0.1798) | **-0.2849(-0.5651,-0.0047)** |
| **-0.5729(-0.8046,-0.3411)** | **-0.6142(-1.0413,-0.187)** | 0.0158(-0.2862,0.3179) | **-0.1795(-0.4189,0.0599)** |
| **-0.4039(-0.7297,-0.0781)** | -0.3234(-0.6965,0.0498) | 0.0785(-0.1012,0.2581) | **-0.8845(-1.2716,-0.4974)** |
| **-0.4631(-0.6895,-0.2367)** | -0.0688(-0.4977,0.3601) | 0.2245(-0.0417,0.4906) | **-0.6577(-0.997,-0.3183)** |
| **-0.3562(-0.709,-0.0035)** | -0.1149(-0.4552,0.2254) | 0.2232(-0.0453,0.4916) | **-0.8794(-1.2889,-0.4699)** |
| **-0.4841(-0.8589,-0.1094)** | 0.0903(-0.0503,0.2309) | **0.4289(0.2637,0.5941)** | -0.214(-1.4002,0.9722) |
| **-0.5858(-1.0386,-0.133)** | 0.0522(-0.1898,0.2941) | **0.4761(0.3333,0.6189)** | -0.5497(-1.3571,0.2577) |
| **-0.6598(-1.2217,-0.0979)** | 0.0836(-0.1901,0.3573) | **0.5351(0.3965,0.6737)** | -0.6666(-1.4596,0.1264) |

*High resource ecosystem*

| **0.2641(0.1409,0.3873)** | 0.2066(-0.0025,0.4157) | 0.1821(-0.0404,0.4046) | 0.0588(-0.1751,0.2926) |
| --- | --- | --- | --- |
| **0.4671(0.3542,0.58)** | 0.3283(0.1713,0.4854) | 1e-04(-0.2237,0.2238) | -0.1741(-0.3683,0.0201) |
| **0.5429(0.4345,0.6513)** | -0.0039(-0.3438,0.336) | -0.2444(-0.4718,-0.017) | -0.3628(-0.6286,-0.097) |
| **0.5857(0.4728,0.6986)** | -0.0429(-0.4557,0.3699) | -0.3765(-0.7466,-0.0065) | -0.4481(-0.7777,-0.1185) |
| **0.5408(0.3947,0.6869)** | -0.1767(-0.5263,0.1728) | -0.4784(-0.7136,-0.2433) | -0.7034(-1.0342,-0.3725) |
| **0.5366(0.3718,0.7014)** | -0.1462(-0.4382,0.1458) | -0.4595(-0.7292,-0.1897) | -0.8649(-1.2017,-0.5281) |
| **0.5033(0.2614,0.7451)** | -0.1778(-0.5612,0.2056) | -0.4815(-0.8929,-0.0701) | -0.7828(-1.2149,-0.3508) |
| **0.5675(0.3321,0.8029)** | -0.2744(-0.7128,0.164) | -0.507(-0.9383,-0.0757) | -0.9135(-1.0797,-0.7472) |
| **0.543(0.3113,0.7746)** | -0.0475(-0.4346,0.3396) | -0.4795(-1.0312,0.0721) | -1.2399(-1.615,-0.8649) |
| **0.4435(0.2699,0.617)** | -0.1834(-0.5437,0.1768) | -0.4261(-0.819,-0.0332) | -1.1043(-1.4726,-0.736) |

***(b) Low-resource connected meta-ecosystems***

*Low resource ecosystem*

| -0.1769(-0.5086,0.1549) | -0.1557(-0.3914,0.08) | 0.0951(-0.096,0.2861) | -0.0883(-0.3924,0.2158) |
| --- | --- | --- | --- |
| **-0.5458(-0.98,-0.1115)** | **-0.4632(-0.8429,-0.0834)** | -0.1049(-0.3843,0.1745) | **-0.4708(-0.7773,-0.1643)** |
| **-0.7062(-1.1887,-0.2236)** | -0.479(-1.1403,0.1822) | -0.1699(-0.4779,0.1382) | -0.2277(-0.5026,0.0472) |
| **-0.9551(-1.3765,-0.5337)** | -0.4528(-0.976,0.0704) | -0.0572(-0.3533,0.2389) | -0.2912(-0.6063,0.024) |
| **-0.9835(-1.252,-0.7151)** | -0.3003(-0.8591,0.2585) | 0.0739(-0.2163,0.3641) | **-1.0148(-1.2719,-0.7577)** |
| **-0.7437(-0.9267,-0.5608)** | -0.0128(-0.5359,0.5102) | 0.1255(-0.0891,0.3402) | **-0.8698(-1.6164,-0.1231)** |
| **-0.7144(-0.9719,-0.457)** | 0.0033(-0.4422,0.4487) | **0.4507(0.2464,0.655)** | **-1.2443(-1.5404,-0.9481)** |
| **-0.9944(-1.2565,-0.7322)** | 0.1945(-0.2665,0.6554) | **0.459(0.2812,0.6368)** | **-0.6009(-0.8793,-0.3224)** |
| **-1.1917(-1.4862,-0.8972)** | 0.218(-0.1947,0.6308) | **0.4276(0.27,0.5853)** | **-0.746(-1.2456,-0.2464)** |
| **-1.3379(-1.4663,-1.2096)** | 0.2162(-0.0503,0.4826) | **0.4525(0.2955,0.6096)** | -0.2898(-0.7723,0.1928) |

*High resource ecosystem*

| **0.2047(0.0239,0.3854)** | 0.0011(-0.3045,0.3068) | 0.0878(-0.064,0.2396) | -0.0825(-0.2944,0.1294) |
| --- | --- | --- | --- |
| **0.3239(0.0734,0.5744)** | -0.0717(-0.4067,0.2632) | 0.0909(-0.0902,0.2721) | -0.2215(-0.5271,0.0841) |
| 0.2653(-0.0942,0.6249) | -0.2209(-0.5508,0.109) | -0.048(-0.2489,0.1529) | -0.4521(-0.7682,-0.136) |
| 0.1891(-0.1171,0.4953) | -0.4652(-0.7398,-0.1907) | -0.187(-0.4645,0.0906) | -0.5664(-0.7592,-0.3736) |
| **0.2676(0.0022,0.533)** | -0.5645(-0.9477,-0.1814) | -0.4583(-0.7424,-0.1742) | -0.9217(-1.4499,-0.3935) |
| 0.2436(-0.0397,0.5269) | -0.6481(-0.9416,-0.3546) | -0.6472(-0.838,-0.4565) | -1.1766(-1.7414,-0.6118) |
| **0.3298(0.0128,0.6469)** | -0.6793(-1.0582,-0.3004) | -0.6634(-1.1047,-0.2221) | -1.1237(-1.6536,-0.5939) |
| **0.3655(0.1213,0.6097)** | -0.9164(-1.496,-0.3368) | -0.7351(-1.3745,-0.0957) | -1.1849(-1.3895,-0.9802) |
| **0.3986(0.1744,0.6228)** | -0.6175(-1.0483,-0.1867) | -0.6033(-1.0473,-0.1592) | -1.0603(-1.375,-0.7456) |
| 0.2972(-0.0039,0.5982) | -0.5926(-0.8666,-0.3186) | -0.5107(-0.7897,-0.2317) | -1.1727(-1.4264,-0.919) |

***(c) High-resource connected meta-ecosystems***

*Low resource ecosystem*

| -0.1664(-0.4359,0.103) | -0.0267(-0.2355,0.1821) | **-0.093(-0.185,-0.001)** | **-0.2383(-0.4499,-0.0268)** |
| --- | --- | --- | --- |
| -0.2595(-0.6786,0.1596) | -0.1262(-0.4478,0.1955) | **-0.4542(-0.5937,-0.3147)** | **-0.863(-1.3821,-0.3439)** |
| -0.4105(-0.8492,0.0282) | -0.0268(-0.3645,0.3109) | -0.148(-0.4155,0.1196) | **-0.6143(-1.0847,-0.1439)** |
| **-0.7516(-1.1113,-0.392)** | -0.3166(-0.7244,0.0912) | -0.2359(-0.428,-0.0438) | **-0.3687(-0.6248,-0.1126)** |
| **-0.7153(-1.2137,-0.2169)** | -0.2641(-0.6759,0.1476) | -0.2997(-0.6168,0.0174) | **-0.4011(-0.5183,-0.284)** |
| **-0.6322(-1.219,-0.0454)** | -0.1453(-0.5108,0.2202) | **-0.1657(-0.2975,-0.0339)** | **-0.7546(-1.0535,-0.4557)** |
| -0.6274(-1.3367,0.0819) | 0.0442(-0.3269,0.4154) | 0.1702(-0.0329,0.3733) | **-0.4459(-0.7801,-0.1117)** |
| **-0.8049(-1.402,-0.2079)** | -0.0279(-0.4511,0.3952) | 0.1937(0.0014,0.3861) | -0.1509(-0.4169,0.1152) |
| **-1.1987(-1.7293,-0.6681)** | 0.0449(-0.3594,0.4493) | -0.1035(-0.3623,0.1552) | -0.0111(-0.4339,0.4116) |
| **-1.5053(-1.827,-1.1835)** | **-0.4235(-0.8162,-0.0308)** | 0.0986(-0.2398,0.4371) | 0.0955(-0.317,0.5081) |

*High resource ecosystem*

| **0.2952(0.084,0.5064)** | 0.1617(0.0257,0.2978) | 0.0323(-0.2156,0.2803) | 0.0155(-0.1273,0.1583) |
| --- | --- | --- | --- |
| **0.431(0.2469,0.615)** | 0.1441(-0.0798,0.3679) | 0.0204(-0.2081,0.2489) | -0.1671(-0.4299,0.0957) |
| **0.3947(0.1714,0.6181)** | 0.1582(-0.149,0.4654) | -0.1325(-0.4121,0.147) | -0.3183(-0.5201,-0.1164) |
| 0.262(-0.0026,0.5267) | 0.1345(-0.1357,0.4048) | -0.4143(-0.9199,0.0913) | -0.9221(-1.1398,-0.7043) |
| 0.2991(-0.0627,0.6609) | -0.1087(-0.4037,0.1863) | -0.3905(-0.8588,0.0778) | -0.9688(-1.5275,-0.4101) |
| **0.3005(0.0013,0.5997)** | -0.2306(-0.4413,-0.0199) | -0.5684(-1.0307,-0.1061) | -1.2429(-1.6037,-0.882) |
| 0.23(-0.0467,0.5066) | -0.5781(-0.8325,-0.3237) | -0.6689(-0.9458,-0.3919) | -1.1409(-1.4507,-0.8312) |
| **0.2666(0.0034,0.5298)** | -0.5275(-0.8926,-0.1623) | -0.6296(-1.2816,0.0224) | -1.0321(-1.4288,-0.6354) |
| **0.2292(0.0084,0.45)** | -0.47(-0.8933,-0.0468) | -0.5955(-1.1103,-0.0808) | -0.9948(-1.5194,-0.4702) |
| **0.2415(0.0015,0.4814)** | -0.3471(-0.8727,0.1786) | -0.6081(-0.9844,-0.2319) | -1.2204(-1.5984,-0.8424) |

**Table S12-B**: Log-response ratio of size class evenness comparing each time point to the first time point for (a) isolated ecosystems, (b) ecosystems connected to a low-resource ecosystem and (c) ecosystems connected to a high-resource ecosystem using the patch 2 experimental units. The first value gives the log-response ratio estimate and values between brackets indicate the 95% confidence interval. Values in bold reflect where the 95% confidence interval does not overlap with zero.

***(a) Low-resource connected meta-ecosystems***

*Low resource ecosystem*

| -0.2389(-0.4615,-0.0162) | 0.0422(-0.2506,0.335) | -0.1374(-0.4225,0.1478) | 0.1213(-0.2694,0.512) |
| --- | --- | --- | --- |
| -0.3843(-0.7369,-0.0316) | -0.1197(-0.4737,0.2342) | -0.1526(-0.2593,-0.0459) | -0.4555(-0.9671,0.0561) |
| -0.5984(-0.8583,-0.3384) | -0.271(-0.665,0.123) | -0.0276(-0.3715,0.3163) | -0.3271(-0.5102,-0.144) |
| -0.7239(-0.9238,-0.5239) | -0.3466(-0.7782,0.085) | -0.0919(-0.3248,0.141) | -0.581(-0.7894,-0.3726) |
| -0.8072(-0.9428,-0.6717) | -0.0411(-0.493,0.4109) | 0.1029(-0.2223,0.4281) | -1.3959(-1.7262,-1.0657) |
| -0.9346(-1.1995,-0.6697) | 0.2088(-0.2361,0.6537) | 0.1573(-0.1477,0.4623) | -0.5811(-1.0197,-0.1424) |
| -0.7237(-1.0695,-0.3779) | 0.333(0.0637,0.6023) | 0.4346(0.1861,0.6831) | -1.0057(-1.5198,-0.4916) |
| -0.763(-1.2451,-0.2809) | 0.3456(0.0633,0.6279) | 0.484(0.285,0.683) | -0.2829(-0.4797,-0.086) |
| -0.8391(-1.3026,-0.3755) | 0.3895(0.0905,0.6885) | 0.5528(0.4406,0.6651) | -0.0654(-0.1909,0.0601) |
| -0.5822(-1.1385,-0.0258) | 0.2692(-0.1049,0.6433) | 0.4355(0.2971,0.5739) | -0.2524(-0.727,0.2222) |

***(b) High-resource connected meta-ecosystems***

*High resource ecosystem*

| 0.2534(0.0825,0.4244) 0 | .1083(-0.1298,0.3464) 0 | .141(-0.0277,0.3097) - | 0.027(-0.1612,0.1071) |
| --- | --- | --- | --- |
| 0.306(0.1568,0.4551) | 0.1724(0.0257,0.319) | 0.0284(-0.1295,0.1863) | -0.4497(-0.6634,-0.2359) |
| 0.3332(0.1889,0.4775) | 0.0921(-0.0727,0.2569) | -0.214(-0.3311,-0.0968) | -0.869(-1.1622,-0.5758) |
| 0.3081(0.187,0.4293) | 0.0072(-0.1819,0.1962) | -0.234(-0.526,0.0581) | -1.0923(-1.5154,-0.6692) |
| 0.2503(0.0038,0.4969) | -0.2465(-0.5379,0.0449) | -0.3497(-0.6385,-0.0608) | -1.249(-1.4718,-1.0261) |
| 0.304(-0.0809,0.689) | -0.069(-0.348,0.21) | -0.67(-0.8962,-0.4438) | -1.062(-1.3454,-0.7785) |
| 0.1892(-0.1604,0.5387) | -0.1655(-0.5227,0.1916) | -0.7906(-1.0539,-0.5273) | -1.1545(-1.6711,-0.638) |
| 0.1553(-0.2124,0.523) | -0.4188(-0.7873,-0.0502) | -0.8638(-1.1742,-0.5535) | -0.9041(-1.2425,-0.5657) |
| 0.0576(-0.178,0.2932) | -0.6333(-1.0193,-0.2472) | -0.6578(-1.0406,-0.275) | -0.9199(-1.274,-0.5657) |
| -0.0229(-0.2264,0.1805) | -0.3231(-0.8241,0.1779) | -0.6985(-1.0461,-0.3509) | -0.9132(-1.4112,-0.4153) |

**Table S13-A**: Statistical summary table from PERMANOVA on Hellinger-transformed size class abundances at each time point. Hellinger-transformed size class abundances are used as response variable, disturbance intensity and subsidy flow are used as fixed variables and replicate as a random factor. Analysis is performed for the local low- and high-resource ecosystems separately due to non-independence of some experimental treatments. In the table we report degrees of freedom (df), sum of squares (SS), mean sum of squares (MS), F-statistic (F-value), R² and p-value. Significant effects (p < 0.005) are indicated in bold. The results using the patch 2 experimental units are reported in Supplementary Table S13-B.

***(a) Low-resource ecosystems***

|  | df | SS | F-value | R² | p-value |
| --- | --- | --- | --- | --- | --- |
| **Time point 1** |  |  |  |  |  |
| Disturbance | 3 | 0.02 | 0.57 | 0.025 | 0.905 |
| Subsidy flow | 2 | 0.05 | 1.81 | 0.052 | 0.078 |
| Disturbance x Subsidy flow | 6 | 0.07 | 0.87 | 0.075 | 0.700 |
| **Time point 2** |  |  |  |  |  |
| **Disturbance** | **3** | **0.27** | **6.94** | **0.232** | **< 0.001** |
| **Subsidy flow** | **2** | **0.06** | **2.12** | **0.047** | **0.038** |
| Disturbance x Subsidy flow | 6 | 0.10 | 1.31 | 0.088 | 0.200 |
| **Time point 3** |  |  |  |  |  |
| **Disturbance** | **3** | **0.42** | **8.67** | **0.286** | **< 0.001** |
| Subsidy flow | 2 | 0.03 | 0.95 | 0.021 | 0.446 |
| Disturbance x Subsidy flow | 6 | 0.06 | 0.67 | 0.046 | 0.860 |
| **Time point 4** |  |  |  |  |  |
| **Disturbance** | **3** | **0.24** | **7.22** | **0.228** | **< 0.001** |
| Subsidy flow | 2 | 0.02 | 1.01 | 0.021 | 0.439 |
| **Disturbance x Subsidy flow** | **6** | **0.15** | **2.21** | **0.140** | **0.002** |
| **Time point 5** |  |  |  |  |  |
| **Disturbance** | **3** | **0.31** | **7.49** | **0.228** | **< 0.001** |
| **Subsidy flow** | **2** | **0.07** | **2.58** | **0.052** | **0.009** |
| **Disturbance x Subsidy flow** | **6** | **0.16** | **1.98** | **0.121** | **0.013** |
| **Time point 6** |  |  |  |  |  |
| **Disturbance** | **3** | **0.64** | **9.13** | **0.304** | **< 0.001** |
| Subsidy flow | 2 | 0.02 | 0.52 | 0.012 | 0.828 |
| Disturbance x subsidy flow | 6 | 0.07 | 0.47 | 0.031 | 0.979 |
| **Time point 7** |  |  |  |  |  |
| **Disturbance** | **3** | **0.74** | **12.96** | **0.376** | **< 0.001** |
| Subsidy flow | 2 | 0.05 | 1.30 | 0.025 | 0.256 |
| Disturbance x subsidy flow | 6 | 0.08 | 0.67 | 0.039 | 0.890 |
| **Time point 8** |  |  |  |  |  |
| **Disturbance** | **3** | **0.63** | **10.75** | **0.313** | **< 0.001** |
| **Subsidy flow** | **2** | **0.10** | **2.48** | **0.048** | **0.019** |
| Disturbance x subsidy flow | 6 | 0.13 | 1.13 | 0.066 | 0.351 |
| **Time point 9** |  |  |  |  |  |
| **Disturbance** | **3** | **1.07** | **16.71** | **0.412** | **< 0.001** |
| Subsidy flow | 2 | 0.07 | 1.63 | 0.027 | 0.169 |
| **Disturbance x subsidy flow** | **6** | **0.24** | **1.90** | **0.094** | **0.042** |
| **Time point 10** |  |  |  |  |  |
| **Disturbance** | **3** | **1.85** | **19.86** | **0.494** | **< 0.001** |
| Subsidy flow | 2 | 0.03 | 0.45 | 0.008 | 0.777 |
| Disturbance x subsidy flow | 6 | 0.10 | 0.51 | 0.025 | 0.925 |
| **Time point 11** |  |  |  |  |  |
| **Disturbance** | **3** | **1.02** | **14.25** | **0.381** | **< 0.001** |
| Subsidy flow | 2 | 0.05 | 0.95 | 0.017 | 0.486 |
| Disturbance x subsidy flow | 6 | 0.20 | 1.41 | 0.076 | 0.154 |

***(b) High-resource ecosystems***

|  | df | SS | F-value | R² | p-value |
| --- | --- | --- | --- | --- | --- |
| **Time point 1** |  |  |  |  |  |
| Disturbance | 3 | 0.02 | 0.82 | 0.058 | 0.700 |
| Subsidy flow | 2 | 0.01 | 0.56 | 0.013 | 0.738 |
| Disturbance x subsidy flow | 6 | 0.03 | 0.93 | 0.065 | 0.590 |
| **Time point 2** |  |  |  |  |  |
| **Disturbance** | **3** | **0.13** | **4.59** | **0.249** | **< 0.001** |
| Subsidy flow | 2 | 0.01 | 1.34 | 0.024 | 0.254 |
| Disturbance x subsidy flow | 6 | 0.03 | 1.08 | 0.059 | 0.409 |
| **Time point 3** |  |  |  |  |  |
| **Disturbance** | **3** | **0.24** | **7.77** | **0.342** | **< 0.001** |
| Subsidy flow | 2 | 0.01 | 1.34 | 0.020 | 0.251 |
| Disturbance x subsidy flow | 6 | 0.05 | 1.53 | 0.067 | 0.159 |
| **Time point 4** |  |  |  |  |  |
| **Disturbance** | **3** | **0.44** | **17.90** | **0.544** | **< 0.001** |
| Subsidy flow | 2 | 0.00 | 0.36 | 0.004 | 0.795 |
| Disturbance x subsidy flow | 6 | 0.04 | 1.54 | 0.047 | 0.195 |
| **Time point 5** |  |  |  |  |  |
| **Disturbance** | **3** | **0.27** | **9.18** | **0.393** | **< 0.001** |
| Subsidy flow | 2 | 0.00 | 0.38 | 0.005 | 0.769 |
| **Disturbance x subsidy flow** | **6** | **0.06** | **2.07** | **0.089** | **0.048** |
| **Time point 6** |  |  |  |  |  |
| **Disturbance** | **3** | **0.31** | **10.36** | **0.425** | **< 0.001** |
| Subsidy flow | 2 | 0.01 | 0.80 | 0.011 | 0.490 |
| Disturbance x subsidy flow | 6 | 0.03 | 1.08 | 0.044 | 0.401 |
| **Time point 7** |  |  |  |  |  |
| **Disturbance** | **3** | **0.36** | **15.50** | **0.512** | **< 0.001** |
| Subsidy flow | 2 | 0.01 | 1.72 | 0.019 | 0.181 |
| Disturbance x subsidy flow | 6 | 0.03 | 1.18 | 0.039 | 0.368 |
| **Time point 8** |  |  |  |  |  |
| **Disturbance** | **3** | **0.30** | **11.95** | **0.447** | **< 0.001** |
| Subsidy flow | 2 | 0.01 | 1.48 | 0.018 | 0.225 |
| Disturbance x subsidy flow | 6 | 0.04 | 1.61 | 0.060 | 0.174 |
| **Time point 9** |  |  |  |  |  |
| **Disturbance** | **3** | **0.26** | **9.70** | **0.394** | **< 0.001** |
| Subsidy flow | 2 | 0.01 | 0.78 | 0.011 | 0.499 |
| Disturbance x subsidy flow | 6 | 0.04 | 1.34 | 0.054 | 0.239 |
| **Time point 10** |  |  |  |  |  |
| **Disturbance** | **3** | **0.34** | **13.01** | **0.459** | **< 0.001** |
| Subsidy flow | 2 | 0.01 | 0.59 | 0.007 | 0.595 |
| Disturbance x subsidy flow | 6 | 0.05 | 1.81 | 0.063 | 0.135 |
| **Time point 11** |  |  |  |  |  |
| **Disturbance** | **3** | **0.26** | **6.73** | **0.329** | **< 0.001** |
| Subsidy flow | 2 | 0.00 | 0.10 | 0.002 | 0.951 |
| Disturbance x subsidy flow | 6 | 0.03 | 0.72 | 0.035 | 0.675 |

**Table S13-B**: Statistical summary table from PERMANOVA on Hellinger-transformed size class abundances at each time point using the patch 2 experimental units. Hellinger-transformed size class abundances are used as response variable, disturbance intensity and subsidy flow are used as fixed variables and replicate as a random factor. Analysis is performed for the local low- and high-resource ecosystems separately due to non-independence of some experimental treatments. In the table we report degrees of freedom (df), sum of squares (SS), mean sum of squares (MS), F-statistic (F-value), R² and p-value. Significant effects (p < 0.005) are indicated in bold.

***(a) Low-resource ecosystems***

|  | df | SS | F-value | R² | p-value |
| --- | --- | --- | --- | --- | --- |
| **Time point 1** |  |  |  |  |  |
| Disturbance | 3 | 0.02 | 0.28 | 0.013 | 0.987 |
| Subsidy flow | 2 | 0.03 | 1.17 | 0.035 | 0.322 |
| Disturbance x Subsidy flow | 6 | 0.05 | 0.60 | 0.054 | 0.941 |
| **Time point 2** |  |  |  |  |  |
| **Disturbance** | **3** | **0.24** | **6.28** | **0.215** | **< 0.001** |
| **Subsidy flow** | **2** | **0.06** | **2.26** | **0.052** | **0.020** |
| Disturbance x Subsidy flow | 6 | 0.12 | 1.54 | 0.105 | 0.069 |
| **Time point 3** |  |  |  |  |  |
| **Disturbance** | **3** | **0.43** | **8.80** | **0.290** | **< 0.001** |
| Subsidy flow | 2 | 0.03 | 0.86 | 0.019 | 0.512 |
| Disturbance x Subsidy flow | 6 | 0.06 | 0.66 | 0.044 | 0.867 |
| **Time point 4** |  |  |  |  |  |
| **Disturbance** | **3** | **0.26** | **7.12** | **0.240** | **< 0.001** |
| Subsidy flow | 2 | 0.02 | 0.62 | 0.014 | 0.785 |
| Disturbance x Subsidy flow | 6 | 0.11 | 1.54 | 0.104 | 0.057 |
| **Time point 5** |  |  |  |  |  |
| **Disturbance** | **3** | **0.32** | **9.38** | **0.271** | **< 0.001** |
| **Subsidy flow** | **2** | **0.07** | **3.00** | **0.058** | **0.009** |
| **Disturbance x Subsidy flow** | **6** | **0.12** | **1.76** | **0.102** | **0.021** |
| **Time point 6** |  |  |  |  |  |
| **Disturbance** | **3** | **0.92** | **14.20** | **0.388** | **< 0.001** |
| Subsidy flow | 2 | 0.07 | 1.55 | 0.028 | 0.201 |
| Disturbance x subsidy flow | 6 | 0.11 | 0.83 | 0.046 | 0.665 |
| **Time point 7** |  |  |  |  |  |
| **Disturbance** | **3** | **0.64** | **11.83** | **0.355** | **< 0.001** |
| Subsidy flow | 2 | 0.04 | 1.13 | 0.023 | 0.317 |
| Disturbance x subsidy flow | 6 | 0.11 | 1.02 | 0.061 | 0.445 |
| **Time point 8** |  |  |  |  |  |
| **Disturbance** | **3** | **0.54** | **10.93** | **0.314** | **< 0.001** |
| **Subsidy flow** | **2** | **0.10** | **3.03** | **0.046** | **0.009** |
| Disturbance x subsidy flow | 6 | 0.08 | 0.78 | 0.046 | 0.815 |
| **Time point 9** |  |  |  |  |  |
| **Disturbance** | **3** | **0.66** | **11.15** | **0.303** | **< 0.001** |
| **Subsidy flow** | **2** | **0.10** | **2.45** | **0.044** | **0.033** |
| **Disturbance x subsidy flow** | **6** | **0.30** | **2.53** | **0.137** | **0.004** |
| **Time point 10** |  |  |  |  |  |
| **Disturbance** | **3** | **1.33** | **13.52** | **0.404** | **< 0.001** |
| Subsidy flow | 2 | 0.03 | 0.52 | 0.010 | 0.712 |
| Disturbance x subsidy flow | 6 | 0.06 | 0.32 | 0.019 | 0.995 |
| **Time point 11** |  |  |  |  |  |
| **Disturbance** | **3** | **0.84** | **11.28** | **0.322** | **< 0.001** |
| Subsidy flow | 2 | 0.05 | 1.02 | 0.019 | 0.426 |
| **Disturbance x subsidy flow** | **6** | **0.26** | **1.73** | **0.099** | **0.040** |

***(b) High-resource ecosystems***

|  | df | SS | F-value | R² | p-value |
| --- | --- | --- | --- | --- | --- |
| **Time point 1** |  |  |  |  |  |
| Disturbance | 3 | 0.03 | 1.07 | 0.049 | 0.425 |
| Subsidy flow | 2 | 0.01 | 0.63 | 0.019 | 0.783 |
| Disturbance x subsidy flow | 6 | 0.04 | 0.67 | 0.061 | 0.900 |
| **Time point 2** |  |  |  |  |  |
| **Disturbance** | **3** | **0.20** | **6.13** | **0.239** | **< 0.001** |
| Subsidy flow | 2 | 0.01 | 0.50 | 0.013 | 0.825 |
| Disturbance x subsidy flow | 6 | 0.04 | 0.60 | 0.047 | 0.905 |
| **Time point 3** |  |  |  |  |  |
| **Disturbance** | **3** | **0.41** | **14.93** | **0.401** | **< 0.001** |
| Subsidy flow | 2 | 0.04 | 2.02 | 0.036 | 0.085 |
| Disturbance x subsidy flow | 6 | 0.06 | 1.14 | 0.061 | 0.354 |
| **Time point 4** |  |  |  |  |  |
| **Disturbance** | **3** | **0.59** | **22.62** | **0.497** | **< 0.001** |
| Subsidy flow | 2 | 0.03 | 1.61 | 0.024 | 0.189 |
| Disturbance x subsidy flow | 6 | 0.06 | 1.06 | 0.047 | 0.427 |
| **Time point 5** |  |  |  |  |  |
| **Disturbance** | **3** | **0.50** | **18.95** | **0.447** | **< 0.001** |
| **Subsidy flow** | **2** | **0.05** | **2.56** | **0.040** | **0.047** |
| Disturbance x subsidy flow | 6 | 0.08 | 1.52 | 0.072 | 0.152 |
| **Time point 6** |  |  |  |  |  |
| **Disturbance** | **3** | **0.47** | **15.85** | **0.422** | **< 0.001** |
| Subsidy flow | 2 | 0.04 | 1.88 | 0.033 | 0.113 |
| Disturbance x subsidy flow | 6 | 0.06 | 1.04 | 0.055 | 0.449 |
| **Time point 7** |  |  |  |  |  |
| **Disturbance** | **3** | **0.43** | **16.33** | **0.429** | **< 0.001** |
| Subsidy flow | 2 | 0.02 | 1.36 | 0.024 | 0.229 |
| Disturbance x subsidy flow | 6 | 0.07 | 1.39 | 0.073 | 0.183 |
| **Time point 8** |  |  |  |  |  |
| **Disturbance** | **3** | **0.33** | **11.98** | **0.363** | **< 0.001** |
| Subsidy flow | 2 | 0.01 | 0.76 | 0.015 | 0.615 |
| Disturbance x subsidy flow | 6 | 0.07 | 1.24 | 0.075 | 0.278 |
| **Time point 9** |  |  |  |  |  |
| **Disturbance** | **3** | **0.40** | **14.22** | **0.377** | **< 0.001** |
| Subsidy flow | 2 | 0.02 | 0.83 | 0.015 | 0.523 |
| Disturbance x subsidy flow | 6 | 0.09 | 1.65 | 0.087 | 0.090 |
| **Time point 10** |  |  |  |  |  |
| **Disturbance** | **3** | **0.39** | **14.30** | **0.389** | **< 0.001** |
| Subsidy flow | 2 | 0.02 | 1.06 | 0.019 | 0.416 |
| Disturbance x subsidy flow | 6 | 0.07 | 1.20 | 0.066 | 0.321 |
| **Time point 11** |  |  |  |  |  |
| **Disturbance** | **3** | **0.42** | **12.24** | **0.355** | **< 0.001** |
| Subsidy flow | 2 | 0.01 | 0.44 | 0.009 | 0.846 |
| Disturbance x subsidy flow | 6 | 0.07 | 0.96 | 0.056 | 0.532 |

**Table S14-A**: Log-response ratio of size class diversity at each time point comparing each experimental treatment combination to the control, undisturbed isolated local low-resource ecosystem for (a) isolated ecosystems, (b) ecosystems connected to a low-resource ecosystem and (c) ecosystems connected to a high-resource ecosystem. The first value gives the log-response ratio estimate and values between brackets indicate the 95% confidence interval. Values in bold reflect where the 95% confidence interval does not overlap with zero. The results using the patch 2 experimental units are reported in Supplementary Table S14-B.

***(a) Unconnected meta-ecosystems***

*Low resource ecosystem*

|  | 0.0547(-0.4645,0.5739) | 0.205(-0.2205,0.6305) | 0.0325(-0.4392,0.5042) |
| --- | --- | --- | --- |
|  | 0.0642(-0.5074,0.6358) | 0.1447(-0.3768,0.6662) | -0.3935(-0.9241,0.137) |
|  | 0.1711(-0.4149,0.7571) | 0.1153(-0.5501,0.7806) | 0.0148(-0.5862,0.6158) |
|  | -0.0862(-0.5612,0.3887) | 0.4769(0.1629,0.7909) | 0.2108(-0.0894,0.511) |
|  | 0.0545(-0.4143,0.5233) | 0.508(0.1782,0.8377) | -0.774(-1.32,-0.2281) |
|  | 0.2865(-0.1824,0.7554) | 0.6383(0.3562,0.9205) | -0.4659(-0.9162,-0.0156) |
|  | 0.1615(-0.3435,0.6665) | 0.4589(0.0097,0.9082) | -0.813(-1.3578,-0.2682) |
|  | 0.5156(0.1006,0.9307) | 0.7709(0.3367,1.2051) | -0.6791(-2.52,1.1618) |
|  | 0.5156(-0.04,1.0711) | 0.8877(0.3584,1.4169) | -0.3633(-1.645,0.9184) |
|  | 0.7453(0.1401,1.3505) | 1.1198(0.5563,1.6832) | -0.418(-1.4299,0.5938) |

*High resource ecosystem*

| 0.6691(0.2607,1.0775) | 0.5841(0.1362,1.0319) | 0.5032(0.038,0.9684) | 0.327(-0.1429,0.797) |
| --- | --- | --- | --- |
| 1.0474(0.549,1.5459) | 0.8046(0.2919,1.3173) | 0.5678(0.0311,1.1045) | 0.2148(-0.3068,0.7364) |
| 1.1519(0.6291,1.6748) | 0.4935(-0.1302,1.1173) | 0.2172(-0.3382,0.7726) | 0.0019(-0.5685,0.5723) |
| 1.3267(1.1386,1.5148) | 0.5563(0.125,0.9877) | 0.2241(-0.2318,0.6801) | 0.1104(-0.1443,0.365) |
| 1.1349(0.854,1.4159) | 0.319(-0.1347,0.7726) | 0.0147(-0.3359,0.3653) | -0.2782(-0.7081,0.1517) |
| 1.1662(0.9173,1.4151) | 0.3332(-0.0229,0.6893) | 0.0024(-0.2891,0.2939) | -0.3652(-0.7934,0.0629) |
| 0.9734(0.5488,1.398) | 0.1409(-0.3271,0.609) | -0.0674(-0.5779,0.4431) | -0.4188(-1.0182,0.1806) |
| 1.1192(0.6788,1.5595) | 0.1598(-0.4095,0.7291) | -0.0069(-0.515,0.5012) | -0.4742(-0.9093,-0.0391) |
| 1.2268(0.6878,1.7658) | 0.5006(-0.1179,1.1191) | 0.1278(-0.5846,0.8402) | -0.69(-1.3295,-0.0505) |
| 1.2316(0.6414,1.8217) | 0.4466(-0.1783,1.0716) | 0.2933(-0.3198,0.9065) | -0.5082(-1.1594,0.1429) |

***(b) Low-resource connected meta-ecosystems***

*Low resource ecosystem*

| 0.1635(-0.3147,0.6417) | 0.2094(-0.2702,0.689) | 0.362(-0.1121,0.8361) | 0.1223(-0.3883,0.633) |
| --- | --- | --- | --- |
| -0.0866(-0.7492,0.576) | -0.022(-0.6362,0.5923) | 0.2456(-0.3094,0.8006) | -0.1649(-0.7254,0.3957) |
| -0.3133(-1.001,0.3745) | -0.1927(-1.0272,0.6418) | 0.2253(-0.3503,0.801) | 0.0128(-0.5665,0.5921) |
| -0.4436(-0.8967,0.0096) | 0.0156(-0.5042,0.5353) | 0.5094(0.2063,0.8124) | 0.0642(-0.3232,0.4516) |
| **-0.6019(-0.9451,-0.2586)** | 0.0589(-0.5339,0.6517) | 0.4631(0.0734,0.8528) | -0.8597(-1.2192,-0.5001) |
| **-0.358(-0.6064,-0.1096)** | 0.3764(-0.1248,0.8775) | 0.5264(0.1933,0.8595) | -0.8197(-1.4458,-0.1936) |
| -0.4324(-0.8762,0.0114) | 0.3015(-0.2803,0.8834) | 0.7879(0.3596,1.2163) | -1.1079(-1.6729,-0.5429) |
| **-0.4785(-0.9274,-0.0296)** | 0.5409(-0.0578,1.1395) | 0.8938(0.4531,1.3346) | -0.5455(-1.1057,0.0146) |
| **-0.6389(-1.2144,-0.0634)** | 0.6882(0.0682,1.3082) | 0.8863(0.3639,1.4087) | -1.0122(-2.0709,0.0465) |
| **-0.724(-1.2875,-0.1605)** | 0.7905(0.1997,1.3813) | 0.9369(0.3797,1.4941) | 0.135(-0.6329,0.9029) |

*High resource ecosystem*

| 0.6823(0.257,1.1076) | 0.4641(-0.0354,0.9635) | 0.5348(0.1053,0.9642) | 0.2615(-0.2194,0.7425) |
| --- | --- | --- | --- |
| 1.0016(0.4627,1.5405) | 0.5869(0.0095,1.1643) | 0.652(0.1228,1.1811) | 0.213(-0.3161,0.7421) |
| 0.9233(0.2968,1.5499) | 0.3571(-0.2369,0.9512) | 0.5471(-0.0119,1.1061) | -0.0268(-0.638,0.5845) |
| 1.0385(0.6926,1.3844) | 0.2876(-0.0058,0.581) | 0.4784(0.1676,0.7893) | -0.0283(-0.2679,0.2112) |
| 0.9243(0.5722,1.2763) | 0.0588(-0.4072,0.5248) | 0.1063(-0.3118,0.5245) | -0.4787(-1.0326,0.0752) |
| 0.9324(0.5953,1.2694) | -0.0622(-0.3922,0.2678) | -0.0847(-0.3748,0.2053) | -0.6241(-1.238,-0.0102) |
| 0.8381(0.3888,1.2873) | -0.2347(-0.7473,0.2779) | -0.2425(-0.8129,0.3279) | -0.7456(-1.3232,-0.168) |
| 1.0322(0.5814,1.483) | -0.2993(-0.9401,0.3416) | -0.1548(-0.908,0.5984) | -0.7089(-1.1389,-0.2789) |
| 1.2078(0.6774,1.7382) | 0.098(-0.5548,0.7509) | 0.1198(-0.5385,0.7781) | -0.482(-1.0891,0.1252) |
| 1.1557(0.5634,1.748) | 0.2328(-0.3403,0.8058) | 0.2622(-0.3298,0.8541) | -0.4917(-1.1242,0.1408) |

***(c) High-resource connected meta-ecosystems***

*Low resource ecosystem*

| 0.1929(-0.3032,0.689) | 0.3274(-0.128,0.7828) | 0.2112(-0.1908,0.6133) | -0.0273(-0.4963,0.4418) |
| --- | --- | --- | --- |
| 0.2105(-0.4552,0.8761) | 0.3458(-0.2588,0.9504) | -0.1039(-0.5966,0.3888) | -0.523(-1.2287,0.1827) |
| 0.0704(-0.6356,0.7764) | 0.3132(-0.3048,0.9313) | 0.205(-0.3669,0.777) | -0.3635(-1.0881,0.361) |
| -0.1353(-0.5453,0.2748) | 0.3322(-0.1443,0.8086) | 0.3322(0.083,0.5813) | 0.0912(-0.2345,0.4169) |
| -0.2273(-0.8591,0.4044) | 0.2179(-0.298,0.7338) | 0.0694(-0.3289,0.4677) | -0.3196(-0.8193,0.18) |
| -0.0747(-0.6875,0.5381) | 0.3577(-0.0644,0.7798) | 0.256(0.0251,0.4869) | -0.5939(-1.0466,-0.1411) |
| -0.2455(-1.0637,0.5726) | 0.3595(-0.1561,0.8751) | 0.4851(0.0641,0.9061) | -0.2571(-0.7532,0.239) |
| -0.3302(-1.039,0.3787) | 0.4172(-0.2301,1.0645) | 0.5775(0.1595,0.9956) | 0.0729(-0.4039,0.5497) |
| -0.6405(-1.3685,0.0876) | 0.5398(-0.0803,1.1599) | 0.3443(-0.2313,0.92) | -0.2772(-1.6364,1.082) |
| **-0.8112(-1.4242,-0.1981)** | 0.1815(-0.4846,0.8475) | 0.629(-0.0172,1.2751) | 0.4038(-0.4343,1.2419) |

*High resource ecosystem*

| 0.6546(0.2187,1.0905) | 0.5393(0.128,0.9506) | 0.382(-0.0858,0.8499) | 0.215(-0.1884,0.6185) |
| --- | --- | --- | --- |
| 0.9673(0.4555,1.4791) | 0.6739(0.1362,1.2116) | 0.4233(-0.1317,0.9783) | 0.2428(-0.3181,0.8037) |
| 0.9349(0.382,1.4879) | 0.6548(0.0686,1.241) | 0.2791(-0.3011,0.8593) | 0.0224(-0.5536,0.5984) |
| 0.9177(0.6473,1.188) | 0.7892(0.4841,1.0944) | 0.2196(-0.3382,0.7774) | -0.3889(-0.643,-0.1348) |
| 0.8025(0.3868,1.2182) | 0.4111(0.0547,0.7675) | 0.0051(-0.5201,0.5302) | -0.559(-1.1616,0.0435) |
| 0.8196(0.4883,1.1509) | 0.1988(-0.0589,0.4565) | -0.1247(-0.6303,0.3808) | -0.8307(-1.2646,-0.3968) |
| 0.6232(0.1727,1.0737) | -0.1177(-0.5741,0.3387) | -0.3915(-0.8206,0.0376) | -0.8358(-1.3779,-0.2937) |
| 0.7666(0.3051,1.2282) | -0.0032(-0.5576,0.5511) | -0.2048(-0.8706,0.461) | -0.6238(-1.1424,-0.1052) |
| 0.8638(0.3235,1.4041) | 0.1465(-0.4995,0.7926) | -0.0146(-0.6868,0.6575) | -0.552(-1.272,0.168) |
| 0.9421(0.3683,1.5158) | 0.2803(-0.4019,0.9625) | 0.0959(-0.5319,0.7238) | -0.6414(-1.2751,-0.0076) |

**Table S14-B**: Log-response ratio of size class diversity at each time point comparing each experimental treatment combination to the control, undisturbed isolated local low-resource ecosystem for (a) isolated ecosystems, (b) ecosystems connected to a low-resource ecosystem and (c) ecosystems connected to a high-resource ecosystem using the patch 2 experimental units. The first value gives the log-response ratio estimate and values between brackets indicate the 95% confidence interval. Values in bold reflect where the 95% confidence interval does not overlap with zero.

***(a) Low-resource connected meta-ecosystems***

*Low resource ecosystem*

| 0.1439(-0.3176,0.6054) | 0.4137(-0.0779,0.9053) | 0.0831(-0.3865,0.5528) | 0.3343(-0.2114,0.88) |
| --- | --- | --- | --- |
| 0.1061(-0.4992,0.7115) | 0.2497(-0.341,0.8404) | 0.2451(-0.2538,0.744) | -0.1543(-0.8957,0.5871) |
| -0.2121(-0.7882,0.3641) | 0.0975(-0.5772,0.7722) | 0.3007(-0.3641,0.9655) | 0.0036(-0.5853,0.5924) |
| -0.1426(-0.3713,0.0861) | 0.1811(-0.2812,0.6434) | 0.3739(0.0771,0.6706) | -0.1982(-0.4604,0.0641) |
| -0.4308(-0.7076,-0.154) | 0.4003(-0.1462,0.9467) | 0.362(-0.0478,0.7718) | -1.4906(-1.8304,-1.1508) |
| -0.4724(-0.7937,-0.1512) | 0.584(0.1227,1.0453) | 0.5976(0.2422,0.9531) | -0.4039(-1.0139,0.2062) |
| -0.4221(-0.8682,0.0241) | 0.5496(0.1347,0.9645) | 0.6754(0.2337,1.1171) | -0.9162(-1.662,-0.1705) |
| -0.36(-0.9457,0.2258) | 0.7404(0.2847,1.196) | 0.8184(0.3773,1.2595) | 0.0414(-0.399,0.4818) |
| -0.2636(-0.9857,0.4585) | 0.8921(0.3309,1.4534) | 1.0623(0.5519,1.5727) | -0.0411(-0.8574,0.7752) |
| 0.0101(-0.794,0.8143) | 0.8544(0.2056,1.5031) | 0.9507(0.3839,1.5175) | 0.1774(-0.5029,0.8577) |

***(b) High-resource connected meta-ecosystems***

*High resource ecosystem*

| 0.7422(0.3105,1.1738) | 0.564(0.1029,1.0252) | 0.5285(0.1104,0.9465) | 0.305(-0.1015,0.7116) |
| --- | --- | --- | --- |
| 0.9703(0.4711,1.4695) | 0.7979(0.2896,1.3063) | 0.5739(0.0607,1.0871) | 0.0175(-0.4872,0.5222) |
| 0.9881(0.4599,1.5163) | 0.6626(0.1271,1.1981) | 0.2851(-0.2455,0.8156) | -0.4502(-1.0258,0.1254) |
| 1.0465(0.8352,1.2579) | 0.7379(0.4874,0.9884) | 0.4222(0.1044,0.7399) | -0.4964(-0.9524,-0.0403) |
| 0.8328(0.4852,1.1805) | 0.2785(-0.0506,0.6077) | 0.2208(-0.1864,0.628) | -0.7538(-1.1078,-0.3998) |
| 0.8594(0.5375,1.1813) | 0.5631(0.2223,0.9038) | -0.0477(-0.3194,0.224) | -0.5641(-0.8895,-0.2388) |
| 0.6244(0.1621,1.0868) | 0.2631(-0.2311,0.7572) | -0.3102(-0.7514,0.1309) | -0.7685(-1.313,-0.224) |
| 0.6756(0.1956,1.1556) | 0.1119(-0.3623,0.5861) | -0.2665(-0.7715,0.2385) | -0.4634(-0.9822,0.0555) |
| 0.7866(0.2657,1.3075) | 0.0732(-0.5663,0.7126) | 0.0077(-0.5904,0.6058) | -0.2989(-0.8858,0.2879) |
| 0.7695(0.2076,1.3313) | 0.452(-0.3085,1.2125) | 0.106(-0.5103,0.7223) | -0.2748(-0.9515,0.4019) |

**Table S15-A**: Log-response ratio of size class evenness at each time point comparing each experimental treatment combination to the control, undisturbed isolated local low-resource ecosystem for (a) isolated ecosystems, (b) ecosystems connected to a low-resource ecosystem and (c) ecosystems connected to a high-resource ecosystem. The first value gives the log-response ratio estimate and values between brackets indicate the 95% confidence interval. Values in bold reflect where the 95% confidence interval does not overlap with zero. The results using the patch 2 experimental units are reported in Supplementary Table S14-B.

***(a) Unconnected meta-ecosystems***

*Low resource ecosystem*

|  | 0.1774(-0.2706,0.6253) 0 | .3512(-0.0217,0.724) 0 | .2116(-0.2059,0.6291) |
| --- | --- | --- | --- |
|  | 0.2095(-0.3728,0.7917) | 0.3285(-0.1569,0.8139) | -0.141(-0.6788,0.3969) |
|  | 0.2518(-0.3731,0.8767) | 0.2097(-0.4775,0.8968) | 0.1373(-0.4926,0.7672) |
|  | -0.0413(-0.5199,0.4373) | 0.5887(0.2175,0.9599) | 0.3934(0.071,0.7157) |
|  | 0.0805(-0.4076,0.5687) | 0.4824(0.12,0.8447) | -0.4806(-0.9794,0.0183) |
|  | 0.3943(-0.0833,0.872) | 0.6876(0.3485,1.0267) | -0.1946(-0.5937,0.2046) |
|  | 0.2414(-0.2415,0.7242) | 0.5794(0.1442,1.0146) | -0.5232(-1.0571,0.0107) |
|  | 0.5744(0.1832,0.9657) | 0.9131(0.5123,1.3138) | 0.2701(-0.971,1.5113) |
|  | 0.638(0.1315,1.1444) | 1.0619(0.5946,1.5291) | 0.036(-0.8858,0.9579) |
|  | 0.7433(0.124,1.3626) | 1.1948(0.6223,1.7674) | -0.0069(-0.9751,0.9614) |

*High resource ecosystem*

| 0.6312(0.2727,0.9897) | 0.5737(0.1774,0.9701) 0 | .5492(0.1456,0.9528) 0 | .4259(0.0159,0.8358) |
| --- | --- | --- | --- |
| 0.9908(0.5153,1.4663) | 0.852(0.3642,1.3399) | 0.5237(0.0105,1.037) | 0.3496(-0.1515,0.8506) |
| 0.9651(0.3916,1.5387) | 0.4183(-0.2395,1.0761) | 0.1779(-0.4295,0.7853) | 0.0594(-0.5634,0.6822) |
| 1.1585(0.9171,1.3999) | 0.5299(0.0653,0.9946) | 0.1964(-0.2308,0.6235) | 0.1247(-0.2679,0.5174) |
| 0.9447(0.5993,1.2901) | 0.2272(-0.242,0.6964) | -0.0745(-0.466,0.317) | -0.2995(-0.755,0.156) |
| 0.9997(0.7346,1.2648) | 0.3169(-0.0414,0.6752) | 0.0036(-0.3368,0.344) | -0.4018(-0.7974,-0.0061) |
| 0.8595(0.4414,1.2776) | 0.1784(-0.3347,0.6915) | -0.1253(-0.6597,0.4091) | -0.4266(-0.977,0.1238) |
| 1.0516(0.6184,1.4849) | 0.2097(-0.3599,0.7794) | -0.0228(-0.587,0.5413) | -0.4293(-0.8292,-0.0294) |
| 1.1288(0.6282,1.6293) | 0.5383(-0.0506,1.1271) | 0.1062(-0.6017,0.8142) | -0.6542(-1.2351,-0.0732) |
| 1.1032(0.5221,1.6843) | 0.4763(-0.185,1.1377) | 0.2336(-0.446,0.9133) | -0.4445(-1.1103,0.2212) |

***(b) Low-resource connected meta-ecosystems***

*Low resource ecosystem*

| 0.1885(-0.2815,0.6585) | 0.2097(-0.1982,0.6176) | 0.4605(0.0766,0.8443) | 0.2771(-0.1738,0.728) |
| --- | --- | --- | --- |
| -0.0238(-0.6557,0.6082) | 0.0588(-0.537,0.6546) | 0.4171(-0.1204,0.9545) | 0.0512(-0.5009,0.6032) |
| -0.2857(-1.0256,0.4543) | -0.0585(-0.9257,0.8086) | 0.2506(-0.3894,0.8906) | 0.1928(-0.4319,0.8175) |
| -0.384(-0.8536,0.0857) | 0.1183(-0.4444,0.6811) | 0.5139(0.1524,0.8755) | 0.28(-0.0972,0.6572) |
| **-0.5814(-0.9907,-0.1721)** | 0.1018(-0.5367,0.7404) | 0.4761(0.0522,0.8999) | -0.6126(-1.0145,-0.2107) |
| **-0.2823(-0.5544,-0.0102)** | 0.4486(-0.1119,1.009) | 0.5869(0.2926,0.8813) | -0.4084(-1.1817,0.365) |
| -0.3599(-0.7843,0.0644) | 0.3578(-0.2009,0.9165) | 0.8052(0.4109,1.1995) | -0.8898(-1.3386,-0.4409) |
| **-0.5119(-0.9574,-0.0664)** | 0.6769(0.0919,1.2619) | 0.9414(0.5397,1.3431) | -0.1184(-0.5737,0.3368) |
| **-0.6076(-1.1378,-0.0775)** | 0.8021(0.1982,1.406) | 1.0117(0.5435,1.4799) | -0.1619(-0.8282,0.5043) |
| **-0.6799(-1.2469,-0.1129)** | 0.8742(0.261,1.4874) | 1.1106(0.5364,1.6848) | 0.3683(-0.3652,1.1017) |

*High resource ecosystem*

| 0.6647(0.2801,1.0493) | 0.4611(0.0044,0.9179) | 0.5478(0.176,0.9196) | 0.3775(-0.0227,0.7776) |
| --- | --- | --- | --- |
| 0.9405(0.4133,1.4677) | 0.5449(-0.0273,1.117) | 0.7075(0.2095,1.2055) | 0.3951(-0.1604,0.9506) |
| 0.7805(0.1109,1.45) | 0.2942(-0.3599,0.9483) | 0.4671(-0.1324,1.0666) | 0.063(-0.5843,0.7103) |
| 0.8549(0.4792,1.2306) | 0.2005(-0.1498,0.5509) | 0.4788(0.126,0.8315) | 0.0994(-0.1915,0.3902) |
| 0.7644(0.3518,1.177) | -0.0677(-0.5644,0.4289) | 0.0385(-0.3864,0.4634) | -0.425(-1.0405,0.1905) |
| 0.7996(0.4457,1.1535) | -0.0921(-0.4542,0.27) | -0.0912(-0.3764,0.194) | -0.6206(-1.2239,-0.0173) |
| 0.779(0.3113,1.2466) | -0.2302(-0.7418,0.2814) | -0.2143(-0.7737,0.3451) | -0.6746(-1.3062,-0.043) |
| 0.9425(0.5023,1.3827) | -0.3394(-1.025,0.3463) | -0.1581(-0.895,0.5788) | -0.6078(-1.0273,-0.1883) |
| 1.0773(0.5783,1.5763) | 0.0612(-0.5587,0.6811) | 0.0754(-0.5538,0.7046) | -0.3816(-0.9273,0.1641) |
| 1.0498(0.4173,1.6823) | 0.1601(-0.46,0.7801) | 0.242(-0.3804,0.8643) | -0.42(-1.0314,0.1913) |

***(c) High-resource connected meta-ecosystems***

*Low resource ecosystem*

| 0.1929(-0.2374,0.6232) | 0.3326(-0.0625,0.7278) | 0.2664(-0.0815,0.6143) | 0.121(-0.2756,0.5176) |
| --- | --- | --- | --- |
| 0.2564(-0.3666,0.8795) | 0.3898(-0.1724,0.9519) | 0.0617(-0.4199,0.5434) | -0.3471(-1.0413,0.3472) |
| 0.004(-0.7093,0.7174) | 0.3877(-0.2684,1.0437) | 0.2665(-0.3564,0.8894) | -0.1998(-0.9331,0.5334) |
| -0.1865(-0.6038,0.2307) | 0.2485(-0.2108,0.7079) | 0.3292(0.0435,0.6149) | 0.1964(-0.1357,0.5285) |
| -0.3192(-0.907,0.2687) | 0.132(-0.3844,0.6484) | 0.0965(-0.3482,0.5411) | -0.005(-0.338,0.328) |
| -0.1769(-0.7987,0.445) | 0.31(-0.1093,0.7294) | 0.2896(0.0454,0.5339) | -0.2993(-0.6621,0.0636) |
| -0.2789(-1.0654,0.5075) | 0.3927(-0.1105,0.8959) | 0.5187(0.1228,0.9146) | -0.0974(-0.574,0.3792) |
| -0.3286(-1.0271,0.37) | 0.4484(-0.1088,1.0057) | 0.6701(0.2597,1.0806) | 0.3255(-0.1242,0.7752) |
| -0.6207(-1.3118,0.0705) | 0.623(0.0233,1.2226) | 0.4745(-0.0383,0.9874) | 0.5669(-0.0453,1.1791) |
| **-0.8533(-1.4938,-0.2127)** | 0.2285(-0.4505,0.9074) | 0.7507(0.1016,1.3997) | 0.7475(0.0569,1.4382) |

*High resource ecosystem*

| 0.6284(0.2344,1.0223) | 0.4949(0.1356,0.8542) | 0.3655(-0.0493,0.7803) | 0.3487(-0.0132,0.7106) |
| --- | --- | --- | --- |
| 0.9207(0.4263,1.4151) | 0.6338(0.1233,1.1444) | 0.5102(-0.0024,1.0228) | 0.3227(-0.2061,0.8514) |
| 0.783(0.1794,1.3866) | 0.5465(-0.0929,1.1859) | 0.2558(-0.3708,0.8823) | 0.07(-0.5259,0.666) |
| 0.801(0.4651,1.1368) | 0.6734(0.3332,1.0137) | 0.1246(-0.4216,0.6709) | -0.3832(-0.6834,-0.0829) |
| 0.6691(0.1935,1.1446) | 0.2612(-0.1656,0.6881) | -0.0205(-0.5813,0.5402) | -0.5988(-1.237,0.0394) |
| 0.7297(0.3694,1.09) | 0.1986(-0.0925,0.4897) | -0.1392(-0.6432,0.3648) | -0.8137(-1.2267,-0.4007) |
| 0.5522(0.1163,0.9882) | -0.2558(-0.6779,0.1664) | -0.3466(-0.7827,0.0896) | -0.8186(-1.2763,-0.361) |
| 0.7168(0.271,1.1626) | -0.0773(-0.59,0.4354) | -0.1794(-0.9241,0.5653) | -0.5819(-1.1175,-0.0463) |
| 0.781(0.2883,1.2738) | 0.0818(-0.5291,0.6928) | -0.0437(-0.7212,0.6338) | -0.443(-1.128,0.2421) |
| 0.8673(0.2653,1.4692) | 0.2788(-0.4835,1.0411) | 0.0177(-0.6504,0.6858) | -0.5946(-1.2636,0.0745) |

**Table S15-B**: Log-response ratio of size class evenness at each time point comparing each experimental treatment combination to the control, undisturbed isolated local low-resource ecosystem for (a) isolated ecosystems, (b) ecosystems connected to a low-resource ecosystem and (c) ecosystems connected to a high-resource ecosystem using the patch 2 experimental units. The first value gives the log-response ratio estimate and values between brackets indicate the 95% confidence interval. Values in bold reflect where the 95% confidence interval does not overlap with zero.

***(a) Low-resource connected meta-ecosystems***

*Low resource ecosystem*

| 0.1341(-0.2665,0.5348) | 0.4152(-0.0283,0.8587) | 0.2357(-0.2028,0.6741) | 0.4944(-0.019,1.0078) |
| --- | --- | --- | --- |
| 0.1454(-0.4337,0.7244) | 0.4099(-0.1699,0.9897) | 0.3771(-0.0944,0.8485) | 0.0741(-0.6134,0.7616) |
| -0.1702(-0.7886,0.4482) | 0.1572(-0.5284,0.8427) | 0.4006(-0.2575,1.0586) | 0.1011(-0.4891,0.6912) |
| -0.1451(-0.4333,0.1431) | 0.2322(-0.2467,0.7111) | 0.4869(0.1749,0.7989) | -0.0022(-0.2964,0.2919) |
| -0.3974(-0.7349,-0.0599) | 0.3687(-0.1788,0.9163) | 0.5127(0.064,0.9614) | -0.9861(-1.4384,-0.5338) |
| -0.4656(-0.7985,-0.1327) | 0.6779(0.1894,1.1663) | 0.6264(0.2607,0.992) | -0.112(-0.5948,0.3708) |
| -0.3615(-0.8447,0.1216) | 0.6952(0.2635,1.1269) | 0.7968(0.3777,1.2158) | -0.6435(-1.2584,-0.0286) |
| -0.2729(-0.8748,0.329) | 0.8357(0.3779,1.2934) | 0.9741(0.5624,1.3857) | 0.2072(-0.2034,0.6178) |
| -0.2474(-0.8871,0.3924) | 0.9812(0.4485,1.514) | 1.1445(0.6895,1.5995) | 0.5263(0.0679,0.9848) |
| 0.0835(-0.7005,0.8675) | 0.9349(0.2677,1.6021) | 1.1012(0.5317,1.6707) | 0.4133(-0.315,1.1415) |

***(b) High-resource connected meta-ecosystems***

*High resource ecosystem*

| 0.7091(0.3344,1.0839) | 0.564(0.1543,0.9737) | 0.5967(0.223,0.9704) | 0.4287(0.0692,0.7881) |
| --- | --- | --- | --- |
| 0.9182(0.4351,1.4014) | 0.7847(0.3023,1.267) | 0.6407(0.1548,1.1266) | 0.1626(-0.3442,0.6694) |
| 0.844(0.2645,1.4236) | 0.6029(0.0179,1.1879) | 0.2968(-0.2766,0.8702) | -0.3582(-0.9914,0.2751) |
| 0.9696(0.7287,1.2105) | 0.6686(0.3874,0.9499) | 0.4275(0.0688,0.7862) | -0.4308(-0.9024,0.0407) |
| 0.7428(0.3471,1.1385) | 0.246(-0.1791,0.6711) | 0.1428(-0.2806,0.5662) | -0.7565(-1.1379,-0.3751) |
| 0.8557(0.4209,1.2906) | 0.4827(0.1381,0.8273) | -0.1183(-0.4218,0.1852) | -0.5103(-0.8585,-0.162) |
| 0.634(0.1479,1.1201) | 0.2793(-0.2123,0.7709) | -0.3458(-0.7741,0.0825) | -0.7097(-1.3269,-0.0925) |
| 0.728(0.213,1.2431) | 0.154(-0.3617,0.6697) | -0.2911(-0.767,0.1847) | -0.3314(-0.826,0.1632) |
| 0.732(0.2318,1.2322) | 0.0411(-0.5452,0.6274) | 0.0166(-0.5676,0.6007) | -0.2455(-0.8113,0.3203) |
| 0.7254(0.1365,1.3143) | 0.4252(-0.3207,1.1712) | 0.0498(-0.603,0.7027) | -0.1649(-0.9088,0.579) |
