# Supplementary material for: Local Resource Availability and Subsidy Flow Mediate the Effects of Disturbances in Meta‐Ecosystems
Source: Ecol Evol. 2026 Apr 27;16(4):e73516. doi: 10.1002/ece3.73516 (PMC13112083; doi:10.1002/ece3.73516)
Supplement: Supplementary file 2 — Figure S1: Log responses ratios of time series of log‐transformed densities comparing each time point to T0 for (a) the isolated meta‐ecosystems, (b) the low‐resource connected ecosystems and (c) the high‐resource connected ecosystems. Log response ratios of density comparing each treatment against the control (here: low resource isolated unconnected ecosystem) at each time point for (d) the isolated meta‐ecosystems, (e) the low‐resource connected ecosystems and (f) the high‐resource connected ecosystems. Blue symbols are low‐resource focal systems, while red symbols indicate high resource focal systems, both with varying levels of total perturbations (20%, 50%, 70%, 90%), respectively. Figure S2: Log response ratios of time series of evenness comparing each time point to T0 for (a) the isolated ecosystems, (b) the low‐resource connected ecosystems and (c) the high‐resource connected ecosystems. Log response ratios of evenness comparing each treatment against the control (here taken as the low resource isolated ecosystem) at each time point for (d) the isolated ecosystems, (e) the low‐resource connected ecosystems and (f) the high‐resource connected ecosystems. Blue symbols are low resource focal systems, while red symbols indicate high resource focal systems, both with varying levels of total perturbations (20%, 50%, 70%, 90%), respectively. Figure S3: Visualization of community size class compositional trajectories from the start (unfilled symbols) to the end (filled symbols) of the experiment. We used Non‐Metric Dimensional Scaling analysis on Hellinger‐transformed size class abundances. Blue (resp. red) colors represent local low‐resource (resp. high‐resource) ecosystems with circles representing isolated ecosystems, squares representing ecosystems connected to a low‐resource ecosystem and triangles representing ecosystems connected to a high‐resource ecosystem. Color intensity reflects different disturbance intensity levels. [file ECE3-16-e73516-s001.docx]

**Supplementary Information B: Supplementary Results**

***Log-response ratios for density***

For local low-resource ecosystems, disturbance had a negative impact on density, resulting in a significant decrease in the density toward the end of the experiment (Supplementary Fig. S1a-c, blue lines; Supplementary Information Table S6). Being connected to a high-resource ecosystem reduced these negative effects of disturbance, but only so for the lowest two disturbance levels (Supplementary Fig. S1c). Even more so, for the undisturbed low-resource ecosystem receiving a high-resource meta-ecosystem flow, we observed increasingly higher densities toward the end of the experiment, even exceeding densities found in the undisturbed high-resource ecosystems (Supplementary Fig. S1c). Contrary to biomass, we did not observe a decrease in densities in the disturbed local high-resource ecosystems during the first part of the experiment. Instead, we observed significantly higher densities toward the end of the experiment, but this was only for disturbed ecosystems (Supplementary Fig. S1a-c; Supplementary Information Table S6). Subsidy flow did not positively or negatively affect densities in the local high-resource ecosystem (Supplementary Fig. S1b-c).

The log response ratio analyses for densities at each time point showed again significant negative impacts of disturbance on densities for low-resource ecosystems (Supplementary Fig. S1d-f, blue colors; Supplementary Table S7). For 50 and 70% disturbance intensity, the negative effects became stronger toward the end of the experiment, whereas for the highest disturbance intensity, effects remained relatively constant throughout the experiment (Supplementary Fig. S1d-f). Undisturbed low-resource ecosystems connected to a low-resource ecosystem did not show a significant change in density compared to unconnected undisturbed low-resource ecosystems (Supplementary Fig. S1e; Supplementary Table S7-Ab). In contrast, undisturbed low-resource ecosystems connected to a high-resource ecosystem showed a steep significant increase in densities (Supplementary Fig. S1f; Supplementary Table S7A-c). High-resource ecosystems did not show a change in densities in the first half of the experiment, whereas in the second half of the experiment, significantly higher densities were found with increasing disturbance intensity, and this was found consistently across the three meta-ecosystem flow treatments (Supplementary Fig. S1d-f, Supplementary Table S7).

**Supplementary Information C: Supplementary Figures**


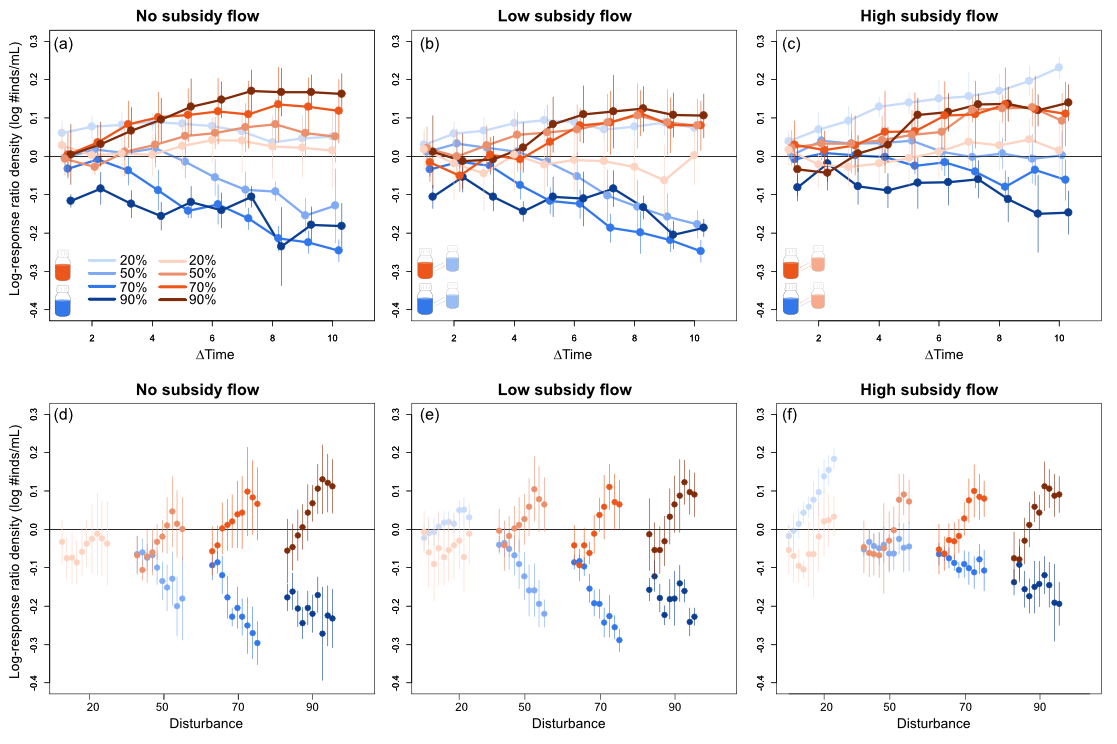


**Figure S1**: Log responses ratios of time series of log-transformed densities comparing each time point to T0 for (a) the isolated meta-ecosystems, (b) the low-resource connected ecosystems and (c) the high-resource connected ecosystems. Log response ratios of density comparing each treatment against the control (here: low resource isolated unconnected ecosystem) at each time point for (d) the isolated meta-ecosystems, (e) the low-resource connected ecosystems and (f) the high-resource connected ecosystems. Blue symbols are low-resource focal systems, while red symbols indicate high resource focal systems, both with varying levels of total perturbations (20%, 50%, 70%, 90%), respectively.


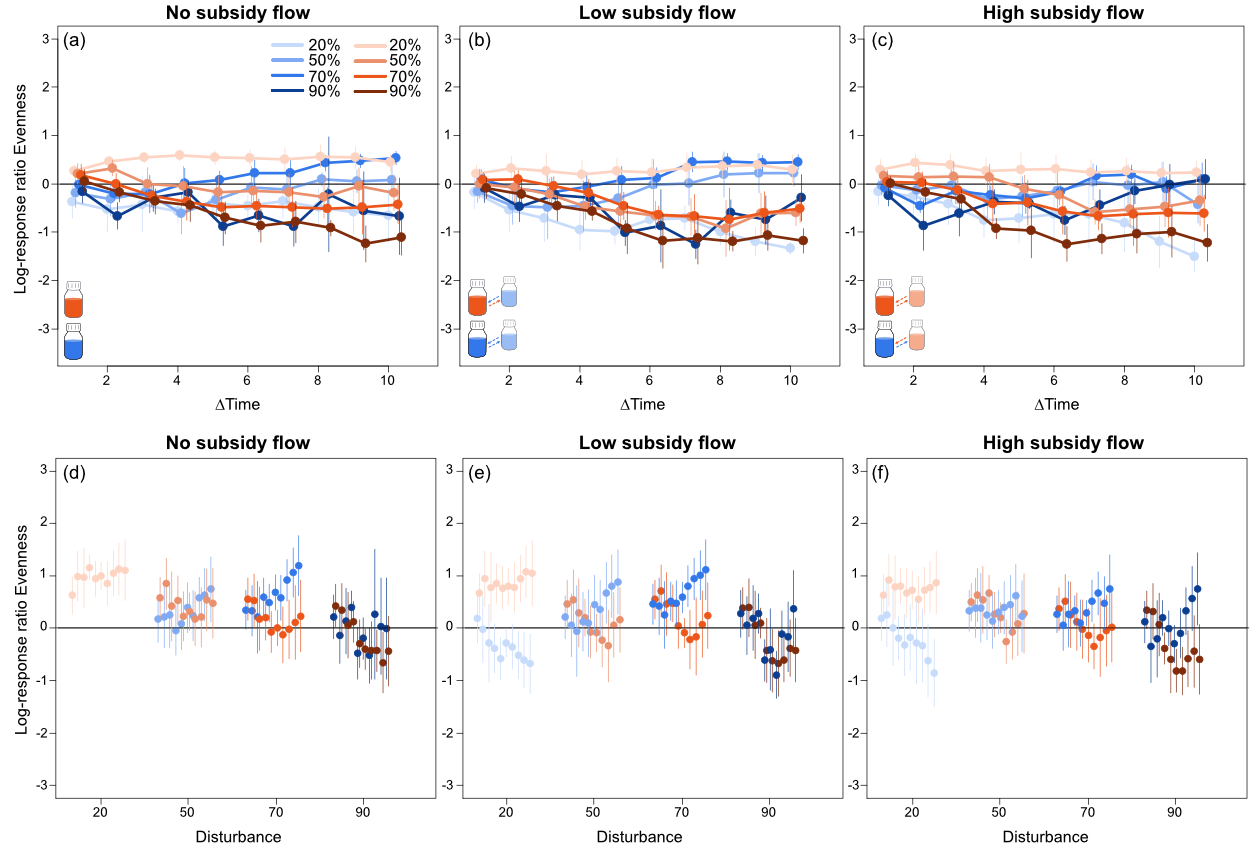


**Figure S2**: Log response ratios of time series of evenness comparing each time point to T0 for (a) the isolated ecosystems, (b) the low-resource connected ecosystems and (c) the high-resource connected ecosystems. Log response ratios of evenness comparing each treatment against the control (here taken as the low resource isolated ecosystem) at each time point for (d) the isolated ecosystems, (e) the low-resource connected ecosystems and (f) the high-resource connected ecosystems. Blue symbols are low resource focal systems, while red symbols indicate high resource focal systems, both with varying levels of total perturbations (20%, 50%, 70%, 90%), respectively.


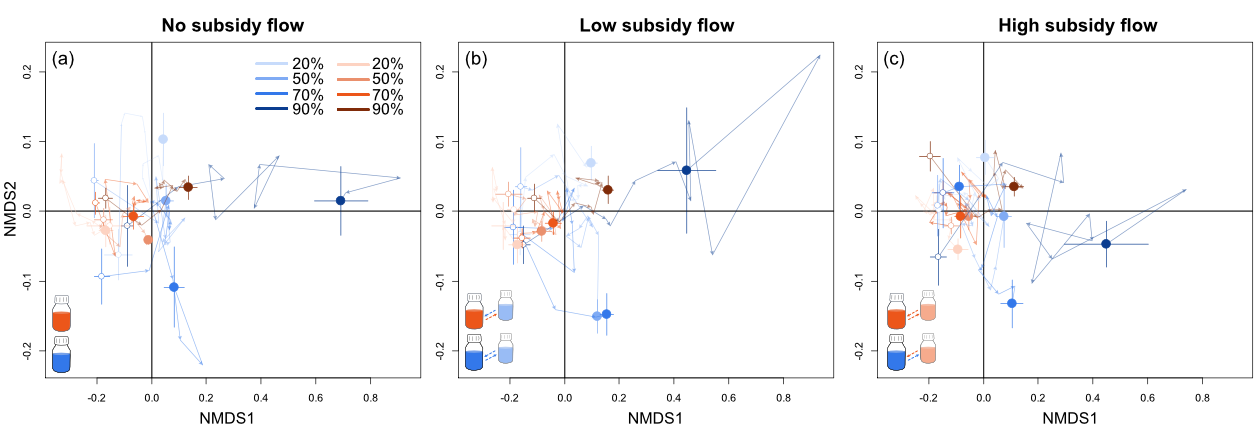


**Figure S3**: Visualization of community size class compositional trajectories from the start (unfilled symbols) to the end (filled symbols) of the experiment. We used Non-Metric Dimensional Scaling analysis on Hellinger-transformed size class abundances. Blue (resp. red) colors represent local low-resource (resp. high-resource) ecosystems with circles representing isolated ecosystems, squares representing ecosystems connected to a low-resource ecosystem and triangles representing ecosystems connected to a high-resource ecosystem. Color intensity reflects different disturbance intensity levels.
